# Trends and risk indicators for high fertility among Nigerian female youth aged 15–29 years: A pooled data analysis

**DOI:** 10.1016/j.heliyon.2024.e37946

**Published:** 2024-09-14

**Authors:** Turnwait Otu Michael, Soladoye S. Asa, Tope Olubodun

**Affiliations:** a Department of Sociology, University of Johannesburg, Gauteng, South Africa; b Department of Sociology, University of Ibadan, Ibadan, Nigeria; c Department of Demography and Social Statistics, Obafemi Awolowo University, Osun State, Nigeria; d Department of Community Medicine and Primary Care, Federal Medical Centre Abeokuta, Ogun State, Nigeria

**Keywords:** Children ever born, Female youth, Fertility, Marital status, Risk indicators

## Abstract

**Introduction:**

Nigeria's age-specific fertility rates are highest among the country's youth population, yet little is known about the factors that influence female youth fertility in Nigeria. This study examined fertility trends and risk factors associated with high fertility among Nigerian female youth aged 15 to 29.

**Methods:**

We examined a pooled data from four rounds of the Nigeria Demographic and Health Surveys (conducted in 2003, 2008, 2013, and 2018). Of the data, 62,713 female youth were extracted for this study. Descriptive statistics and multinomial logistic regression were employed.

**Results:**

The results show that 21.5 % of the sample had 3 or more children. Between 2003 and 2018, there was a drop of only 28 live births per 1000 women among the youth. Female youth with less than a secondary education were twice as likely to have 1–2 children (adjusted odds ratio [aOR] = 1.54, 95 % confidence interval [CI] = 1.43–1.66) and three times as likely to have 3 or more children (aOR = 3.32, 95 % CI = 3.03–3.64) compared to those with a secondary education or higher. Unmarried female youth were 95 % less likely to have 1–2 children and 98 % less likely to have 3 or more children. Additionally, female youth from the lowest-income families were twice as likely to have 1–2 children (aOR = 2.06, 95 % CI = 1.84–1.84) and four times as likely to have 3 or more children (aOR = 3.50, 95 % CI = 3.03–4.05) compared to those from the highest-income families.

**Conclusion:**

To control high fertility, significant improvement in education, employment, ending early marriage, poverty and unfavorable pronatalist cultural norms, are required.

## Highlights

• Educational disparities heighten fertility risks among female youth with less than secondary education.
• Income disparities significantly influence fertility outcomes, with lower-income female youth at higher risk.
• There is only a slight decrease in live births per 1000 women among Nigerian youth.
• Controlling high fertility among female youth requires addressing education, employment, early marriage, poverty, and culture.

## 1 Introduction

In 2024, the world population reached 8.1 billion, up from 8 billion in November 2022, and is projected to reach 9.8 billion by 2050 [1,2]. Nigeria, along with seven other countries (Congo, Egypt, Ethiopia, India, Pakistan, Philippines, and Tanzania), will account for more than 50 % of the increase in the world's population between 2024 and 2050 [3]. Nigeria currently has 229 million people [4], with women accounting for 49.2 % of its population [5], and young people aged 15–29 constituting 29 % of the population [6].

Nigeria had a fertility rate of 4.6 in 2022 [5], down from 5.3 in 2018, 5.5 in 2013, and 5.7 in 2008 and 2003 respectively [7]. This rate is higher than the fertility replacement level of 2.1 [8,9]. Behavioral and biological factors such as exposure, deliberate, and natural marital fertility contribute to the high fertility rate in Nigeria [10]. High fertility resulting from unintended pregnancies among female youth is linked to maternal and child health risks, truncated education, stigma, intergenerational poverty, and distorted social, economic, and health development [11,12]. Despite the high fertility rates among 15-19-year-olds and 25-29-year-olds [7], little is known about the trends and intermediate determinants of fertility among female youth in Nigeria. Prior studies on trends and determinants of fertility have been conducted among women aged 15–49 years [[13], [14], [15]], with limited understanding of the drivers of fertility among specific age groups that report high fertility rates. This study aims to address this gap by examining the trends and risk indicators for high fertility among Nigerian female youth aged 15–29 years utilizing a pooled data analysis from the IPUM-DHS datasets.

In achieving this objective, the study is based on two theories: Demographic Transition Theory (DTT) and Rational Choice Theory (RCT). Both theories were chosen for their ability to explain the interplay between socio-economic development and individual decision-making in shaping fertility patterns. In the Nigerian context, DTT helps elucidate the ongoing demographic shifts and persistent high fertility due to socio-economic factors, while RCT highlights how cultural norms, economic incentives, and access to resources influence reproductive choices among Nigerian female youth. DTT holds that as income, the economy, food supply, education, and technology improve, fertility will decline; and as medical and sanitation services improve, the death rate will decline [16]. DTT assumes that improving the social, economic, and intermediate factors contributing to high population growth rates in developing countries will reduce fertility [[16], [17], [18]]. RCT, on the other hand, acknowledges the contributing role of individuals to fertility because they make decisions based on cost-benefit analyses [19]. The theory explains why some people prefer high fertility despite access to social and economic factors that typically reduce fertility [19,20]. Prior studies in Nigeria have also demonstrated associations between marital status, religion, education, household size, and wealth index and fertility [12,21].

## 2 Methods

### 2.1 Data source

This study was a secondary analysis of data extracted from the IPUMS DHS database, available at [https://www.idhsdata.org/idhs/] [22]. Nigeria has six Nigeria Demographic and Health Survey (NDHS) datasets. The data for this study were extracted from the 2003, 2008, 2013, and 2018 NDHS datasets. Data from the NDHS 1999 was not accessible, and the data from the NDHS 1990 did not include the variables of interest. The pooled sample included 62,713 female respondents aged 15–29 years. The NDHS is a household cross-sectional nationally representative population-based survey conducted in the 774 local government areas in Nigeria. Pooling data from four survey rounds increased the sample size and precision of parameter estimates, allowing for better generalization of findings. It also provided independent estimates for each variable of interest, allowing for relative comparison by survey year. This approach was particularly relevant because the study's age-specific sample (15–29) consisted of female adolescents who had not yet completed their reproductive cycle. Using a single round of surveys would have made analyzing the fertility trends of this young female population difficult.

The NDHS employed a stratified sampling strategy that included two stages of cluster design. The first stage selected enumeration areas (EAs) as sampling units. The second stage listed households across selected EAs in both rural and urban areas to conduct a random sampling of eligible respondents at households for questionnaire administration. Data were collected using paper-based questionnaires in 2003, 2008, and 2013. In 2018, data were collected using computer-assisted personal interviewing (CAPI). The surveys' standardized DHS instrument reflected Nigeria's population and health situations. The survey questionnaires were translated into Hausa, Yoruba, and Igbo to facilitate understanding by respondents. The surveys collected information on respondents' demographics, household characteristics, fertility, family planning, sexual activity, and maternal and child health.

### 2.2 Study variables

**Dependent variable:** Female youth fertility (number of children ever born) was the dependent variable in this study. Total children ever born was coded as a continuous variable in the original NDHS dataset, ranging from 0 to 18 children. This variable was recoded into three categories: 0 = never born, 1 = born with 1–2 children, and 2 = born with 3 or more children. Given that the average number of children born by female Nigerian youth aged 15–29 years is 1.92 [7], this classification standard is appropriate. A similar analytical classification was used in a previous study [23]. Furthermore, because young people aged 15 to 29 have not yet reached the end of their reproductive years, it is necessary to have more than two categories to fully understand their fertility levels.

**Independent variable:** The explanatory variables are age (coded as 15–19 = 1, 20–24 = 2, 25–29 = 3), education (recoded as below secondary = 1, secondary+ = 2), marital status (not married = 0, married = 1), age at first marriage/cohabitation (below 18 = 1, 18+ = 2), religion (Christian = 1, Muslim/Other = 2), currently working (not working = 0, working = 1), wealth index (poorest = 1, poorer = 2, middle = 3, richer = 4, richest = 5). Other explanatory variables are ideal number of children (2 or less = 1, 3–4 = 2, 5+ = 3), ever had a pregnancy terminated (no = 0, yes = 1), FP use by method type (no method = 0, use method [traditional or modern] = 1), FP media exposure (not heard FP on radio, TV, or in newspaper/magazine = 0, heard from any of the three sources = 1), residence (urban = 1, rural = 2), ethnicity (Hausa = 1, Yoruba = 2, Igbo = 3, Other = 4), and year of sample (2003 = 1, 2008 = 2, 2013 = 3, 2018 = 4). The selection of the independent variables was influenced by previous research [10,13,24]. The wealth index from the DHS dataset was calculated in quintiles using Principal Component Analysis [25]. Respondents were asked about household wealth measuring items such as roofing materials, sanitary facilities, water sources, flooring materials, and household ownership of bicycles, cars, and televisions [7].

### 2.3 Statistical analyses

The data was analyzed at three different levels: descriptive (univariate), bivariate, and multivariate. Univariate analysis was used to examine the demographics of respondents (frequencies and percentages). The age-specific fertility rate (ASFR), which refers to the number of live births to women of a specific age group per 1,000, was calculated to support trend analysis on Nigerian female youth fertility. The age-specific fertility rate was calculated as ASFR(15–29) = B(15–29)/f_total population (15–29) x 1000. The formula used for each female age category, for example, females aged 15–19 years, was ASFR(15–19) = B(15–19)/f_total population (15–19) x 1000. In the formula, the symbol ‘B’ represents birth, while the symbol ‘f’ represents females. Rates in DHS data are for the 1–36 months preceding the survey [7]. The rate of decline in fertility rate for 15-29-year-olds was obtained by subtracting the total increase in live births per 1000 women from the total decline in live births per 1000 women between 2003 and 2018.

Bivariate analysis (chi-square) was used to test the association between variables at less than a 0.05 p-value to identify significant factors. This aided in the identification of variables that were statistically significant for inclusion in the multinomial regression model, as variables with no significant associations were excluded from the regression model stage. A diagnostic test was performed using variance inflation factor (VIF) to avoid multicollinearity errors in explanatory variables to further ensure that problems of collinearity are excluded from the regression model. The VIF diagnostic tests yielded a minimum VIF of 1.127 and a maximum VIF of 2.051, both of which are statistically appropriate and within an acceptable range.

A multinomial logistic regression model was used to test the predictive effect of the independent variables on the dependent outcome. The model used ‘never born’ as the reference category for the dependent variable, which was recoded into three categories (never born, born with 1–2 children, and born with 3 or more children). Age and education of husbands/partners were controlled. A matching variable method was utilized to eliminate confounding in the study design and to demonstrate an unbiased link between exposure and outcome variables. The matching variable strategy aided in achieving equal distribution of confounding variables among exposed and unexposed groups [26,27]. This eliminates the possibility that differences in confounding variables caused the variation in outcomes. This also allowed the model to estimate the number of children born versus never born. The data was analyzed using SPSS Statistics v25. To correct or adjust for disproportionate sampling, all surveys were weighted using the sample weight for persons (v005/1000000) on the women's file. Cases that were missing or had non-numeric responses were excluded from the analysis. Non-numeric responses cases on the ideal number of children, for example, were excluded from the analysis because they were difficult to quantify in relation to the number of children ever born, which requires quantified responses. "Up to God" and "don't know" are two examples of non-numerical responses. The missing cases, which included both user and system missing, were less than 1 % and insignificant, meaning that rather than employing an imputation technique which the nature of the datasets did not support, missing responses were dropped during analysis to avoid data misinterpretations and potential biases in discussions. Previous studies utilizing DHS datasets have also employed a similar technique in handling missing/non-numeric responses [28,29]. This manuscript adheres to the Strengthening the Reporting of Observational Studies in Epidemiology (STROBE) guidelines.

## 3 Results

Table 1 shows the frequency distribution of the socio-demographic characteristics of study participants whose data were extracted by survey year. The ages of the respondents were nearly evenly distributed across the categories in the four waves of surveys, with each category accounting for about 30 % of the sample distribution. Across the four surveys, nearly half of the respondents had a secondary education, while tertiary education was the least commonly reported. Less than 10 % of female youths had completed tertiary education. A little more than half of those surveyed were married. In the four waves of surveys, more than 60 % of married female youth were under the age of 18 at the time of their first marriage or cohabitation. In the 2003 and 2008 survey rounds, slightly more than half of the respondents were Christians, while slightly more than half of the respondents in the 2013 and 2018 survey years were Muslims or adherents of other religions. Over 50 % of respondents in the 2003, 2008, and 2013 survey rounds were not working, whereas 51.7 % were working in the 2018 survey year. Across the four waves of surveys, the poorest households had the fewest respondents. More than half of respondents across the four survey years said their ideal number of children was five or more, while less than 5 % said their ideal number of children was two or less.

**Table 1:** Frequency distribution of respondents’ characteristics (Weighted, N = 62,713).

**Variable**	**NDHS 2003** **Weighted (n = 4,170)**		**NDHS 2008** **Weighted (n = 16,486)**		**NDHS 2013** **Weighted (n = 20,094)**		**NDHS 2018** **Weighted (n = 21,961)**	
	**Frequency**	**%**	**Frequency**	**%**	**Frequency**	**%**	**Frequency**	**%**
**Age**								
15–19	1532	36.7	5607	34.0	7226	36.0	8202	37.4
20–24	1383	33.2	5349	32.5	6279	31.2	6686	30.4
25–29	1255	30.1	5530	33.5	6590	32.8	7073	32.2
**Education**								
None	1282	30.7	4339	26.3	6351	31.6	6783	30.9
Primary	827	19.8	2712	16.5	2661	13.2	2403	10.9
Secondary	1814	43.5	7992	48.5	9377	46.7	10730	48.9
Higher	247	5.9	1443	8.8	1706	8.5	2046	9.3
**Marital status**								
Not married	1961	47.0	7851	47.6	9154	45.6	10380	47.3
Married	2209	53.0	8636	52.4	10941	54.4	11582	52.7
**Age at 1**st **marriage/cohabitation**								
Below 18	1556	64.5	5514	59.6	7508	63.6	7749	61.9
18+	857	35.5	3742	40.4	4304	36.4	4761	38.1
**Religion**								
Christian	2236	53.6	9799	59.4	9701	48.3	9511	43.3
Muslim/Other	1934	46.4	6688	40.6	10394	51.7	12451	56.7
**Currently working**								
Not working	2307	55.3	8679	52.6	10452	52.0	10618	48.3
Working	1863	44.7	7807	47.4	9643	48.0	11343	51.7
**Wealth index**								
Poorest	653	15.7	2699	16.4	3311	16.5	3774	17.2
Poorer	716	17.2	2796	17.0	3922	19.5	4429	20.2
Middle	843	20.2	3104	18.8	3955	19.7	4462	20.3
Richer	889	21.3	3840	23.3	4298	21.4	4769	21.7
Richest	1068	25.6	4048	24.6	4608	22.9	4527	20.6
**Ideal No. of children**								
2 or less	84	2.0	638	3.9	510	2.5	1061	4.8
3–4	1254	30.1	6178	37.5	6824	34.0	7478	34.0
5+	2832	67.9	9671	58.7	12761	63.5	13423	61.1
**Total children ever born**								
0	2067	49.6	8216	49.8	9728	48.4	10563	48.1
1	743	17.8	2632	16.0	3383	16.8	3524	16.0
2	489	11.7	2118	12.8	2631	13.1	3085	14.0
3 or more	870	20.9	3520	21.4	4353	21.7	4790	21.8
**Ever had pregnancy terminated**								
No	3789	90.9	15293	92.8	18727	93.2	20307	92.5
Yes	381	9.1	1193	7.2	1368	6.8	1655	7.5
**FP use by method type**								
No method	3588	86.0	13936	84.5	17248	85.8	19748	89.9
Use method	582	14.0	2550	15.5	2847	14.2	2213	10.1
**FP media exposure**								
No	2357	56.5	9659	58.6	13188	65.6	15030	68.4
Yes	1813	43.5	6827	41.4	6907	34.4	6932	31.6
**Residence**								
Urban	1469	35.2	6286	38.1	8577	42.7	9710	44.2
Rural	2701	64.8	10200	61.9	11518	57.3	12252	55.8
**Ethnicity**								
Hausa/Fulani	1178	28.3	3758	22.8	6954	34.6	8601	39.2
Yoruba	495	11.9	3047	18.5	2644	13.2	2826	12.9
Igbo	622	14.9	2871	17.4	2993	14.9	3078	14.0
Other	1874	45.0	6809	41.3	7503	37.3	7457	34.0

Across the four survey years, approximately 48 % of female youth had never given birth, compared to approximately 21 % who had given birth to at least three children. Fewer than 10 % of those surveyed have ever had a pregnancy terminated. Over 80 % of respondents said they did not use any type of family planning to avoid unplanned pregnancies. More than half of those surveyed had heard family planning messages on the radio, television, or in newspapers/magazines. While more than half of the respondents lived in rural areas, approximately one-third of the respondents belonged to the Hausa or Fulani ethnic groups.

**Fig. 1:**
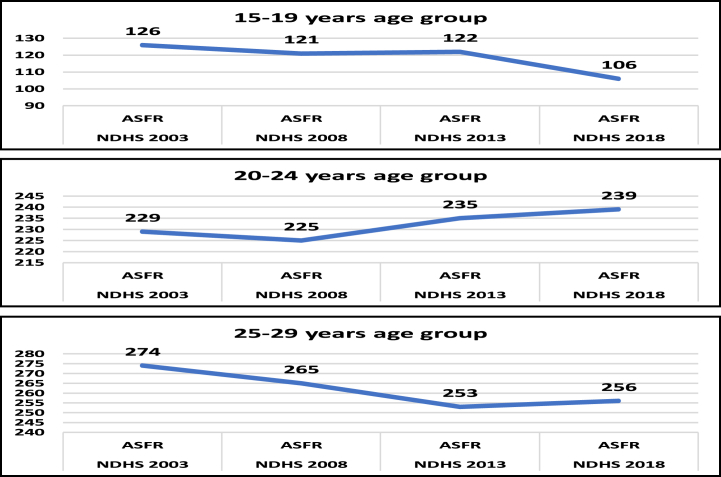
Trends in age-specific fertility rates for Nigerian female youth aged 15–29 years.

Fig. 1 depicts the trends of age-specific fertility rates among Nigerian female youth. The fertility rate for women aged 15–19 years decreased from 126 live births per 1000 women in 2003 to 106 live births per 1000 women in 2018, resulting in a fertility decline of 20 live births per 1000 women. For women aged 20–24 years, the fertility rate increased from 229 live births per 1000 women to 239 live births per 1000 women over the same period, resulting in a fertility increase of 10 live births per 1000 women. Among women aged 25–29 years, the fertility rate fell from 274 live births per 1000 women to 256 live births per 1000 women over the same period, resulting in a drop of 18 live births per 1000 women. However, the drop in fertility rates among women aged 25–29 was not steady: while there was a decline from 2003 to 2013, there was an increase from 253 live births per 1000 women in 2013 to 256 live births per 1000 women in 2018. Overall, there was a decline of only 28 live births per 1000 women among females aged 15–29 between 2003 and 2018.

Table 2 shows that 48.8 % of the female youth had never given birth, 29.7 % had 1-2 children, and 21.6 % had 3 or more children. Respondents aged 25–29 had three or more children more frequently than those aged 15–19. Those with less than a secondary education had more children than those with a secondary education or higher. Married female youth had more children than unmarried respondents. Respondents who married before the age of 18 had three or more children more frequently than those who married after the age of 18. Muslims and other religious adherents had more children than Christians. Female youth who were currently working had more children than their non-working counterparts. Female youth from the poorer and poorest households had more children than those from the richer and richest households. Respondents with an ideal number of five or more children had more children than others. Similarly, respondents with a preferred number of 3–4 children had a higher proportion of 1–2 children than those with an ideal number of five or more.

**Table 2:** Chi-square tests distribution of Nigerian female youth characteristics by fertility (number of children ever born), NDHS 2003–2018.

**Variable**	**Weighted (N = 62,713)**		**Number of children ever born**			**p-values**
	**N**	**%**	**Never born (%)**	**1**–**2 children (%)**	**3 or more children (%)**	
**Age**						<0.001
15-19	22,569	36.0	83.9	15.6	0.5	
20-24	19,695	31.4	40.6	43.2	16.2	
25-29	20,449	32.6	17.8	32.2	50.0	
**Education**						<0.001
Below secondary	27,358	43.6	27.9	35.6	36.5	
Secondary+	35,355	56.4	64.8	25.1	10.1	
**Marital status**						<0.001
Not married	29,346	46.8	87.6	10.1	2.3	
Married	33,367	53.2	14.6	46.9	38.5	
**Age at 1**st **marriage/cohabitation**						<0.001
Below 18 years	22,327	62.0	13.3	40.3	46.4	
18+	13,664	38.0	17.4	59.7	22.9	
**Religion**						
Christian	31,246	49.8	59.7	26.8	13.5	<0.001
Muslim/Other	31,467	50.2	37.9	32.5	29.6	
**Currently working**						<0.001
Not working	32,057	51.1	60.6	24.2	15.1	
Working	30,656	48.9	36.3	35.4	28.3	
**Wealth index**						<0.001
Poorest	10,437	16.6	31.1	34.8	34.1	
Poorer	11,863	18.9	37.3	33.2	29.5	
Middle	12,365	19.7	47.8	30.1	22.0	
Richer	13,796	22.0	55.7	27.6	16.7	
Richest	14,251	22.7	65.3	24.6	10.1	
**Ideal No. of children**						<0.001
2 or less	2292	3.7	61.3	23.6	15.1	
3-4	21,734	34.7	63.7	28.0	8.4	
5+	38,687	61.7	39.6	31.0	29.4	
**Ever had pregnancy terminated**						<0.001
No	58,116	92.7	50.7	28.6	20.7	
Yes	4597	7.3	24.0	42.7	33.3	
**FP use by method type**						<0.001
No method	54,520	86.9	49.3	29.1	21.5	
Use method	8192	13.1	44.8	33.2	21.9	
**FP media exposure**						<0.001
No	40,234	64.2	47.3	29.6	23.1	
Yes	22,479	35.8	51.3	29.8	18.9	
**Residence**						<0.001
Urban	26,042	41.5	59.3	25.9	14.8	
Rural	36,671	58.5	41.3	32.3	26.4	
**Ethnicity**						
Hausa/Fulani	20,492	32.7	33.4	33.3	33.3	<0.001
Yoruba	9013	14.4	60.5	29.1	10.3	
Igbo	9565	15.3	64.8	22.2	13.0	
Other	23,643	37.7	51.0	29.8	19.2	
**Year of sample**						
2003	4170	6.6	49.6	29.6	20.9	<0.022
2008	16,486	26.3	49.8	28.8	21.4	
2013	20,094	32.0	48.4	29.9	21.7	
2018	21,961	35.0	48.1	30.1	21.8	

Note: FP – family planning; n – weighted count; % - weighted percentage.

Female youth who have had a pregnancy terminated had more children than those who had never had a pregnancy terminated. Conversely, respondents who had never had a pregnancy terminated had a higher proportion of never-born children than those who had. Female youth who used family planning (either modern or traditional methods) had a higher proportion of 1–2 children than those who did not, but a nearly equal proportion of 3 or more children compared to those who did not use family planning. Respondents who had no exposure to family planning media had more children than those who had exposure to family planning media. Female youth in rural areas had more children than those in urban areas. Respondents from the Hausa/Fulani ethnic group had more children than those from other ethnic groups. Female youth respondents in the 2003 survey year had fewer instances of having 3 or more children compared to those in successive survey years. Similarly, respondents in the 2003 and 2008 survey years had a higher proportion of never-born children than those in subsequent survey years.

Table 3 presents the results of the multinomial logistic regression analysis. The analysis indicates significant associations between the number of children (1–2 children and 3 or more children) and factors such as age, education, marital status, wealth index, residence, ethnicity, and survey year, when compared to respondents who have never had children. Employment status was significantly associated with having 3 or more children but not with having 1–2 children compared to respondents who had never had children. Additionally, religion was significantly related to having 1–2 children but not to having 3 or more children. Female youth aged 15–19 were 88 % less likely than those aged 25–29 to have 1–2 children and 99 % less likely to have 3 or more children compared to those who had never given birth. Respondents with less than a secondary education were twice as likely as those with a secondary education or higher to have 1–2 children and three times as likely to have 3 or more children. Unmarried female youth were 95 % less likely to have 1–2 children and 98 % less likely to have 3 or more children compared to never born respondents.

**Table 3:** Results of multinomial logistic regression showing Nigerian female youth fertility (number of children ever born) by selected variables, NDHS 2003–2018.

Variable	1-2 children vs. never born		3 or more children vs. never born	
	Adjusted Odds Ratio	95 % C.I.	Adjusted Odds Ratio	95 % C.I.
**Age**				
15–19	0.119∗∗∗	0.110-0.129	0.001∗∗∗	0.001-0.002
20–24	0.655∗∗∗	0.612-0.701	0.116∗∗∗	0.108-0.126
25–29	RC		RC	
**Education**				
Below secondary	1.537∗∗∗	1.426-1.656	3.320∗∗∗	3.026-3.642
Secondary+	RC		RC	
**Marital status**				
Not married	0.046∗∗∗	0.043-0.049	0.024∗∗∗	0.022-0.027
Married	RC		RC	
**Religion**				
Christian	1.454∗∗∗	1.340-1.578	0.983	0.887-1.090
Muslim/Other	RC		RC	
**Currently working**				
Not working	0.701	0.664-0.740	0.557∗∗∗	0.520-0.597
Working	RC		RC	
**Wealth index**				
Poorest	2.063∗∗∗	1.842-2.311	3.502∗∗∗	3.029-4.047
Poorer	2.006∗∗∗	1.807-2.227	3.435∗∗∗	3.000–3.933
Middle	2.132∗∗∗	1.945-2.337	3.451∗∗∗	3.056-3.896
Richer	1.694∗∗∗	1.563-1.837	2.516∗∗∗	2.259-2.802
Richest	RC		RC	
**Residence**				
Urban	1.007	0.941-1.078	0.987	0.905-1.076
Rural	RC		RC	
**Ethnicity**				
Hausa/Fulani	0.892∗∗	0.820-0.970	1.177∗∗	1.063-1.303
Yoruba	1.068	0.979-1.164	0.548∗∗∗	0.487-0.618
Igbo	0.637∗∗∗	0.585-0.694	0.744∗∗∗	0.666-0.832
Other	RC		RC	
**Year of sample**				
2003	0.856∗∗	0.768-0.954	0.891	0.776-1.024
2008	0.788∗∗∗	0.736-0.844	0.866∗∗	0.794-0.944
2013	0.862∗∗∗	0.809-0.919	0.845∗∗∗	0.779-0.916
2018	RC		RC	
Model chi-square	57460.594∗∗∗			
−2 Log Likelihood	16795.994			
Cox & Snell Pseudo R^2^	0.600			
Nagelkerke Pseudo R^2^	0.685			
Classification overall % (correct)	74.5 %			
N	62,713			

Significant at p < .05∗, p < .01∗∗, p < .001∗∗∗; RC – reference category; CI – confidence interval.

Christian respondents were 1.5 % more likely to have 1–2 children than Muslims or other religious adherents. Although not statistically significant, Christian respondents were 1.7 % less likely to have 3 or more children than Muslims or other religious adherents, indicating that Muslims or other religious adherents were more likely to have three or more children than Christians. Respondents who were not working were 44 % less likely to have 3 or more children than those who were working, suggesting that the nature of the job might influence fertility, especially in family businesses not paid in cash or in-kind. As household wealth index rises, the number of children decreases. Female youth from the poorest households were twice as likely as those from the richest households to have 1–2 children and four times as likely to have 3 or more children. Respondents from the Hausa/Fulani ethnic group were 11 % less likely to have 1–2 children but 1.2 times more likely to have three or more children than those from "Other" ethnic groups. Igbo respondents were 36 % less likely to have 1–2 children and 26 % less likely to have 3 or more children than those from the "Other" ethnic group. Similarly, respondents from the Yoruba ethnic group were 45 % less likely to have 3 or more children than those from the "Other" ethnic group when compared to never born respondents.

The results indicate that fertility among Nigerian female youth aged 15–29 has not significantly declined over the years. For instance, in 2003, Nigerian female youth were 14 % less likely to have 1–2 children than in 2018. In 2008, they were 21 % less likely to have 1–2 children and 13 % less likely to have 3 or more children than in 2018. Similarly, in 2013, Nigerian female youth were 14 % less likely to have 1–2 children and 16 % less likely to have 3 or more children than in 2018.

## 4 Discussion

We investigated the trends and determinants of fertility among Nigerian female youth using pooled NDHS datasets from 2003 to 2018. Our study found that the age-specific fertility rate among Nigerian female youth aged 15-19 declined by 20 live births per 1000 women, from 126 to 106 over the 15-year period. Previous research has also indicated a slow decline in female fertility in Nigeria at age-specific fertility rates [30,31]. In contrast, the number of live births to women aged 20–24 increased from 229 per 1000 women to 239 per 1000 women, showing a fertility rise of 10 per 1000 women. This suggests that not all age-specific fertility categories in Nigeria experienced a decline over the 15-year period, consistent with findings that fertility increases with age in some categories [7,32].

Fertility among women aged 25–29 years fell from 274 to 256 live births per 1000 women, a decrease of 18 live births per 1000 women over the 15-year period. However, this decline was not steady; there was a brief increase from 253 to 256 live births per 1000 women. Previous studies found that fertility peaks between the ages of 25 and 29 in Nigeria [7,33]. Cultural beliefs and early marriage contribute to this pattern, as Nigerian females rarely delay childbearing [5]. Overall, there was a net decrease of 28 live births per 1000 women between 2003 and 2018. Previous research has indicated that pronatalist attitudes in Nigeria have contributed to a slow demographic transition, increasing the country's population [21,34].

Our study also revealed that female youth who had terminated pregnancies had more children than those who had not. Conversely, those who had never terminated a pregnancy had a higher proportion of never having children. This finding differs from a study of nine Sub-Saharan countries, which found lower odds of pregnancy termination among young women with higher numbers of births [35]. Additionally, we found that female youth who use family planning methods have a higher proportion of 1–2 children than those who do not, but a nearly equal proportion of 3 or more children compared to non-users. This is supported by previous research indicating lower than expected family planning utilization in Nigeria [36,37]. Traditional family planning methods are less effective and may contribute to unplanned pregnancies [7,38]. Respondents with no exposure to family planning media had more children than those who had exposure. Additionally, rural female youth had more children than urban female youth, consistent with findings that rural areas in Nigeria have higher fertility rates and lower access to modern family planning [5,37].

Age, education, marital status, wealth index, residence, ethnicity, and survey year were significantly associated with having 1–2 children and 3 or more children compared to respondents who had never had children. Employment status was significantly associated with having 3 or more children but not with having 1–2 children. Religion was significantly associated with having 1–2 children but not with having 3 or more children. Female youth aged 15–19 were 88 % less likely than those aged 25–29 to have 1–2 children and 99 % less likely to have 3 or more children. Those with less than a secondary education were twice as likely to have 1–2 children and three times as likely to have 3 or more children, suggesting that timely educational advancement reduces fertility among young women [34,39].

Unmarried female youth were 95 % less likely to have 1–2 children and 98 % less likely to have 3 or more children compared to never born respondents. Previous research found 29 % non-marital fertility among Nigerian women, indicating a need to address high fertility rates among unmarried females [40]. Unlike Nigeria, where fertility is high, a study in Japan and East Asia found that marriage continues to shape fertility despite demographic transition and development [41]. Christians were 1.5 % more likely than Muslims or other religious adherents to have 1–2 children, and though not statistically significant, Christians were 1.7 % less likely to have 3 or more children. This aligns with previous research indicating that Muslim women in Nigeria have more children and are more likely to discontinue family planning than Christians [36,42].

Respondents who did not work were 44 % less likely to have 3 or more children than those who did. The nature of the job may help understand fertility situations, as some female youth jobs are family businesses not paid in cash or in-kind [43]. A study in 21 European countries found a strong relationship between GDP per capita and fertility levels [44]. As the household wealth index rises, the number of children falls. Female youth from poorer households were twice as likely to have 1–2 children and four times as likely to have 3 or more children compared to those from wealthier households. A study in Timor-Leste found similar results, with young females from poor households more likely to have more children than those from wealthy households [27]. Previous studies in Nigeria also found that a higher wealth index is associated with lower fertility rates [5,30].

Respondents from the Hausa/Fulani ethnic group were 11 % less likely to have 1–2 children but 20 % more likely to have 3 or more children than those from "Other" ethnic groups. Igbo respondents were 36 % less likely to have 1–2 children and 26 % less likely to have 3 or more children than "Other" respondents. Yoruba respondents were 45 % less likely to have 3 or more children than "Other" respondents. Previous studies found that the Hausa-Fulani ethnic group has the highest fertility rate, followed by the Yoruba and Igbo ethnic groups [45]. Similar findings were observed in Ghana [46].

Our study shows that fertility among Nigerian female youth aged 15–29 is declining haphazardly rather than steadily year after year. For example, in 2003, Nigerian female youth were 14 % less likely than in 2018 to have 1–2 children. In 2008, they were 21 % less likely to have 1–2 children and 13 % less likely to have 3 or more children than in 2018. Similarly, in 2013, they were 14 % less likely to have 1–2 children and 16 % less likely to have 3 or more children than in 2018. Previous research has found an increase in fertility among Nigerian women due to the country's growing population, low contraceptive use, and poverty [11,30,40].

Certain factors could explain these inconsistencies in fertility declines in Nigeria. Socio-economic and political instability, such as economic recessions, policy changes, disrupt access to education, healthcare, and family planning services, thereby affecting fertility rates [47,48]. Cultural and religious influences on family size preferences and contraceptive use vary across regions and over time, contributing to the irregular patterns observed. Fluctuations in international aid and government funding for reproductive health programs also lead to inconsistent availability and quality of family planning services [49]. Understanding these underlying causes is crucial for formulating more effective interventions that address the diverse and dynamic factors influencing fertility rates among Nigerian female youth.

The findings of our study are further elucidated through the lenses of two theories employed. According to Demographic Transition Theory (DTT), societies transition from high fertility and mortality rates to lower rates as they develop economically and socially. This transition typically progresses through three to five stages, with fertility rates declining due to increased access to education, improved healthcare, and greater socioeconomic opportunities, particularly for women [[16], [17], [18]]. However, our study indicates that Nigerian female youth, especially those with less education, from poorer households, or living in rural areas, continue to exhibit high fertility rates. This suggests that Nigeria may still be in the early stages of this demographic transition, where traditional norms and limited access to family planning resources maintain high fertility rates despite some socio-economic advancements.

Rational Choice Theory (RCT) posits that individuals make reproductive choices based on a rational evaluation of costs and benefits [19,20]. The study's findings that married female youth, those from poorer households, and those with lower educational attainment have more children align with RCT. These groups may perceive higher economic and social benefits from larger families, such as labor contributions to household income or enhanced social status. Additionally, the limited use of family planning methods and high ideal number of children reflect a rational response to contextual factors such as cultural expectations, economic needs, and access to resources. In essence, both DTT and RCT provide frameworks to understand the persistence of high fertility rates among Nigerian female youth, emphasizing the interplay of socio-economic conditions, cultural norms, and individual rationality in shaping reproductive behavior.

### 4.1 Implications for policy and research

The study's findings have significant implications for both policy and research aimed at addressing high fertility rates among Nigerian female youth. From a policy perspective, the findings highlight the urgent need for targeted interventions to improve education and economic opportunities for young women, particularly in rural areas and among poorer households. Policies that promote secondary and tertiary education for girls, delay early marriages, and enhance access to family planning services are crucial. Expanding comprehensive sex education and increasing the availability of reproductive health services can empower young women to make informed choices about their fertility. Moreover, addressing socio-economic disparities by improving job opportunities and economic conditions for women can reduce the perceived need for larger families as a source of economic security.

From a research perspective, there is a need for deeper investigation into the socio-cultural and economic factors that sustain high fertility rates despite the observed demographic transitions. Future research should explore the role of cultural norms, access to education, and economic opportunities in shaping reproductive behavior among young women in Nigeria. Additionally, longitudinal studies could provide insights into the long-term effects of early marriage and low educational attainment on fertility patterns, helping to identify critical intervention points for reducing high fertility rates. By implementing these targeted strategies, policymakers, demographers and public health practitioners can effectively address the high fertility rates among Nigerian female youth, ultimately contributing to broader socio-economic development and improved health outcomes for women and their families.

### 4.2 Strengths and limitations

This study used a nationally representative sample that collected data using standardized, culturally sensitive instruments, which improved data quality and generalizability of the study's findings. One of the flaws of the study is the risk of response bias by women who may be unwilling to provide accurate information on the exact number of children ever born due to personal or cultural beliefs. This response bias could lead to underreporting or overreporting of fertility rates, potentially skewing the results. To mitigate this, efforts were made to ensure privacy and confidentiality during data collection, and interviewers were trained to build rapport and trust with respondents. Another limitation of the study is the assumption that women's socio-demographic characteristics remained unchanged during the period of giving birth. This assumption undermines the validity of associating the number of children ever born with individual and household characteristics, as these characteristics can change over time and significantly affect fertility rates. For instance, a woman's educational attainment, marital status, or economic situation may evolve, influencing her reproductive decisions. Recognizing this, future research should consider longitudinal designs to capture these dynamic changes, and interview the same respondents to provide a more accurate analysis. Additionally, the data was extrapolated from cross-sectional surveys, limiting the ability to make causal inferences about the relationships between socio-demographic factors and fertility rates. Despite these shortcomings, the findings of this study are useful in shaping policies and designing programs that can address the high fertility rates among young females in Nigeria, providing valuable insights into the socio-demographic factors influencing reproductive behavior.

## 5 Conclusions

There is slow and inconsistent decline in fertility among young people aged 15–29 in Nigeria. Risk factors associated with higher fertility rates include low education, early marriage, unemployment, poverty, and belonging to the Hausa/Fulani ethnicity. Given that the peak of age-specific fertility rates in Nigeria occurs among youth, it is imperative for the government and civil society organizations to implement targeted strategies. These strategies should focus on enhancing education, ending early marriage, reducing youth unemployment, and alleviating youth poverty. Moreover, effective communication channels including indigenous languages, pidgin, and English should be utilized to promote comprehensive sexual and reproductive health education among young people. This effort aims to prevent unplanned pregnancies and abortions. A sensitization program should particularly encourage married young women to have a number of children they can economically support. It is recommended to advocate for a norm of two children of any gender. Clear explanations should be provided to help female youths understand that having numerous children may not be financially sustainable in today's society, where parents must invest significant resources in their children's education from primary through university levels. To address uncontrollable high fertility rates in Nigeria, collaboration is needed among church and Islamic clerics, community leaders, and government agencies. Their joint efforts can effectively advocate for and promote initiatives aimed at reducing fertility rates across the country.

## Funding statement

This research did not receive any specific grant from funding agencies in the public, commercial, or not-for-profit sectors.

## Data availability statement

The data used for this study are publicly available from the IPUMS DHS Program. Interested researchers can register on the DHS Program website (https://www.idhsdata.org/idhs-action/extract_requests/download) and request access to the datasets. The raw data that support the findings of this study are available from the corresponding author upon reasonable request.

## Ethics declarations

This study protocol was reviewed and approved by the National Health Research Ethics Committee of Nigeria (NHREC) and the ICF Institutional Review Board, with the approval number: ICF IRB FWA00000845. All participants provided informed consent to participate in the study and for their data to be published. The authors obtained written permission from the IPUMS DHS Program to use the DHS data in this study. No additional ethics approval was required for this study.

**Acknowledgements**

We are grateful to IPUMS DHS for providing access to the DHS datasets used in this study. This article is an output of the SPARKLE Fellowship of the University of Medical Sciences, which was funded by the 10.13039/100000010Ford Foundation. The authors also acknowledge the technical leadership of the SPARKLE (Strengthening Programming for Adolescents and Youths through Resource and Knowledge Generation and Link to Evidence) in persons of Prof Adesegun Fatusi and Dr Olorunfemi Ogundele towards the capacity building of the first author and conceptualisation of the research agenda.

## CRediT authorship contribution statement

**Turnwait Otu Michael:** Writing – review & editing, Writing – original draft, Visualization, Validation, Supervision, Software, Resources, Project administration, Methodology, Investigation, Formal analysis, Data curation, Conceptualization. **Soladoye S. Asa:** Writing – review & editing, Validation, Supervision, Resources, Methodology, Conceptualization. **Tope Olubodun:** Writing – review & editing, Validation, Supervision, Resources, Methodology, Investigation, Conceptualization.

## Declaration of competing interest

The authors declare that they have no known competing financial interests or personal relationships that could have appeared to influence the work reported in this paper.
