# Supplementary material for: Trends and risk indicators for high fertility among Nigerian female youth aged 15–29 years: A pooled data analysis
Source: Heliyon. 2024 Sep 14;10(18):e37946. doi: 10.1016/j.heliyon.2024.e37946 (PMC11421995; doi:10.1016/j.heliyon.2024.e37946)
Supplement: Multimedia component 1 [file mmc1.pdf]

FORMATTING DATE: 07 Jun 2017
ENGLISH LANGUAGE: 26 May 2018

NIGERIA DEMOGRAPHIC AND HEALTH SURVEY - 2018
WOMAN'S QUESTIONNAIRE

NIGERIA
NATIONAL POPULATION COMMISSION

IDENTIFICATION

STATE ______

LOCAL GOVT. AREA ______

LOCALITY ______

ENUMERATION AREA ______

NAME OF HOUSEHOLD HEAD ______

CLUSTER NUMBER . . . .

HOUSEHOLD NUMBER . . . .

NAME AND LINE NUMBER OF WOMAN ______

CHECK COVER PAGE OF HOUSEHOLD QUESTIONNAIRE: HOUSEHOLD SELECTED FOR MAN'S SURVEY? (1=YES, 2=NO)

CHECK HOUSEHOLD QUESTIONNAIRE DVH01: WOMAN SELECTED FOR DV MODULE? (1=YES, 2=NO)

INTERVIEWER VISITS

	1	2	3	FINAL VISIT
DATE				DAY<br>MONTH<br>YEAR
INTERVIEWER'S NAME				INT. NO.
RESULT*				RESULT*
NEXT VISIT: DATE<br>TIME				TOTAL NUMBER OF VISITS

*RESULT CODES: 1 COMPLETED, 2 NOT AT HOME, 3 POSTPONED, 4 REFUSED, 5 PARTLY COMPLETED, 6 INCAPACITATED, 7 OTHER ______ (SPECIFY)

LANGUAGE OF QUESTIONNAIRE** 0 1

LANGUAGE OF INTERVIEW**

NATIVE LANGUAGE OF RESPONDENT**

TRANSLATOR USED (YES = 1, NO = 2)

LANGUAGE OF QUESTIONNAIRE** **ENGLISH**

**LANGUAGE CODES:
01 ENGLISH
02 HAUSA
03 YORUBA
04 IGBO

SUPERVISOR	FIELD EDITOR
NAME ______ NUMBER	NAME ______ NUMBER

INTRODUCTION AND CONSENT

Hello. My name is ________________________. I am working with National Population Commission. We are conducting a survey about health and other topics all over Nigeria. The information we collect will help the government to plan health services. Your household was selected for the survey. The questions usually take about 30 to 60 minutes. All of the answers you give will be confidential and will not be shared with anyone other than members of our survey team. You don't have to be in the survey, but we hope you will agree to answer the questions since your views are important. If I ask you any question you don't want to answer, just let me know and I will go on to the next question or you can stop the interview at any time.

In case you need more information about the survey, you may contact the person listed on the card that has already been given to your household.

Do you have any questions?
May I begin the interview now?

SIGNATURE OF INTERVIEWER ________________________ DATE ______________

RESPONDENT AGREES TO BE INTERVIEWED . . 1 ↓

RESPONDENT DOES NOT AGREE TO BE INTERVIEWED . . 2 → END

SECTION 1. RESPONDENT'S BACKGROUND

NO.	QUESTIONS AND FILTERS	CODING CATEGORIES	SKIP
101	RECORD THE TIME.	HOURS . . . . [ ][ ]<br>MINUTES . . . . [ ][ ]	
102	How long have you been living continuously in (NAME OF CURRENT CITY, TOWN OR VILLAGE OF RESIDENCE)?<br><br>IF LESS THAN ONE YEAR, RECORD '00' YEARS.	YEARS . . . . [ ][ ]<br><br>ALWAYS . . . . 95<br>VISITOR 96	 <br>95, 96 → 105
103	Just before you moved here, did you live in a city, in a town, or in a rural area?	CITY . . . . 1<br>TOWN . . . . 2<br>RURAL AREA 3	
104	Before you moved here, which state did you live in?	ABIA . . . . 01<br>ADAMAWA . . . . 02<br>AKWA IBOM . . . . 03<br>ANAMBRA . . . . 04<br>BAUCHI . . . . 05<br>BAYELSA . . . . 06<br>BENUE . . . . 07<br>BORNO . . . . 08<br>CROSS RIVER . . . . 09<br>DELTA . . . . 10<br>EBONYI . . . . 11<br>EDO . . . . 12<br>EKITI . . . . 13<br>ENUGU . . . . 14<br>FCT-ABUJA . . . . 15<br>GOMBE . . . . 16<br>IMO . . . . 17<br>JIGAWA . . . . 18<br>KADUNA . . . . 19<br>KANO . . . . 20<br>KATSINA . . . . 21<br>KEBBI . . . . 22<br>KOGI . . . . 23<br>KWARA . . . . 24<br>LAGOS . . . . 25<br>NASARAWA . . . . 26<br>NIGER . . . . 27<br>OGUN . . . . 28<br>ONDO . . . . 29<br>OSUN . . . . 30<br>OYO . . . . 31<br>PLATEAU . . . . 32<br>RIVERS 33	

SECTION 1. RESPONDENT'S BACKGROUND

NO.	QUESTIONS AND FILTERS	CODING CATEGORIES	SKIP
		SOKOTO . . . . . 34<br>TARABA . . . . . 35<br>YOBE . . . . . 36<br>ZAMFARA . . . . . 37<br><br>OUTSIDE OF NIGERIA 96	
105	In what month and year were you born?	MONTH . . . . . [ ][ ]<br><br>DON'T KNOW MONTH . . . . . 98<br><br>YEAR . . . . . [ ][ ][ ][ ]<br><br>DON'T KNOW YEAR 9998	
106	How old were you at your last birthday?<br><br>COMPARE AND CORRECT 105 AND/OR 106 IF INCONSISTENT.	AGE IN COMPLETED YEARS . . . . . [ ][ ]	
107	Have you ever attended school?	YES . . . . . 1<br>NO 2	 → 111
108	What is the highest level of school you attended: primary, secondary, or higher?	PRIMARY . . . . . 1<br>SECONDARY . . . . . 2<br>HIGHER 3	
109	What is the highest (class/year) you completed at that level?<br><br>IF COMPLETED LESS THAN ONE YEAR AT THAT LEVEL, RECORD '00'.	CLASS/YEAR . . . . . [ ][ ]	
110	CHECK 108:<br>PRIMARY OR SECONDARY [ ] ↓	HIGHER [ ]	→ 113
111	Now I would like you to read this sentence to me.<br><br>SHOW CARD TO RESPONDENT.<br><br>IF RESPONDENT CANNOT READ WHOLE SENTENCE,<br>PROBE: Can you read any part of the sentence to me?	CANNOT READ AT ALL . . . . . 1<br>ABLE TO READ ONLY PART OF THE SENTENCE . . . . . 2<br>ABLE TO READ WHOLE SENTENCE . . . . . 3<br>NO CARD WITH REQUIRED LANGUAGE ______ 4<br>(SPECIFY LANGUAGE)<br>BLIND/VISUALLY IMPAIRED 5	

SECTION 1. RESPONDENT'S BACKGROUND

NO.	QUESTIONS AND FILTERS	CODING CATEGORIES	SKIP
112	CHECK 111:<br>CODE '2', '3' OR '4' CIRCLED ↓<br>CODE '1' OR '5' CIRCLED		→114
113	Do you read a newspaper or magazine at least once a week, less than once a week or not at all?	AT LEAST ONCE A WEEK . . . . 1<br>LESS THAN ONCE A WEEK . . . . 2<br>NOT AT ALL 3	
114	Do you listen to the radio at least once a week, less than once a week or not at all?	AT LEAST ONCE A WEEK . . . . 1<br>LESS THAN ONCE A WEEK . . . . 2<br>NOT AT ALL 3	
115	Do you watch television at least once a week, less than once a week or not at all?	AT LEAST ONCE A WEEK . . . . 1<br>LESS THAN ONCE A WEEK . . . . 2<br>NOT AT ALL 3	
116	Do you own a mobile telephone?	YES . . . . 1<br>NO 2	 →118
117	Do you use your mobile phone for any financial transactions?	YES . . . . 1<br>NO 2	
118	Do you have an account in a bank or other financial institution that you yourself use?	YES . . . . 1<br>NO 2	
119	Have you ever used the internet?	YES . . . . 1<br>NO 2	 →122
120	In the last 12 months, have you used the internet?<br><br>IF NECESSARY, PROBE FOR USE FROM ANY LOCATION, WITH ANY DEVICE.	YES . . . . 1<br>NO 2	 →122
121	During the last one month, how often did you use the internet: almost every day, at least once a week, less than once a week, or not at all?	ALMOST EVERY DAY . . . . 1<br>AT LEAST ONCE A WEEK . . . . 2<br>LESS THAN ONCE A WEEK . . . . 3<br>NOT AT ALL 4	
122	What is your religion?	CATHOLIC . . . . 1<br>OTHER CHRISTIAN . . . . 2<br>ISLAM . . . . 3<br>TRADITIONALIST . . . . 4<br><br>OTHER ______________________ 6<br>(SPECIFY)	
123	What is your ethnic group?	______________________ ☐☐☐<br>(ETHNIC GROUP)	
124	In the last 12 months, how many times have you been away from home for one or more nights?	NUMBER OF TIMES . . . . ☐☐<br><br>NONE 00	 <br>→201
125	In the last 12 months, have you been away from home for more than one month at a time?	YES . . . . 1<br>NO 2	

SECTION 2. REPRODUCTION

NO.	QUESTIONS AND FILTERS	CODING CATEGORIES	SKIP
201	Now I would like to ask about all the births you have had during your life. Have you ever given birth?	YES . . . . 1<br>NO 2	 → 206
202	Do you have any sons or daughters to whom you have given birth who are now living with you?	YES . . . . 1<br>NO 2	 → 204
203	a) How many sons live with you?<br>b) And how many daughters live with you?<br>IF NONE, RECORD '00'.	a) SONS AT HOME . . . . [ ][ ]<br>b) DAUGHTERS AT HOME . . . . [ ][ ]	
204	Do you have any sons or daughters to whom you have given birth who are alive but do not live with you?	YES . . . . 1<br>NO 2	 → 206
205	a) How many sons are alive but do not live with you?<br>b) And how many daughters are alive but do not live with you?<br>IF NONE, RECORD '00'.	a) SONS ELSEWHERE . . . . [ ][ ]<br>b) DAUGHTERS ELSEWHERE . . . . [ ][ ]	
206	Have you ever given birth to a boy or girl who was born alive but later died?<br>IF NO, PROBE: Any baby who cried, who made any movement, sound, or effort to breathe, or who showed any other signs of life even if for a very short time?	YES . . . . 1<br>NO 2	 → 208
207	a) How many boys have died?<br>b) And how many girls have died?<br>IF NONE, RECORD '00'.	a) BOYS DEAD . . . . [ ][ ]<br>b) GIRLS DEAD . . . . [ ][ ]	
208	SUM ANSWERS TO 203, 205, AND 207, AND ENTER TOTAL. IF NONE, RECORD '00'.	TOTAL BIRTHS . . . . [ ][ ]	
209	CHECK 208:<br>Just to make sure that I have this right: you have had in TOTAL _____ births during your life. Is that correct?<br>YES [ ] ↓ NO [ ] → PROBE AND CORRECT 201-208 AS NECESSARY.		
210	CHECK 208:<br>ONE OR MORE BIRTHS [ ] ↓ NO BIRTHS [ ]		→ 226

SECTION 2. REPRODUCTION

211 Now I would like to record the names of all your births, whether still alive or not, starting with the first one you had.
RECORD NAMES OF ALL THE BIRTHS IN 212. RECORD TWINS AND TRIPLETS ON SEPARATE ROWS. IF THERE ARE MORE THAN 10 BIRTHS, USE AN ADDITIONAL QUESTIONNAIRE, STARTING WITH THE SECOND ROW.

212	213	214	215	216	217	218	219	220	220B	221
What name was given to your (first/ next) baby?<br><br>RECORD NAME.<br><br>BIRTH HISTORY NUMBER.	Is (NAME) a boy or a girl?	Were any of these births twins?	On what day, month, and year was (NAME) born?	Is (NAME) still alive?	IF ALIVE:<br>How old was (NAME) at (NAME)'s last birthday?<br><br>RECORD AGE IN COMP-LETED YEARS.	IF ALIVE:<br>Is (NAME) living with you?	IF ALIVE:<br>RECORD HOUSEHOLD LINE NUMBER OF CHILD. RECORD '00' IF CHILD NOT LISTED IN HOUSEHOLD.	IF DEAD:<br>How old was (NAME) when (he/she) died?<br><br>IF '12 MONTHS' OR '1 YR', ASK: Did (NAME) have (his/her) first birthday?<br><br>THEN ASK: Exactly how many months old was (NAME) when (he/she) died?<br><br>RECORD DAYS IF LESS THAN 1 MONTH; MONTHS IF LESS THAN TWO YEARS; OR YEARS.	IF DEATH AT AGE 0-5<br>On what day, month and year did (NAME) die?	Were there any other live births between (NAME OF PREVIOUS BIRTH) and (NAME), including any children who died after birth?
01	BOY 1<br>GIRL 2	SING 1<br>MULT 2	DAY<br>MONTH<br>YEAR	YES 1<br>NO 2<br>(SKIP TO 220)	AGE IN YEARS	YES 1<br>NO 2	HOUSEHOLD LINE NUMBER<br>(NEXT BIRTH)	DAYS 1<br>MONTHS 2<br>YEARS 3	DAY<br>MONTH<br>YEAR	
02	BOY 1<br>GIRL 2	SING 1<br>MULT 2	DAY<br>MONTH<br>YEAR	YES 1<br>NO 2<br>(SKIP TO 220)	AGE IN YEARS	YES 1<br>NO 2	HOUSEHOLD LINE NUMBER<br>(SKIP TO 221)	DAYS 1<br>MONTHS 2<br>YEARS 3	DAY<br>MONTH<br>YEAR	YES 1<br>(ADD BIRTH)<br>NO 2<br>(NEXT BIRTH)
03	BOY 1<br>GIRL 2	SING 1<br>MULT 2	DAY<br>MONTH<br>YEAR	YES 1<br>NO 2<br>(SKIP TO 220)	AGE IN YEARS	YES 1<br>NO 2	HOUSEHOLD LINE NUMBER<br>(SKIP TO 221)	DAYS 1<br>MONTHS 2<br>YEARS 3	DAY<br>MONTH<br>YEAR	YES 1<br>(ADD BIRTH)<br>NO 2<br>(NEXT BIRTH)
04	BOY 1<br>GIRL 2	SING 1<br>MULT 2	DAY<br>MONTH<br>YEAR	YES 1<br>NO 2<br>(SKIP TO 220)	AGE IN YEARS	YES 1<br>NO 2	HOUSEHOLD LINE NUMBER<br>(SKIP TO 221)	DAYS 1<br>MONTHS 2<br>YEARS 3	DAY<br>MONTH<br>YEAR	YES 1<br>(ADD BIRTH)<br>NO 2<br>(NEXT BIRTH)
05	BOY 1<br>GIRL 2	SING 1<br>MULT 2	DAY<br>MONTH<br>YEAR	YES 1<br>NO 2<br>(SKIP TO 220)	AGE IN YEARS	YES 1<br>NO 2	HOUSEHOLD LINE NUMBER<br>(SKIP TO 221)	DAYS 1<br>MONTHS 2<br>YEARS 3	DAY<br>MONTH<br>YEAR	YES 1<br>(ADD BIRTH)<br>NO 2<br>(NEXT BIRTH)

212<br><br>What name was given to your (first/ next) baby?<br><br>RECORD NAME.<br><br>BIRTH HISTORY NUMBER.	213<br><br>Is (NAME) a boy or a girl?	214<br><br>Were any of these births twins?	215<br><br>On what day, month, and year was (NAME) born?	216<br><br>Is (NAME) still alive?	217<br>IF ALIVE:<br><br>How old was (NAME) at (NAME)'s last birthday?<br><br>RECORD AGE IN COMP-LETED YEARS.	218<br>IF ALIVE:<br><br>Is (NAME) living with you?	219<br>IF ALIVE:<br><br>RECORD HOUSEHOLD LINE NUMBER OF CHILD. RECORD '00' IF CHILD NOT LISTED IN HOUSEHOLD.	220<br>IF DEAD:<br><br>How old was (NAME) when (he/she) died?<br><br>IF '12 MONTHS' OR '1 YR', ASK: Did (NAME) have (his/her) first birthday?<br><br>THEN ASK: Exactly how many months old was (NAME) when (he/she) died?<br><br>RECORD DAYS IF LESS THAN 1 MONTH; MONTHS IF LESS THAN TWO YEARS; OR YEARS.	220B<br>**IF DEATH AT AGE 0-5**<br>On what day, month and year did (NAME) die?	221<br><br>Were there any other live births between (NAME OF PREVIOUS BIRTH) and (NAME), including any children who died after birth?
06	BOY 1<br>GIRL 2	SING 1<br>MULT 2	DAY ☐☐<br>MONTH ☐☐<br>☐☐☐☐<br>YEAR	YES 1<br>NO 2<br>(SKIP TO 220)	AGE IN YEARS<br>☐☐	YES 1<br>NO 2	HOUSEHOLD LINE NUMBER<br>☐☐<br>(SKIP TO 221)	DAYS 1 ☐☐<br>MONTHS 2 ☐☐<br>YEARS 3 ☐☐	DAY ☐☐<br>MONTH ☐☐<br>☐☐☐☐<br>YEAR	YES 1<br>(ADD BIRTH)<br>NO 2<br>(NEXT BIRTH)
07	BOY 1<br>GIRL 2	SING 1<br>MULT 2	DAY ☐☐<br>MONTH ☐☐<br>☐☐☐☐<br>YEAR	YES 1<br>NO 2<br>(SKIP TO 220)	AGE IN YEARS<br>☐☐	YES 1<br>NO 2	HOUSEHOLD LINE NUMBER<br>☐☐<br>(SKIP TO 221)	DAYS 1 ☐☐<br>MONTHS 2 ☐☐<br>YEARS 3 ☐☐	DAY ☐☐<br>MONTH ☐☐<br>☐☐☐☐<br>YEAR	YES 1<br>(ADD BIRTH)<br>NO 2<br>(NEXT BIRTH)
08	BOY 1<br>GIRL 2	SING 1<br>MULT 2	DAY ☐☐<br>MONTH ☐☐<br>☐☐☐☐<br>YEAR	YES 1<br>NO 2<br>(SKIP TO 220)	AGE IN YEARS<br>☐☐	YES 1<br>NO 2	HOUSEHOLD LINE NUMBER<br>☐☐<br>(SKIP TO 221)	DAYS 1 ☐☐<br>MONTHS 2 ☐☐<br>YEARS 3 ☐☐	DAY ☐☐<br>MONTH ☐☐<br>☐☐☐☐<br>YEAR	YES 1<br>(ADD BIRTH)<br>NO 2<br>(NEXT BIRTH)
09	BOY 1<br>GIRL 2	SING 1<br>MULT 2	DAY ☐☐<br>MONTH ☐☐<br>☐☐☐☐<br>YEAR	YES 1<br>NO 2<br>(SKIP TO 220)	AGE IN YEARS<br>☐☐	YES 1<br>NO 2	HOUSEHOLD LINE NUMBER<br>☐☐<br>(SKIP TO 221)	DAYS 1 ☐☐<br>MONTHS 2 ☐☐<br>YEARS 3 ☐☐	DAY ☐☐<br>MONTH ☐☐<br>☐☐☐☐<br>YEAR	YES 1<br>(ADD BIRTH)<br>NO 2<br>(NEXT BIRTH)
10	BOY 1<br>GIRL 2	SING 1<br>MULT 2	DAY ☐☐<br>MONTH ☐☐<br>☐☐☐☐<br>YEAR	YES 1<br>NO 2<br>(SKIP TO 220)	AGE IN YEARS<br>☐☐	YES 1<br>NO 2	HOUSEHOLD LINE NUMBER<br>☐☐<br>(SKIP TO 221)	DAYS 1 ☐☐<br>MONTHS 2 ☐☐<br>YEARS 3 ☐☐	DAY ☐☐<br>MONTH ☐☐<br>☐☐☐☐<br>YEAR	YES 1<br>(ADD BIRTH)<br>NO 2<br>(NEXT BIRTH)

SECTION 2. REPRODUCTION

NO.	QUESTIONS AND FILTERS	CODING CATEGORIES	SKIP
222	Have you had any live births since the birth of (NAME OF LAST BIRTH)?	YES . . . . . . 1 (RECORD BIRTH(S) IN TABLE)<br>NO 2	
223	COMPARE 208 WITH NUMBER OF BIRTHS IN BIRTH HISTORY<br>NUMBERS ARE SAME ☐<br>NUMBERS ARE DIFFERENT ☐ → (PROBE AND RECONCILE)		
223A	CHECK 220B: ENTER THE NUMBER OF DEATHS IN JANUARY 2014 OR LATER<br>IF NONE, RECORD `0'.	NUMBER OF DEATHS . . . . . . ☐	
224	CHECK 215: ENTER THE NUMBER OF BIRTHS IN 2013-2018	NUMBER OF BIRTHS . . . . . . ☐<br>NONE 0	0 → 226
225	**C** FOR EACH BIRTH IN 2013-2018, ENTER 'B' IN THE MONTH OF BIRTH IN THE CALENDAR. WRITE THE NAME OF THE CHILD TO THE LEFT OF THE 'B' CODE. FOR EACH BIRTH, ASK THE NUMBER OF COMPLETED MONTHS THE PREGNANCY LASTED AND RECORD 'P' IN EACH OF THE PRECEDING MONTHS ACCORDING TO THE DURATION OF PREGNANCY. (NOTE: THE NUMBER OF 'P's MUST BE ONE LESS THAN THE NUMBER OF MONTHS THAT THE PREGNANCY LASTED.)		
226	Are you pregnant now?	YES . . . . . . 1<br>NO . . . . . . 2<br>UNSURE 8	2, 8 → 230
227	How many months pregnant are you?<br>RECORD NUMBER OF COMPLETED MONTHS.<br>**C** ENTER 'P's IN THE CALENDAR, BEGINNING WITH THE MONTH OF INTERVIEW AND FOR THE TOTAL NUMBER OF COMPLETED MONTHS.	MONTHS . . . . . . ☐☐	
228	When you got pregnant, did you want to get pregnant at that time?	YES . . . . . . 1<br>NO 2	1 → 230
229	CHECK 208: TOTAL NUMBER OF BIRTHS<br>ONE OR MORE ☐ → a) Did you want to have a baby later on or did you not want any more children?<br>NONE ☐ → b) Did you want to have a baby later on or did you not want any children?	LATER . . . . . . 1<br>NO MORE/NONE 2	
230	Have you ever had a pregnancy that miscarried, was aborted, or ended in a stillbirth?	YES . . . . . . 1<br>NO 2	2 → 239
231	When did the last such pregnancy end?	MONTH . . . . . . ☐☐<br>YEAR . . . . . . ☐☐☐☐	

NO.	QUESTIONS AND FILTERS	CODING CATEGORIES	SKIP
232	CHECK 231: LAST PREGNANCY ENDED IN 2013-2018 □		→ 234
		LAST PREGNANCY ENDED IN 2012 OR EARLIER □	→ 239

LINE NO.	233<br>In what month and year did the preceding such pregnancy end?	234<br>How many months pregnant were you when that pregnancy ended?	235<br>Since January 2013, have you had any other pregnancies that did not result in a live birth?	
01		NUMBER OF MONTHS	YES . . . . 1<br>NO 2	→ NEXT LINE<br>→ 236
02	MONTH YEAR	NUMBER OF MONTHS	YES . . . . 1<br>NO 2	→ NEXT LINE<br>→ 236
03	MONTH YEAR	NUMBER OF MONTHS	YES . . . . 1<br>NO 2	→ NEXT LINE<br>→ 236
04	MONTH YEAR	NUMBER OF MONTHS	YES . . . . 1<br>NO 2	→ 236

NO.	QUESTIONS AND FILTERS	CODING CATEGORIES	SKIP
236	**C** FOR EACH PREGNANCY THAT DID NOT END IN A LIVE BIRTH IN 2013-2018 OR LATER, ENTER 'T' IN THE CALENDAR IN THE MONTH THAT THE PREGNANCY TERMINATED AND 'P' FOR THE REMAINING NUMBER OF COMPLETED MONTHS OF PREGNANCY.<br><br>IF THERE ARE MORE THAN FOUR PREGNANCIES THAT DID NOT END IN A LIVE BIRTH, USE AN ADDITIONAL QUESTIONNAIRE STARTING ON THE SECOND LINE.		
237	Did you have any miscarriages, abortions or stillbirths that ended before 2013?	YES . . . . 1<br>NO 2	 → 239
238	When did the last such pregnancy that terminated before 2013 end?	MONTH . . . .<br>YEAR	

SECTION 2. REPRODUCTION

NO.	QUESTIONS AND FILTERS	CODING CATEGORIES	SKIP
239	When did your last menstrual period start?<br><br>_______________<br>(DATE, IF GIVEN)	DAYS AGO . . . . 1 [ ][ ]<br>WEEKS AGO . . . . 2 [ ][ ]<br>MONTHS AGO . . . . 3 [ ][ ]<br>YEARS AGO . . . . 4 [ ][ ]<br><br>IN MENOPAUSE/<br>HAS HAD HYSTERECTOMY . . . . 994<br><br>BEFORE LAST BIRTH . . . . 995<br><br>NEVER MENSTRUATED 996	
240	From one menstrual period to the next, are there certain days when a woman is more likely to become pregnant?	YES . . . . 1<br>NO . . . . 2<br>DON'T KNOW 8	NO, DON'T KNOW → 242
241	Is this time just before her period begins, during her period, right after her period has ended, or halfway between two periods?	JUST BEFORE HER PERIOD BEGINS . . . . 1<br>DURING HER PERIOD . . . . 2<br>RIGHT AFTER HER PERIOD HAS ENDED . . . . 3<br>HALFWAY BETWEEN TWO PERIODS . . . . 4<br><br>OTHER _______________ 6<br>(SPECIFY)<br>DON'T KNOW 8	
242	After the birth of a child, can a woman become pregnant before her menstrual period has returned?	YES . . . . 1<br>NO . . . . 2<br>DON'T KNOW 8	

SECTION 3. CONTRACEPTION

301	Now I would like to talk about family planning - the various ways or methods that a couple can use to delay or avoid a pregnancy. Have you ever heard of (METHOD)?	
01	Female Sterilization.<br>PROBE: Women can have an operation to avoid having any more children.	YES . . . . . 1<br>NO 2
02	Male Sterilization.<br>PROBE: Men can have an operation to avoid having any more children.	YES . . . . . 1<br>NO 2
03	IUD.<br>PROBE: Women can have a loop or coil placed inside them by a doctor or a nurse which can prevent pregnancy for one or more years.	YES . . . . . 1<br>NO 2
04	Injectables.<br>PROBE: Women can have an injection by a health provider that stops them from becoming pregnant for one or more months.	YES . . . . . 1<br>NO 2
05	Implants.<br>PROBE: Women can have one or more small rods placed in their upper arm by a doctor or nurse which can prevent pregnancy for one or more years.	YES . . . . . 1<br>NO 2
06	Pill.<br>PROBE: Women can take a pill every day to avoid becoming pregnant.	YES . . . . . 1<br>NO 2
07	Condom.<br>PROBE: Men can put a rubber sheath on their penis before sexual intercourse.	YES . . . . . 1<br>NO 2
08	Female Condom.<br>PROBE: Women can place a sheath in their vagina before sexual intercourse.	YES . . . . . 1<br>NO 2
09	Emergency Contraception.<br>PROBE: As an emergency measure, within three days after they have unprotected sexual intercourse, women can take special pills to prevent pregnancy.	YES . . . . . 1<br>NO 2
10	Standard Days Method.<br>PROBE: A woman uses a string of colored beads to know the days she can get pregnant. On the days she can get pregnant, she uses a condom or does not have sexual intercourse.	YES . . . . . 1<br>NO 2
11	Lactational Amenorrhea Method (LAM).<br>PROBE: Up to six months after childbirth, before the menstrual period has returned, women use a method requiring frequent breastfeeding day and night.	YES . . . . . 1<br>NO 2
12	Rhythm Method.<br>PROBE: To avoid pregnancy, women do not have sexual intercourse on the days of the month they think they can get pregnant.	YES . . . . . 1<br>NO 2
13	Withdrawal.<br>PROBE: Men can be careful and pull out before climax.	YES . . . . . 1<br>NO 2
14	Have you heard of any other ways or methods that women or men can use to avoid pregnancy?	YES, MODERN METHOD<br>________ A<br>(SPECIFY)<br>YES, TRADITIONAL METHOD<br>________ B<br>(SPECIFY)<br>NO . . . . . Y

SECTION 3. CONTRACEPTION

NO.	QUESTIONS AND FILTERS	CODING CATEGORIES	SKIP
302	CHECK 226:<br>NOT PREGNANT OR UNSURE ☐	PREGNANT ☐	→ 312
303	Are you or your partner currently doing something or using any method to delay or avoid getting pregnant?	YES . . . . 1<br>NO 2	 → 312
304	Which method are you using?<br><br>RECORD ALL MENTIONED.<br><br>IF MORE THAN ONE METHOD MENTIONED, FOLLOW SKIP INSTRUCTION FOR HIGHEST METHOD IN LIST.	FEMALE STERILIZATION . . . . A<br>MALE STERILIZATION . . . . B<br>IUD . . . . C<br>INJECTABLES . . . . D<br>IMPLANTS . . . . E<br>PILL . . . . F<br>CONDOM . . . . G<br>FEMALE CONDOM . . . . H<br>EMERGENCY CONTRACEPTION . . . . I<br>STANDARD DAYS METHOD . . . . J<br>LACTATIONAL AMENORRHEA METHOD . . . . K<br>RHYTHM METHOD . . . . L<br>WITHDRAWAL . . . . M<br>OTHER MODERN METHOD . . . . X<br>OTHER TRADITIONAL METHOD . . . . Y	A–B → 307<br>C–E → 309<br>G → 306<br>H–Y → 309
305	What is the brand name of the pills you are using?<br><br>IF DON'T KNOW THE BRAND, ASK TO SEE THE PACKAGE.	DUOFEMCONFIDENCE . . . . 01<br>MICROGYNON . . . . 02<br>LOFEMENAL . . . . 03<br>NEOGYNON . . . . 04<br>OTHER ______ 96<br>(SPECIFY)<br>DON'T KNOW 98	→ 309
306	What is the brand name of the condoms you are using?<br><br>IF DON'T KNOW THE BRAND, ASK TO SEE THE PACKAGE.	MALE CONDOMS<br>GOLD CIRCLE . . . . 01<br>DUREX . . . . 02<br>ROUGH RIDER . . . . 03<br>TWIN LOTUS . . . . 04<br>PLAIN CONDOMS . . . . 05<br>GO FLEX . . . . 06<br>OTHER ______ 96<br>(SPECIFY)<br>DON'T KNOW 98	→ 309

SECTION 3. CONTRACEPTION

NO.	QUESTIONS AND FILTERS	CODING CATEGORIES	SKIP
307	In what facility did the sterilization take place?<br><br>PROBE TO IDENTIFY THE TYPE OF SOURCE.<br><br>IF UNABLE TO DETERMINE IF PUBLIC OR PRIVATE SECTOR, WRITE THE NAME OF THE PLACE.<br><br>________________<br>(NAME OF PLACE)	**PUBLIC SECTOR**<br>GOVERNMENT HOSPITAL . . . . 11<br>GOVERNMENT HEALTH CENTER . . . . 12<br>FAMILY PLANNING CLINIC . . . . 13<br>MOBILE CLINIC . . . . 14<br>OTHER PUBLIC SECTOR<br>________________ 16<br>(SPECIFY)<br><br>**PRIVATE MEDICAL SECTOR**<br>PRIVATE HOSPITAL/CLINIC . . . . 21<br>PRIVATE DOCTOR'S OFFICE . . . . 22<br>MOBILE CLINIC . . . . 23<br>NON-GOVERNMENT ORGANIZATION . . . . 24<br><br>OTHER PRIVATE MEDICAL SECTOR<br>________________ 26<br>(SPECIFY)<br><br>OTHER ________________ 96<br>(SPECIFY)<br>DON'T KNOW 98	
308	In what month and year was the sterilization performed?	MONTH . . . . [ ][ ]<br>YEAR . . . . [ ][ ][ ][ ]	→ 310
309	Since what month and year have you been using (CURRENT METHOD) without stopping?<br><br>PROBE: For how long have you been using (CURRENT METHOD) now without stopping?	MONTH . . . . [ ][ ]<br>YEAR . . . . [ ][ ][ ][ ]	
310	CHECK 308 AND 309, 215 AND 231: ANY BIRTH OR PREGNANCY TERMINATION AFTER MONTH AND YEAR OF START OF USE OF CONTRACEPTION IN 308 OR 309<br><br>NO [ ] ↓<br><br>YES [ ] → GO BACK TO 308 OR 309, PROBE AND RECORD MONTH AND YEAR AT START OF CONTINUOUS USE OF CURRENT METHOD (MUST BE AFTER LAST BIRTH OR PREGNANCY TERMINATION).		

311	CHECK 308 AND 309:<br>YEAR IS 2013-2018 ☐ — ENTER CODE FOR METHOD USED IN MONTH OF INTERVIEW IN THE CALENDAR AND IN EACH MONTH BACK TO THE DATE STARTED USING. THEN CONTINUE<br>YEAR IS 2012 OR EARLIER ☐ — ENTER CODE FOR METHOD USED IN MONTH OF INTERVIEW IN THE CALENDAR AND EACH MONTH BACK TO JANUARY 2013 . THEN (SKIP TO 324)
312	I would like to ask you some questions about the times you or your partner may have used a method to avoid getting pregnant during the last few years.<br>USE CALENDAR TO PROBE FOR EARLIER PERIODS OF USE AND NONUSE, STARTING WITH MOST RECENT USE, BACK TO JANUARY 2013. USE NAMES OF CHILDREN, DATES OF BIRTH, AND PERIODS OF PREGNANCY AS REFERENCE POINTS.

		COLUMN 1	COLUMN 2	COLUMN 3
312A	MONTH AND YEAR OF START OF INTERVAL OF USE OR NON-USE.	MONTH ☐☐<br>YEAR ☐☐☐☐	MONTH ☐☐<br>YEAR ☐☐☐☐	MONTH ☐☐<br>YEAR ☐☐☐☐
312B	Between (EVENT) in (MONTH/YEAR) and (EVENT) in (MONTH/YEAR), did you or your partner use any method of contraception?	YES . . . . 1<br>NO . . . . 2 (SKIP TO 312I)	YES . . . . 1<br>NO . . . . 2 (SKIP TO 312I)	YES . . . . 1<br>NO . . . . 2 (SKIP TO 312I)
312C	Which method was that?	METHOD CODE . . ☐	METHOD CODE . . ☐	METHOD CODE . . ☐
312D	How many months after (EVENT) in (MONTH/YEAR) did you start to use (METHOD)?<br>CIRCLE '95' IF RESPONDENT GIVES THE DATE OF STARTING TO USE THE METHOD.	IMMEDIATELY . . . . . 00<br>MONTHS . . ☐☐<br>(SKIP TO 312F)<br>DATE GIVEN 95	IMMEDIATELY . . . . . 00<br>MONTHS . . ☐☐<br>(SKIP TO 312F)<br>DATE GIVEN 95	IMMEDIATELY . . . . . 00<br>MONTHS . . ☐☐<br>(SKIP TO 312F)<br>DATE GIVEN 95
312E	RECORD MONTH AND YEAR RESPONDENT STARTED USING METHOD.	MONTH ☐☐<br>YEAR ☐☐☐☐	MONTH ☐☐<br>YEAR ☐☐☐☐	MONTH ☐☐<br>YEAR ☐☐☐☐
312F	For how many months did you use (METHOD)?<br>CIRCLE '95' IF RESPONDENT GIVES THE DATE OF TERMINATION OF USE.	MONTHS . . ☐☐<br>(SKIP TO 312H)<br>DATE GIVEN 95	MONTHS . . ☐☐<br>(SKIP TO 312H)<br>DATE GIVEN 95	MONTHS . . ☐☐<br>(SKIP TO 312H)<br>DATE GIVEN 95
312G	RECORD MONTH AND YEAR RESPONDENT STOPPED USING METHOD.	MONTH ☐☐<br>YEAR ☐☐☐☐	MONTH ☐☐<br>YEAR ☐☐☐☐	MONTH ☐☐<br>YEAR ☐☐☐☐
312H	Why did you stop using (METHOD)?	REASON STOPPED . . . . . ☐	REASON STOPPED . . . . . ☐	REASON STOPPED . . . . . ☐
312I		GO BACK TO 312A IN NEXT COLUMN; OR, IF NO MORE GAPS, GO TO 313.	GO BACK TO 312A IN NEXT COLUMN; OR, IF NO MORE GAPS, GO TO 313.	GO BACK TO 312A IN NEW QUESTIONNAIRE; OR, IF NO MORE GAPS, GO TO 313.

SECTION 3. CONTRACEPTION

NO.	QUESTIONS AND FILTERS	CODING CATEGORIES	SKIP
313	CHECK THE CALENDAR FOR USE OF ANY CONTRACEPTIVE METHOD IN ANY MONTH<br>NO METHOD USED [ ] ↓ ANY METHOD USED [ ]		ANY METHOD USED → 315
314	Have you ever used anything or tried in any way to delay or avoid getting pregnant?	YES . . . 1<br>NO . . . 2	1, 2 → 326
315	CHECK 304:<br><br>CIRCLE METHOD CODE:<br><br>IF MORE THAN ONE METHOD CODE CIRCLED IN 304, CIRCLE CODE FOR HIGHEST METHOD IN LIST.	NO CODE CIRCLED . . . 00<br>FEMALE STERILIZATION . . . 01<br>MALE STERILIZATION . . . 02<br>IUD . . . 03<br>INJECTABLES . . . 04<br>IMPLANTS . . . 05<br>PILL . . . 06<br>CONDOM . . . 07<br>FEMALE CONDOM . . . 08<br>EMERGENCY CONTRACEPTION . . . 09<br>STANDARD DAYS METHOD . . . 10<br>LACTATIONAL AMENORRHEA METHOD . . . 11<br>RHYTHM METHOD . . . 12<br>WITHDRAWAL . . . 13<br>OTHER MODERN METHOD . . . 95<br>OTHER TRADITIONAL METHOD . . . 96	00 → 326<br>01 → 319<br>02 → 327<br>11, 12, 13 → 323
316	You first started using (CURRENT METHOD) in (DATE FROM 309). Where did you get it at that time?<br><br>PROBE TO IDENTIFY THE TYPE OF SOURCE.<br><br>IF UNABLE TO DETERMINE IF PUBLIC OR PRIVATE SECTOR, WRITE THE NAME OF THE PLACE.<br><br>__________<br>(NAME OF PLACE)	**PUBLIC SECTOR**<br>GOVERNMENT HOSPITAL . . . 11<br>GOVERNMENT HEALTH CENTER . . . 12<br>FAMILY PLANNING CLINIC . . . 13<br>MOBILE CLINIC . . . 14<br>FIELDWORKER . . . 15<br>OTHER PUBLIC SECTOR<br>__________ 16<br>(SPECIFY)<br><br>**PRIVATE MEDICAL SECTOR**<br>PRIVATE HOSPITAL/CLINIC . . . 21<br>PHARMACY . . . 22<br>CHEMIST/PMS STORE . . . 23<br>PRIVATE DOCTOR . . . 24<br>MOBILE CLINIC . . . 25<br>FIELDWORKER . . . 26<br>OTHER PRIVATE MEDICAL SECTOR<br>__________ 27<br>(SPECIFY)<br><br>**OTHER SOURCE**<br>SHOP . . . 31<br>CHURCH . . . 32<br>FRIEND/RELATIVE . . . 33<br>NGO . . . 34<br><br>OTHER __________ 96<br>(SPECIFY)	
317	CHECK 304:<br><br>CIRCLE METHOD CODE:<br><br>IF MORE THAN ONE METHOD CODE CIRCLED IN 304, CIRCLE CODE FOR HIGHEST METHOD IN LIST.	IUD . . . 03<br>INJECTABLES . . . 04<br>IMPLANTS . . . 05<br>PILL . . . 06<br>CONDOM . . . 07<br>FEMALE CONDOM . . . 08<br>EMERGENCY CONTRACEPTION . . . 09<br>STANDARD DAYS METHOD . . . 10<br>OTHER MODERN METHOD . . . 95<br>OTHER TRADITIONAL METHOD . . . 96	07 → 323<br>08, 09, 10, 95 → 322<br>96 → 323

NO.	QUESTIONS AND FILTERS	CODING CATEGORIES	SKIP
318	At that time, were you told about side effects or problems you might have with the method?	YES . . . 1<br>NO . . . 2	1 → 321<br>2 → 320
319	When you got sterilized, were you told about side effects or problems you might have with the method?	YES . . . 1<br>NO . . . 2	1 → 321
320	Were you ever told by a health or family planning worker about side effects or problems you might have with the method?	YES . . . 1<br>NO . . . 2	2 → 322
321	Were you told what to do if you experienced side effects or problems?	YES . . . 1<br>NO . . . 2	
322	CHECK 318 AND 319:<br>ANY 'YES' ☐ ↓ / OTHER ☐ ↓<br>a) At that time, were you told about other methods of family planning that you could use?<br>b) When you obtained (CURRENT METHOD FROM 315) from (SOURCE OF METHOD FROM 307 OR 316), were you told about other methods of family planning that you could use?	YES . . . 1<br>NO . . . 2	1 → 324
323	Were you ever told by a health or family planning worker about other methods of family planning that you could use?	YES . . . 1<br>NO . . . 2	
324	CHECK 304:<br><br>CIRCLE METHOD CODE:<br><br>IF MORE THAN ONE METHOD CODE CIRCLED IN 304, CIRCLE CODE FOR HIGHEST METHOD IN LIST.	FEMALE STERILIZATION . . . 01<br>MALE STERILIZATION . . . 02<br>IUD . . . 03<br>INJECTABLES . . . 04<br>IMPLANTS . . . 05<br>PILL . . . 06<br>CONDOM . . . 07<br>FEMALE CONDOM . . . 08<br>EMERGENCY CONTRACEPTION . . . 09<br>STANDARD DAYS METHOD . . . 10<br>LACTATIONAL AMENORRHEA METHOD . . . 11<br>RHYTHM METHOD . . . 12<br>WITHDRAWAL . . . 13<br>OTHER MODERN METHOD . . . 95<br>OTHER TRADITIONAL METHOD . . . 96	01, 02 → 327<br>11, 12, 13 → 327<br>96 → 327

NO.	QUESTIONS AND FILTERS	CODING CATEGORIES	SKIP
325	Where did you obtain (CURRENT METHOD) the last time?<br><br>PROBE TO IDENTIFY THE TYPE OF SOURCE.<br><br>IF UNABLE TO DETERMINE IF PUBLIC OR PRIVATE SECTOR, WRITE THE NAME OF THE PLACE.<br><br>______________________<br>(NAME OF PLACE)	**PUBLIC SECTOR**<br>GOVERNMENT HOSPITAL . . . . . 11<br>GOVERNMENT HEALTH CENTER . . . . . 12<br>FAMILY PLANNING CLINIC . . . . . 13<br>MOBILE CLINIC . . . . . 14<br>FIELDWORKER . . . . . 15<br>OTHER PUBLIC SECTOR<br>______________________ 16<br>(SPECIFY)<br><br>**PRIVATE MEDICAL SECTOR**<br>PRIVATE HOSPITAL/CLINIC . . . . . 21<br>PHARMACY . . . . . 22<br>CHEMIST/PMS STORE . . . . . 23<br>PRIVATE DOCTOR . . . . . 24<br>MOBILE CLINIC . . . . . 25<br>FIELDWORKER . . . . . 26<br>OTHER PRIVATE MEDICAL SECTOR<br>______________________ 27<br>(SPECIFY)<br><br>**OTHER SOURCE**<br>SHOP . . . . . 31<br>CHURCH . . . . . 32<br>FRIEND/RELATIVE . . . . . 33<br>NGO . . . . . 34<br><br>OTHER ______________________ 96<br>(SPECIFY)	→ 327
326	Do you know of a place where you can obtain a method of family planning?	YES . . . . . 1<br>NO 2	
327	In the last 12 months, were you visited by a fieldworker?	YES . . . . . 1<br>NO 2	NO → 329
328	Did the fieldworker talk to you about family planning?	YES . . . . . 1<br>NO 2	
329	CHECK 202: CHILDREN LIVING WITH RESPONDENT<br>YES ☐ → a) In the last 12 months, have you visited a health facility for care for yourself or your children?<br>NO ☐ → b) In the last 12 months, have you visited a health facility for care for yourself?	YES . . . . . 1<br>NO 2	NO → 401
330	Did any staff member at the health facility speak to you about family planning methods?	YES . . . . . 1<br>NO 2	

SECTION 4. PREGNANCY AND POSTNATAL CARE

401	CHECK 224:<br>ONE OR MORE BIRTHS IN 2013-2018 ☐↓ NO BIRTHS IN 2013-2018 ☐ → 648		
402	CHECK 215. RECORD THE BIRTH HISTORY NUMBER IN 403 AND THE NAME AND SURVIVAL STATUS IN 404 FOR EACH BIRTH IN 2013-2018. ASK THE QUESTIONS ABOUT ALL OF THESE BIRTHS. BEGIN WITH THE LAST BIRTH. IF THERE ARE MORE THAN 2 BIRTHS, USE LAST COLUMN OF ADDITIONAL QUESTIONNAIRE(S).<br>Now I would like to ask some questions about your children born in the last five years. (We will talk about each separately.)		
403	BIRTH HISTORY NUMBER FROM 212 IN BIRTH HISTORY.	LAST BIRTH<br>BIRTH HISTORY NUMBER . . . . ☐☐	NEXT-TO-LAST BIRTH<br>BIRTH HISTORY NUMBER . . . . ☐☐
404	FROM 212 AND 216:	NAME ______<br>LIVING ☐↓ DEAD ☐↓	NAME ______<br>LIVING ☐↓ DEAD ☐↓
405	When you got pregnant with (NAME), did you want to get pregnant at that time?	YES . . . . 1 (SKIP TO 408)<br>NO 2	YES . . . . 1 (SKIP TO 426)<br>NO 2
406	CHECK 208:<br>ONLY ONE BIRTH ☐↓ MORE THAN ONE BIRTH ☐↓<br>a) Did you want to have a baby later on, or did you not want any children?<br>b) Did you want to have a baby later on, or did you not want any more children?	LATER . . . . 1<br>NO MORE/NONE . . . . 2 (SKIP TO 408)	LATER . . . . 1<br>NO MORE/NONE . . . . 2 (SKIP TO 426)
407	How much longer did you want to wait?	MONTHS . . . . 1 ☐☐<br>YEARS . . . . 2 ☐☐<br>DON'T KNOW 998	MONTHS . . . . 1 ☐☐<br>YEARS . . . . 2 ☐☐<br>DON'T KNOW 998
408	Did you see anyone for antenatal care for this pregnancy?	YES . . . . 1<br>NO . . . . 2 (SKIP TO 414)	
409	Whom did you see?<br>Anyone else?<br>PROBE TO IDENTIFY EACH TYPE OF PERSON AND RECORD ALL MENTIONED.	**HEALTH PERSONNEL**<br>DOCTOR . . . . A<br>NURSE/MIDWIFE . . . . B<br>AUXILIARY MIDWIFE . . . . . C<br>COMMUNITY EXTENSION HEALTH WORKER . . . . . D<br>**OTHER PERSON**<br>TRADITIONAL BIRTH ATTENDANT . . . . E<br>COMMUNITY/ VILLAGE HEALTH WORKER . . . . F<br>OTHER ______ X<br>(SPECIFY)	

SECTION 4. PREGNANCY AND POSTNATAL CARE

NO.	QUESTIONS AND FILTERS	LAST BIRTH<br>NAME ______	NEXT-TO-LAST BIRTH<br>NAME ______
410	Where did you receive antenatal care for this pregnancy?<br><br>Anywhere else?<br><br>PROBE TO IDENTIFY THE TYPE OF SOURCE.<br><br>IF UNABLE TO DETERMINE IF PUBLIC OR PRIVATE SECTOR, WRITE THE NAME OF THE PLACE.<br><br>______<br>(NAME OF PLACE)	**HOME**<br>HER HOME . . . . A<br>OTHER HOME . . . . B<br><br>**PUBLIC SECTOR**<br>GOVERNMENT HOSPITAL . . C<br>GOVERNMENT HEALTH CENTER . . . . D<br>GOVERNMENT HEALTH POST . . . . E<br>OTHER PUBLIC SECTOR<br>______ F<br>(SPECIFY)<br><br>**PRIVATE MEDICAL SECTOR**<br>PRIVATE HOSPITAL/ CLINIC . . . . G<br>OTHER PRIVATE MEDICAL SECTOR<br>______ H<br>(SPECIFY)<br><br>OTHER ______ X<br>(SPECIFY)	
411	How many months pregnant were you when you first received antenatal care for this pregnancy?	MONTHS . . . . [ ][ ]<br><br>DON'T KNOW 98	
412	How many times did you receive antenatal care during this pregnancy?	NUMBER OF TIMES . . . . . [ ][ ]<br><br>DON'T KNOW 98	
413	As part of your antenatal care during this pregnancy, were any of the following done at least once:<br><br>a) Was your blood pressure measured?<br>b) Did you give a urine sample?<br>c) Did you give a blood sample?	YES NO<br><br>a) BP . . . . 1 2<br>b) URINE . . . . 1 2<br>c) BLOOD 1 2	
414	During this pregnancy, were you given an injection in the arm to prevent the baby from getting tetanus, that is, convulsions after birth?	YES . . . . 1<br>NO . . . . 2<br>(SKIP TO 417)<br>DON'T KNOW 8	
415	During this pregnancy, how many times did you get a tetanus injection?	TIMES . . . . [ ]<br><br>DON'T KNOW 8	
416	CHECK 415:	2 OR MORE TIMES [ ] (SKIP TO 420)<br>OTHER [ ]	
417	At any time before this pregnancy, did you receive any tetanus injections?	YES . . . . 1<br>NO . . . . 2<br>(SKIP TO 420)<br>DON'T KNOW 8	

SECTION 4. PREGNANCY AND POSTNATAL CARE

NO.	QUESTIONS AND FILTERS	LAST BIRTH<br>NAME ______	NEXT-TO-LAST BIRTH<br>NAME ______
418	Before this pregnancy, how many times did you receive a tetanus injection?<br><br>IF 7 OR MORE TIMES, RECORD '7'.	TIMES . . . . [ ]<br><br>DON'T KNOW 8	
419	CHECK 418:<br>ONLY ONE [ ] ↓ | MORE THAN ONE TIME [ ] ↓<br>a) How many years ago did you receive that tetanus injection? | b) How many years ago did you receive the last tetanus injection prior to this pregnancy?	YEARS AGO . . . . . [ ][ ]	
420	During this pregnancy, were you given or did you buy any iron tablets or iron syrup?<br><br>SHOW TABLETS/SYRUP.	YES . . . . 1<br>NO . . . . 2<br>(SKIP TO 422) ←<br>DON'T KNOW 8	
421	During the whole pregnancy, for how many days did you take the tablets or syrup?<br><br>IF ANSWER IS NOT NUMERIC, PROBE FOR APPROXIMATE NUMBER OF DAYS.	DAYS . . . . . [ ][ ][ ]<br><br>DON'T KNOW 998	
422	During this pregnancy, did you take any drug for intestinal worms?	YES . . . . 1<br>NO . . . . 2<br>DON'T KNOW 8	
423	During this pregnancy, did you take SP/Fansidar to keep you from getting malaria?	YES . . . . 1<br>NO . . . . 2<br>(SKIP TO 426) ←<br>DON'T KNOW 8	
424	How many times did you take SP/Fansidar during this pregnancy?	TIMES . . . . [ ][ ]	
425	Did you get the SP/Fansidar during any antenatal care visit, during another visit to a health facility or from another source?<br><br>IF MORE THAN ONE SOURCE, RECORD THE HIGHEST SOURCE ON THE LIST.	ANTENATAL VISIT . . . . 1<br>ANOTHER FACILITY VISIT . . . . . 2<br>COMMUNITY HEALTH EXTENSION WORKER . . . . . 3<br>OTHER SOURCE 6	
426	When (NAME) was born, was (NAME) very large, larger than average, average, smaller than average, or very small?	VERY LARGE . . . . 1<br>LARGER THAN AVERAGE . . . . 2<br>AVERAGE . . . . 3<br>SMALLER THAN AVERAGE . . . . 4<br>VERY SMALL . . . . 5<br>DON'T KNOW 8	VERY LARGE . . . . 1<br>LARGER THAN AVERAGE . . . . 2<br>AVERAGE . . . . 3<br>SMALLER THAN AVERAGE . . . . 4<br>VERY SMALL . . . . 5<br>DON'T KNOW 8
427	Was (NAME) weighed at birth?	YES . . . . 1<br>NO . . . . 2<br>(SKIP TO 429) ←<br>DON'T KNOW 8	YES . . . . 1<br>NO . . . . 2<br>(SKIP TO 429) ←<br>DON'T KNOW 8

SECTION 4. PREGNANCY AND POSTNATAL CARE

NO.	QUESTIONS AND FILTERS	LAST BIRTH<br>NAME ______	NEXT-TO-LAST BIRTH<br>NAME ______
428	How much did (NAME) weigh?<br><br>RECORD WEIGHT IN KILOGRAMS FROM HEALTH CARD, IF AVAILABLE.	KG FROM CARD<br>1 [ ] . [ ][ ][ ]<br>KG FROM RECALL<br>2 [ ] . [ ][ ][ ]<br>DON'T KNOW 99998	KG FROM CARD<br>1 [ ] . [ ][ ][ ]<br>KG FROM RECALL<br>2 [ ] . [ ][ ][ ]<br>DON'T KNOW 99998
429	Who assisted with the delivery of (NAME)?<br><br>Anyone else?<br><br>PROBE FOR THE TYPE(S) OF PERSON(S) AND RECORD ALL MENTIONED.<br><br>IF RESPONDENT SAYS NO ONE ASSISTED, PROBE TO DETERMINE WHETHER ANY ADULTS WERE PRESENT AT THE DELIVERY.	**HEALTH PERSONNEL**<br>DOCTOR . . . . A<br>NURSE/MIDWIFE . . . . B<br>COMMUNITY HEALTH EXTENSION WORKER . . C<br>AUXILIARY MIDWIFE . . . . D<br>**OTHER PERSON**<br>TRADITIONAL BIRTH ATTENDANT . . . . E<br>RELATIVE/FRIEND . . . . F<br>OTHER ______ X<br>(SPECIFY)<br>NO ONE ASSISTED . . . . Y<br>(E–Y: SKIP TO 430)	**HEALTH PERSONNEL**<br>DOCTOR . . . . A<br>NURSE/MIDWIFE . . . . B<br>COMMUNITY HEALTH EXTENSION WORKER . . C<br>AUXILIARY MIDWIFE . . . . D<br>**OTHER PERSON**<br>TRADITIONAL BIRTH ATTENDANT . . . . E<br>RELATIVE/FRIEND . . . . F<br>OTHER ______ X<br>(SPECIFY)<br>NO ONE ASSISTED . . . . Y<br>(E–Y: SKIP TO 430)
429A	Immediately after delivery of (NAME) did you receive an injection in the thigh or buttock?	YES . . . . 1<br>NO . . . . 2<br>DON'T KNOW 8	YES . . . . 1<br>NO . . . . 2<br>DON'T KNOW 8
430	Where did you give birth to (NAME)?<br><br>PROBE TO IDENTIFY THE TYPE OF SOURCE.<br><br>IF UNABLE TO DETERMINE IF PUBLIC OR PRIVATE SECTOR, WRITE THE NAME OF THE PLACE.<br><br>______<br>(NAME OF PLACE)	**HOME**<br>HER HOME . . . . 11<br>OTHER HOME . . . . 12<br>(11–12: SKIP TO 434)<br><br>**PUBLIC SECTOR**<br>GOVERNMENT HOSPITAL . . 21<br>GOVERNMENT HEALTH CENTER . . . . 22<br>GOVERNMENT HEALTH POST . . . . 23<br>OTHER PUBLIC SECTOR<br>______ 26<br>(SPECIFY)<br><br>**PRIVATE MEDICAL SECTOR**<br>PRIVATE HOSPITAL/ CLINIC . . . . 31<br>OTHER PRIVATE MEDICAL SECTOR<br>______ 36<br>(SPECIFY)<br><br>OTHER ______ 96<br>(SPECIFY)<br>(SKIP TO 434)	**HOME**<br>HER HOME . . . . 11<br>OTHER HOME . . . . 12<br>(11–12: SKIP TO 459)<br><br>**PUBLIC SECTOR**<br>GOVERNMENT HOSPITAL . . 21<br>GOVERNMENT HEALTH CENTER . . . . 22<br>GOVERNMENT HEALTH POST . . . . 23<br>OTHER PUBLIC SECTOR<br>______ 26<br>(SPECIFY)<br><br>**PRIVATE MEDICAL SECTOR**<br>PRIVATE HOSPITAL/ CLINIC . . . . 31<br>OTHER PRIVATE MEDICAL SECTOR<br>______ 36<br>(SPECIFY)<br><br>OTHER ______ 96<br>(SPECIFY)<br>(SKIP TO 459)

SECTION 4. PREGNANCY AND POSTNATAL CARE

NO.	QUESTIONS AND FILTERS	LAST BIRTH<br>NAME ______	NEXT-TO-LAST BIRTH<br>NAME ______
430A	Did you move from another health facility to come to this facility or did you go directly from home to this facility, or from somewhere else that was not a health facility?	CAME FROM ANOTHER HEALTH FACILITY .... 1<br>CAME FROM HOME .... 2<br>CAME FROM OTHER NON-FACILITY LOCATION . . 3<br>DON'T KNOW .... 8<br>(SKIP TO 430F)	CAME FROM ANOTHER HEALTH FACILITY .... 1<br>CAME FROM HOME .... 2<br>CAME FROM OTHER NON-FACILITY LOCATION . . 3<br>DON'T KNOW .... 8<br>(SKIP TO 430F)
430B	Which health facility referred or send you to this facility where you gave birth to (NAME)?<br><br>PROBE TO IDENTIFY THE TYPE OF SOURCE.<br><br>IF UNABLE TO DETERMINE IF PUBLIC OR PRIVATE SECTOR, WRITE THE NAME OF THE PLACE.<br><br>______<br>(NAME OF PLACE)	**PUBLIC SECTOR**<br>GOVERNMENT HOSPITAL . . 21<br>GOVERNMENT HEALTH CENTER .... 22<br>GOVERNMENT HEALTH POST .... 23<br>OTHER PUBLIC SECTOR<br>______ 26<br>(SPECIFY)<br><br>**PRIVATE MEDICAL SECTOR**<br>PRIVATE HOSPITAL/ CLINIC .... 31<br>OTHER PRIVATE MEDICAL SECTOR<br>______ 36<br>(SPECIFY)<br><br>NO FORMAL REFERRAL ..... 41<br><br>OTHER ______ 96<br>(SPECIFY)	**PUBLIC SECTOR**<br>GOVERNMENT HOSPITAL . . 21<br>GOVERNMENT HEALTH CENTER .... 22<br>GOVERNMENT HEALTH POST .... 23<br>OTHER PUBLIC SECTOR<br>______ 26<br>(SPECIFY)<br><br>**PRIVATE MEDICAL SECTOR**<br>PRIVATE HOSPITAL/ CLINIC .... 31<br>OTHER PRIVATE MEDICAL SECTOR<br>______ 36<br>(SPECIFY)<br><br>NO FORMAL REFERRAL ..... 41<br><br>OTHER ______ 96<br>(SPECIFY)
430C	Why did you move from this facility to the facility where you gave birth to (NAME)?	PROBLEM DURING LABOR/ EMERGENCY .... 1<br>HEALTH PROFESSIONAL NOT AVAILABLE .... 2<br>FACILITY TOO CROWDED/ NO BED AVAILABLE ..... 3<br>FACILITY NOT OPEN .... 4<br><br>OTHER ______ 6<br>(SPECIFY)	PROBLEM DURING LABOR/ EMERGENCY .... 1<br>HEALTH PROFESSIONAL NOT AVAILABLE .... 2<br>FACILITY TOO CROWDED/ NO BED AVAILABLE ..... 3<br>FACILITY NOT OPEN .... 4<br><br>OTHER ______ 6<br>(SPECIFY)
430D	Did a health worker go with you when you moved to the facility where you gave birth to (NAME)?	YES .... 1<br>NO .... 2<br>DON'T KNOW 8	YES .... 1<br>NO .... 2<br>DON'T KNOW 8

NO.	QUESTIONS AND FILTERS	LAST BIRTH<br>NAME ______	NEXT-TO-LAST BIRTH<br>NAME ______
430E<br><br>430F	What means of transportation did you use to get from the facility that referred you to the faclity where you gave birth to (NAME)?<br><br>What means of transportation did you use to get to the health facility where you gave birth to (NAME)?<br><br>PROBE FOR THE TYPE (S) OF TRANSPORT USED AND RECORD ALL MENTIONED.	**MOTORISED**<br>AMBULANCE . . . . . A<br>PRIVATE CAR/TRUCK . . . . . B<br>TAXI/PAID DRIVER . . . . . C<br>TRICYCLE . . . . . D<br>MOTORCYCLE/SCOOTER . . E<br>BOAT WITH MOTOR . . . . . F<br>PUBLIC TRANSPORT/BUS . . G<br><br>**NOT MOTORISED**<br>BICYCLE . . . . . H<br>CANOE/BOAT WITHOUT MOTOR . . . . . I<br>ANIMAL-DRAWN CART . . . . . J<br>WALKING (ON FOOT) . . . . . K<br>CARRIED . . . . . L<br><br>OTHER ______ X<br>(SPECIFY)<br>DON'T KNOW . . . . . Z	**MOTORISED**<br>AMBULANCE . . . . . A<br>PRIVATE CAR/TRUCK . . . . . B<br>TAXI/PAID DRIVER . . . . . C<br>TRICYCLE . . . . . D<br>MOTORCYCLE/SCOOTER . . E<br>BOAT WITH MOTOR . . . . . F<br>PUBLIC TRANSPORT/BUS . . G<br><br>**NOT MOTORISED**<br>BICYCLE . . . . . H<br>CANOE/BOAT WITHOUT MOTOR . . . . . I<br>ANIMAL-DRAWN CART . . . . . J<br>WALKING (ON FOOT) . . . . . K<br>CARRIED . . . . . L<br><br>OTHER ______ X<br>(SPECIFY)<br>DON'T KNOW . . . . . Z
430G	How long did it take for you to decide to go and reach the health facility?<br><br>IF LESS THAN ONE HOUR, RECORD IN MINUTES.	MINUTES . . . . . 1 [ ][ ]<br>HOURS . . . . . 2 [ ][ ]<br><br>DON'T KNOW 998	MINUTES . . . . . 1 [ ][ ]<br>HOURS . . . . . 2 [ ][ ]<br><br>DON'T KNOW 998
431	How long after (NAME) was delivered did you stay there?<br><br>IF LESS THAN ONE DAY, RECORD HOURS;<br>IF LESS THAN ONE WEEK, RECORD DAYS.	HOURS . . . . . 1 [ ][ ]<br>DAYS . . . . . 2 [ ][ ]<br>WEEKS . . . . . 3 [ ][ ]<br><br>DON'T KNOW 998	
432	Was (NAME) delivered by caesarean, that is, did they cut your belly open to take the baby out?	YES . . . . . 1<br>NO . . . . . 2 (SKIP TO 434)	YES . . . . . 1<br>NO . . . . . 2 (SKIP TO 459)
433	When was the decision made to have the caesarean section? Was it before or after your labor pains started?	BEFORE . . . . . 1<br>AFTER 2	BEFORE . . . . . 1<br>AFTER 2
433A	What was the reason for taking the decision to have the caesarean section?	EXCESS BLEEDING . . . . . 1<br>BREECH POSITON . . . . . 2<br>MEDICAL CONDITION OF MOTHER . . . . . 3<br>CORD PROBLEM . . . . . 4<br>VOLUNTARY . . . . . 5<br><br>OTHER ______ 6<br>(SPECIFY)	EXCESS BLEEDING . . . . . 1<br>BREECH POSITON . . . . . 2<br>MEDICAL CONDITION OF MOTHER . . . . . 3<br>CORD PROBLEM . . . . . 4<br>VOLUNTARY . . . . . 5<br><br>OTHER ______ 6<br>(SPECIFY)
434	Immediately after the birth, was (NAME) put on your chest?	YES . . . . . 1<br>NO . . . . . 2 (SKIP TO 434B)<br>DON'T KNOW . . . . . 8 (SKIP TO 434B)	
434A	Was (NAME)'s bare skin touching your bare skin?	YES . . . . . 1<br>NO . . . . . 2<br>DON'T KNOW 8	

NO.	QUESTIONS AND FILTERS	LAST BIRTH<br>NAME ______	NEXT-TO-LAST BIRTH<br>NAME ______
434B	Was (NAME) wiped dry within a few minutes after birth?	YES . . . 1<br>NO . . . 2<br>DON'T KNOW . . . 8	
434C	How long after the birth was (NAME) bathed for the first time?<br><br>IF LESS THAN ONE HOUR, RECORD `00' HOURS;<br>IF LESS THAN 24 HOURS, RECORD HOURS; OTHERWISE, RECORD DAYS.	IMMEDIATELY . . . 000<br>HOURS . . . 1 [ ][ ]<br>DAYS . . . 2 [ ][ ]<br>DON'T KNOW . . . 998	
434D	CHECK 430: PLACE OF DELIVERY	CODE 11, 12, OR 96 CIRCLED [ ] ↓<br>OTHER [ ] (SKIP TO 434H)	
434E	What was used to cut the cord?	RAZOR BLADE . . . 1<br>KNIFE . . . 2<br>SCISSORS . . . 3<br>SICKLE . . . 4<br>OTHER ______ 6<br>(SPECIFY)<br>DON'T KNOW . . . 8	
434F	Was it new or had it ever been used before?	NEW . . . 1<br>USED BEFORE . . . 2<br>DON'T KNOW . . . 8	
434G	Was it boiled before it was used to cut the cord?	YES . . . 1<br>NO . . . 2<br>DON'T KNOW . . . 8	
434H	Was anything applied to the stump of the cord at any time?	YES . . . 1<br>NO . . . 2<br>(SKIP TO 434M) ←<br>DON'T KNOW . . . 8	
434I	What was applied?<br><br>Anything else?	CHLORHEXIDINE . . . A<br>OTHER ANTISEPTIC (ALCOHOL, SPIRIT, GENTIAN VIOLET, DETOL) . . . B<br>OLIVE OIL . . . C<br>ASH . . . D<br>ANIMAL DUNG . . . E<br>TURMERIC . . . F<br>OTHER ______ X<br>(SPECIFY)<br>DON'T KNOW . . . Z	
434J	CHECK 434I: SUBSTANCE APPLIED TO CORD	CODE `A' NOT CIRCLED [ ] ↓<br>CODE `A' CIRCLED [ ] (SKIP TO 434L)	
434K	Was chlorohexidine applied to the stump at any time?<br><br>SHOW SAMPLE OF CHLORHEXIDINE	YES . . . 1<br>NO . . . 2<br>(SKIP TO 434M) ←<br>DON'T KNOW . . . 8	

NO.	QUESTIONS AND FILTERS	LAST BIRTH<br>NAME ______	NEXT-TO-LAST BIRTH<br>NAME ______
434L	How long after the cord was cut was chlorhexidine first applied?<br><br>IF LESS THAN 1 HOUR, RECORD `00' HOURS; IF LESS THAN 24 HOURS, RECORD HOURS; OTHERWISE, RECORD DAYS.	HOURS . . . . 1 [ ][ ]<br>DAYS . . . . 2 [ ][ ]<br>DON'T KNOW 998	
434M	CHECK 430: PLACE OF DELIVERY	CODE 11, 12, OR 96 CIRCLED [ ] (SKIP TO 449)<br>OTHER [ ] ↓	
435	I would like to talk to you about checks on your health after delivery, for example, someone asking you questions about your health or examining you. Did anyone check on your health while you were still in the facility?	YES . . . . 1<br>NO . . . . 2<br>(SKIP TO 438)	
436	How long after delivery did the first check take place?<br><br>IF LESS THAN ONE DAY, RECORD HOURS;<br>IF LESS THAN ONE WEEK, RECORD DAYS.	HOURS . . . . 1 [ ][ ]<br>DAYS . . . . 2 [ ][ ]<br>WEEKS . . . . 3 [ ][ ]<br>DON'T KNOW 998	
437	Who checked on your health at that time?<br><br>PROBE FOR MOST QUALIFIED PERSON.	**HEALTH PERSONNEL**<br>DOCTOR . . . . 11<br>NURSE/MIDWIFE . . . . 12<br>COMMUNITY HEALTH EXTENSION WORKER . . 13<br>AUXILIARY MIDWIFE . . . . 14<br>**OTHER PERSON**<br>TRADITIONAL BIRTH ATTENDANT . . . . 21<br>COMMUNITY/ VILLAGE HEALTH WORKER . . . . 22<br>OTHER ______ 96<br>(SPECIFY)	
438	Now I would like to talk to you about checks on (NAME)'s health after delivery – for example, someone examining (NAME), checking the cord, or seeing if (NAME) is OK. Did anyone check on (NAME)'s health while you were still in the facility?	YES . . . . 1<br>NO . . . . 2<br>(SKIP TO 441)<br>DON'T KNOW 8	
439	How long after delivery was (NAME)'s health first checked?<br><br>IF LESS THAN ONE DAY, RECORD HOURS;<br>IF LESS THAN ONE WEEK, RECORD DAYS.	HOURS . . . . 1 [ ][ ]<br>DAYS . . . . 2 [ ][ ]<br>WEEKS . . . . 3 [ ][ ]<br>DON'T KNOW 998	

NO.	QUESTIONS AND FILTERS	LAST BIRTH<br>NAME ________	NEXT-TO-LAST BIRTH<br>NAME ________
440	Who checked on (NAME)'s health at that time?<br><br>PROBE FOR MOST QUALIFIED PERSON.	**HEALTH PERSONNEL**<br>DOCTOR . . . . 11<br>NURSE/MIDWIFE . . . . 12<br>COMMUNITY HEALTH EXTENSION WORKER . . 13<br>AUXILIARY MIDWIFE . . . . 14<br>**OTHER PERSON**<br>TRADITIONAL BIRTH ATTENDANT . . . . 21<br>COMMUNITY/ VILLAGE HEALTH WORKER . . . . 22<br><br>OTHER ________ 96<br>(SPECIFY)	
441	Now I want to talk to you about what happened after you left the facility. Did anyone check on your health after you left the facility?	YES . . . . 1<br>NO . . . . 2<br>(SKIP TO 445)	
442	How long after delivery did that check take place?<br><br>IF LESS THAN ONE DAY, RECORD HOURS;<br>IF LESS THAN ONE WEEK, RECORD DAYS.	HOURS . . . . 1 [ ][ ]<br>DAYS . . . . 2 [ ][ ]<br>WEEKS . . . . 3 [ ][ ]<br><br>DON'T KNOW 998	
443	Who checked on your health at that time?<br><br>PROBE FOR MOST QUALIFIED PERSON.	**HEALTH PERSONNEL**<br>DOCTOR . . . . 11<br>NURSE/MIDWIFE . . . . 12<br>COMMUNITY HEALTH EXTENSION WORKER . . 13<br>AUXILIARY MIDWIFE . . . . 14<br>**OTHER PERSON**<br>TRADITIONAL BIRTH ATTENDANT . . . . 21<br>COMMUNITY/ VILLAGE HEALTH WORKER . . . . 22<br><br>OTHER ________ 96<br>(SPECIFY)	

NO.	QUESTIONS AND FILTERS	LAST BIRTH<br>NAME ______	NEXT-TO-LAST BIRTH<br>NAME ______
444	Where did the check take place?<br><br>PROBE TO IDENTIFY THE TYPE OF SOURCE.<br><br>IF UNABLE TO DETERMINE IF PUBLIC OR PRIVATE SECTOR, WRITE THE NAME OF THE PLACE.<br><br>______<br>(NAME OF PLACE)	**HOME**<br>HER HOME . . . . 11<br>OTHER HOME . . . . 12<br><br>**PUBLIC SECTOR**<br>GOVERNMENT HOSPITAL . . 21<br>GOVERNMENT HEALTH CENTER . . . . 22<br>GOVERNMENT HEALTH POST . . . . 23<br>OTHER PUBLIC SECTOR<br>______ 26<br>(SPECIFY)<br><br>**PRIVATE MEDICAL SECTOR**<br>PRIVATE HOSPITAL/ CLINIC . . . . 31<br>OTHER PRIVATE MEDICAL SECTOR<br>______ 36<br>(SPECIFY)<br><br>OTHER ______ 96<br>(SPECIFY)	
445	I would like to talk to you about checks on (NAME)'s health after you left (FACILITY IN 430). Did any health care provider or a traditional birth attendant check on (NAME)'s health in the two months after you left (FACILITY IN 430)?	YES . . . . 1<br>NO . . . . 2<br>DON'T KNOW . . . . 8<br>(NO, DON'T KNOW: SKIP TO 457)	
446	How many hours, days or weeks after the birth of (NAME) did that check take place?<br><br>IF LESS THAN ONE DAY, RECORD HOURS;<br>IF LESS THAN ONE WEEK, RECORD DAYS.	HOURS . . . . 1 [ ][ ]<br>DAYS . . . . 2 [ ][ ]<br>WEEKS . . . . 3 [ ][ ]<br><br>DON'T KNOW 998	
447	Who checked on (NAME)'s health at that time?<br><br>PROBE FOR MOST QUALIFIED PERSON.	**HEALTH PERSONNEL**<br>DOCTOR . . . . 11<br>NURSE/MIDWIFE . . . . 12<br>COMMUNITY HEALTH EXTENSION WORKER . . 13<br>AUXILIARY MIDWIFE . . . . 14<br>**OTHER PERSON**<br>TRADITIONAL BIRTH ATTENDANT . . . . 21<br>COMMUNITY/ VILLAGE HEALTH WORKER . . . . 22<br><br>OTHER ______ 96<br>(SPECIFY)	

SECTION 4. PREGNANCY AND POSTNATAL CARE

NO.	QUESTIONS AND FILTERS	LAST BIRTH<br>NAME ______	NEXT-TO-LAST BIRTH<br>NAME ______
448	Where did this check of (NAME) take place?<br><br>PROBE TO IDENTIFY THE TYPE OF SOURCE.<br><br>IF UNABLE TO DETERMINE IF PUBLIC OR PRIVATE SECTOR, WRITE THE NAME OF THE PLACE.<br><br>______<br>(NAME OF PLACE)	**HOME**<br>HER HOME . . . . 11<br>OTHER HOME . . . . 12<br><br>**PUBLIC SECTOR**<br>GOVERNMENT HOSPITAL . . 21<br>GOVERNMENT HEALTH CENTER . . . . 22<br>GOVERNMENT HEALTH POST . . . . 23<br>OTHER PUBLIC SECTOR<br>______ 26<br>(SPECIFY)<br><br>**PRIVATE MEDICAL SECTOR**<br>PRIVATE HOSPITAL/ CLINIC . . . . 31<br>OTHER PRIVATE MEDICAL SECTOR<br>______ 36<br>(SPECIFY)<br><br>OTHER ______ 96<br>(SPECIFY)<br><br>(SKIP TO 457)	
449	I would like to talk to you about checks on your health after delivery, for example, someone asking you questions about your health or examining you. Did anyone check on your health after you gave birth to (NAME)?	YES . . . . 1<br>NO . . . . 2<br>(SKIP TO 453)	
450	How long after delivery did the first check take place?<br><br>IF LESS THAN ONE DAY, RECORD HOURS;<br>IF LESS THAN ONE WEEK, RECORD DAYS.	HOURS . . . . 1 [ ][ ]<br>DAYS . . . . 2 [ ][ ]<br>WEEKS . . . . 3 [ ][ ]<br><br>DON'T KNOW 998	
451	Who checked on your health at that time?<br><br>PROBE FOR MOST QUALIFIED PERSON.	**HEALTH PERSONNEL**<br>DOCTOR . . . . 11<br>NURSE/MIDWIFE . . . . 12<br>COMMUNITY HEALTH EXTENSION WORKER . . 13<br>AUXILIARY MIDWIFE . . . . 14<br>**OTHER PERSON**<br>TRADITIONAL BIRTH ATTENDANT . . . . 21<br>COMMUNITY/ VILLAGE HEALTH WORKER . . . . 22<br><br>OTHER ______ 96<br>(SPECIFY)	

NO.	QUESTIONS AND FILTERS	LAST BIRTH<br>NAME ______	NEXT-TO-LAST BIRTH<br>NAME ______
452	Where did this first check take place?<br><br>PROBE TO IDENTIFY THE TYPE OF SOURCE.<br><br>IF UNABLE TO DETERMINE IF PUBLIC OR PRIVATE SECTOR, WRITE THE NAME OF THE PLACE.<br><br>______<br>(NAME OF PLACE)	**HOME**<br>HER HOME . . . . 11<br>OTHER HOME . . . . 12<br><br>**PUBLIC SECTOR**<br>GOVERNMENT HOSPITAL . . 21<br>GOVERNMENT HEALTH CENTER . . . . 22<br>GOVERNMENT HEALTH POST . . . . 23<br>OTHER PUBLIC SECTOR<br>______ 26<br>(SPECIFY)<br><br>**PRIVATE MEDICAL SECTOR**<br>PRIVATE HOSPITAL/ CLINIC . . . . 31<br>OTHER PRIVATE MEDICAL SECTOR<br>______ 36<br>(SPECIFY)<br><br>OTHER ______ 96<br>(SPECIFY)	
453	I would like to talk to you about checks on (NAME)'s health after delivery – for example, someone examining (NAME), checking the cord, or seeing if (NAME) is OK. In the two months after (NAME) was born, did any health care provider or a traditional birth attendant check on (NAME)'s health?	YES . . . . 1<br>NO . . . . 2<br>(SKIP TO 457)<br>DON'T KNOW 8	
454	How many hours, days or weeks after the birth of (NAME) did the first check take place?<br><br>IF LESS THAN ONE DAY, RECORD HOURS;<br>IF LESS THAN ONE WEEK, RECORD DAYS.	HOURS AFTER BIRTH . . . . . 1 [ ][ ]<br>DAYS AFTER BIRTH . . . . . 2 [ ][ ]<br>WEEKS AFTER BIRTH . . . . . 3 [ ][ ]<br><br>DON'T KNOW 998	
455	Who checked on (NAME)'s health at that time?<br><br>PROBE FOR MOST QUALIFIED PERSON.	**HEALTH PERSONNEL**<br>DOCTOR . . . . 11<br>NURSE/MIDWIFE . . . . 12<br>COMMUNITY HEALTH EXTENSION WORKER . . 13<br>AUXILIARY MIDWIFE . . . . 14<br>**OTHER PERSON**<br>TRADITIONAL BIRTH ATTENDANT . . . . 21<br>COMMUNITY/ VILLAGE HEALTH WORKER . . . . 22<br><br>OTHER ______ 96<br>(SPECIFY)	

NO.	QUESTIONS AND FILTERS	LAST BIRTH<br>NAME ______	NEXT-TO-LAST BIRTH<br>NAME ______
456	Where did this first check of (NAME) take place?<br><br>PROBE TO IDENTIFY THE TYPE OF SOURCE.<br><br>IF UNABLE TO DETERMINE IF PUBLIC OR PRIVATE SECTOR, WRITE THE NAME OF THE PLACE.<br><br>______<br>(NAME OF PLACE)	**HOME**<br>HER HOME . . . . 11<br>OTHER HOME . . . . 12<br><br>**PUBLIC SECTOR**<br>GOVERNMENT HOSPITAL . . 21<br>GOVERNMENT HEALTH CENTER . . . . 22<br>GOVERNMENT HEALTH POST . . . . 23<br>OTHER PUBLIC SECTOR<br>______ 26<br>(SPECIFY)<br><br>**PRIVATE MEDICAL SECTOR**<br>PRIVATE HOSPITAL/ CLINIC . . . . 31<br>OTHER PRIVATE MEDICAL SECTOR<br>______ 36<br>(SPECIFY)<br><br>OTHER ______ 96<br>SPECIFY	
457	During the first two days after (NAME)'s birth, did any health care provider do the following:<br>a) Examine the cord?<br>b) Measure (NAME)'s temperature?<br>c) Counsel you on danger signs for newborns?<br>d) Counsel you on breastfeeding?<br>e) Observe (NAME) breastfeeding?	YES NO DK<br>a) CORD . . . . 1 2 8<br>b) TEMP. . . . . . 1 2 8<br>c) SIGNS . . . . . 1 2 8<br>d) COUNSEL BREAST-FEED 1 2 8<br>e) OBSERVE BREAST-FEED 1 2 8	
458	Has your menstrual period returned since the birth of (NAME)?	YES . . . . 1<br>(SKIP TO 460)<br>NO . . . . 2<br>(SKIP TO 461)	
459	Did your period return between the birth of (NAME) and your next pregnancy?		YES . . . . 1<br>NO . . . . 2<br>(SKIP TO 463)
460	For how many months after the birth of (NAME) did you not have a period?	MONTHS . . . . [ ][ ]<br><br>DON'T KNOW 98	MONTHS . . . . [ ][ ]<br><br>DON'T KNOW 98
461	CHECK 226: IS RESPONDENT PREGNANT?	NOT PREGNANT [ ]<br>PREGNANT OR UNSURE [ ] (SKIP TO 463)	
462	Have you had sexual intercourse since the birth of (NAME)?	YES . . . . 1<br>NO . . . . 2<br>(SKIP TO 464)	

NO.	QUESTIONS AND FILTERS	LAST BIRTH<br>NAME ________	NEXT-TO-LAST BIRTH<br>NAME ________
463	For how many months after the birth of (NAME) did you not have sexual intercourse?	MONTHS . . . . [ ][ ]<br>DON'T KNOW 98	MONTHS . . . . [ ][ ]<br>DON'T KNOW 98
464	Did you ever breastfeed (NAME)?	YES . . . . 1 (SKIP TO 466)<br>NO 2	YES . . . . 1<br>NO 2
465	CHECK 404: IS CHILD LIVING?	LIVING [ ] (SKIP TO 470)<br>DEAD [ ] (SKIP TO 471)	
466	How long after birth did you first put (NAME) to the breast?<br><br>IF LESS THAN 1 HOUR, RECORD '00' HOURS;<br>IF LESS THAN 24 HOURS, RECORD HOURS;<br>OTHERWISE, RECORD DAYS.	IMMEDIATELY . . . . 000<br><br>HOURS . . . . 1 [ ][ ]<br>DAYS . . . . 2 [ ][ ]	
467	In the first three days after delivery, was (NAME) given anything to drink other than breast milk?	YES . . . . 1<br>NO 2	
468	CHECK 404: IS CHILD LIVING?	LIVING [ ]<br>DEAD [ ] (SKIP TO 471)	LIVING [ ]<br>DEAD [ ] (SKIP TO 471)
469	Are you still breastfeeding (NAME)?	YES . . . . 1<br>NO 2	
470	Did (NAME) drink anything from a bottle with a nipple yesterday or last night?	YES . . . . 1<br>NO . . . . 2<br>DON'T KNOW 8	YES . . . . 1<br>NO . . . . 2<br>DON'T KNOW 8
471		GO BACK TO 405 IN NEXT COLUMN; OR, IF NO MORE BIRTHS, GO TO 501A.	GO BACK TO 405 IN NEXT-TO-LAST COLUMN OF NEW QUESTIONNAIRE; OR, IF NO MORE BIRTHS, GO TO 501A.

SECTION 5A. CHILD IMMUNIZATION (LAST BIRTH)

NO.	QUESTIONS AND FILTERS	CODING CATEGORIES	SKIP
501A	CHECK 215 IN THE BIRTH HISTORY: ANY BIRTHS IN 2015-2018?<br>ONE OR MORE BIRTHS IN 2015-2018 ☐ ↓	NO BIRTHS IN 2015-2018 ☐	→ 601
502A	RECORD THE NAME AND BIRTH HISTORY NUMBER FROM 212 OF THE LAST CHILD BORN IN 2015-2018.<br>NAME OF LAST BIRTH ________________	BIRTH HISTORY NUMBER . . . . ☐☐	
503A	CHECK 216 FOR CHILD:<br>LIVING ☐ ↓	DEAD ☐	→ 501B
504A	Do you have a card or other document where (NAME)'s vaccinations are written down?	YES, HAS ONLY A CARD . . . . 1<br>YES, HAS ONLY AN OTHER DOCUMENT . . . . . 2<br>YES, HAS CARD AND OTHER DOCUMENT . . . . . 3<br>NO, NO CARD AND NO OTHER DOCUMENT . . 4	1 → 507A<br><br>3 → 507A
505A	Did you ever have a vaccination card for (NAME)?	YES . . . . 1<br>NO 2	
506A	CHECK 504A:<br>CODE '2' CIRCLED ☐ ↓	CODE '4' CIRCLED ☐	→ 511A
507A	May I see the card or other document where (NAME)'s vaccinations are written down?	YES, ONLY CARD SEEN . . . . 1<br>YES, ONLY OTHER DOCUMENT SEEN . . . . 2<br>YES, CARD AND OTHER DOCUMENT SEEN . . 3<br>NO CARD AND NO OTHER DOCUMENT SEEN . . 4	4 → 511A

SECTION 5A. CHILD IMMUNIZATION (LAST BIRTH)

NO.	QUESTIONS AND FILTERS	CODING CATEGORIES	SKIP
	NAME OF LAST BIRTH ______________	BIRTH HISTORY NUMBER . . . . [ ][ ]	
508A	COPY DATES FROM THE CARD.<br>WRITE '44' IN 'DAY' COLUMN IF CARD SHOWS THAT A DOSE WAS GIVEN, BUT NO DATE IS RECORDED.		

	DAY	MONTH	YEAR
BCG			
HEPATITIS B AT BIRTH			
ORAL POLIO VACCINE (OPV) 0 (BIRTH DOSE)			
ORAL POLIO VACCINE (OPV) 1			
ORAL POLIO VACCINE (OPV) 2			
ORAL POLIO VACCINE (OPV) 3			
DPT-HEP.B-HIB (PENTAVALENT) 1			
DPT-HEP.B-HIB (PENTAVALENT) 2			
DPT-HEP.B-HIB (PENTAVALENT) 3			
PNEUMOCOCCAL 1			
PNEUMOCOCCAL 2			
PNEUMOCOCCAL 3			
INACTIVATED POLIO VIRUS (IPV)			
MEASLES 1			
MEASLES/MMR 2			
VITAMIN A (MOST RECENT)			

NO.	QUESTIONS AND FILTERS	CODING CATEGORIES	SKIP
509A	CHECK 508A: 'BCG' TO 'MEASLES/MMR 2' ALL RECORDED?<br>NO [ ] ↓	YES [ ]	→ 526A
510A	In addition to what is recorded on (this document/these documents), did (NAME) receive any other vaccinations, including vaccinations received in campaigns or immunization days or child health days?<br><br>RECORD 'YES' ONLY IF THE RESPONDENT MENTIONS AT LEAST ONE OF THE VACCINATIONS IN 508A THAT ARE NOT RECORDED AS HAVING BEEN GIVEN.	YES . . . . 1<br>(PROBE FOR VACCINATIONS AND WRITE '66' IN THE CORRESPONDING DAY COLUMN IN 508A THEN WRITE '00' IN THE CORRESPONDING DAY COLUMN FOR ALL VACCINATIONS NOT GIVEN)<br>(THEN SKIP TO 526A)<br><br>NO . . . . 2<br>DON'T KNOW . . . . 8<br>(WRITE '00' IN THE CORRESPONDING DAY COLUMN FOR ALL VACCINATIONS NOT GIVEN)<br>(THEN SKIP TO 526A)	

SECTION 5A. CHILD IMMUNIZATION (LAST BIRTH)

NO.	QUESTIONS AND FILTERS	CODING CATEGORIES	SKIP
	NAME OF LAST BIRTH ____________	BIRTH HISTORY NUMBER . . . . [ ][ ]	
511A	Did (NAME) ever receive any vaccinations to prevent (NAME) from getting diseases, including vaccinations received in campaigns or immunization days or child health days?	YES . . . 1<br>NO . . . 2<br>DON'T KNOW . . . 8	(NO, DON'T KNOW) → 526A
512A	Has (NAME) ever received a BCG vaccination against tuberculosis, that is, an injection in the arm or shoulder that usually causes a scar?	YES . . . 1<br>NO . . . 2<br>DON'T KNOW . . . 8	
513A	Within 24 hours after birth, did (NAME) receive a Hepatitis B vaccination, that is, an injection in the thigh to prevent Hepatitis B?	YES . . . 1<br>NO . . . 2<br>DON'T KNOW . . . 8	
514A	Has (NAME) ever received oral polio vaccine, that is, about two drops in the mouth to prevent polio?	YES . . . 1<br>NO . . . 2<br>DON'T KNOW . . . 8	(NO, DON'T KNOW) → 517A
515A	Did (NAME) receive the first oral polio vaccine in the first two weeks after birth or later?	FIRST TWO WEEKS . . . 1<br>LATER . . . 2	
516A	How many times did (NAME) receive the oral polio vaccine?	NUMBER OF TIMES . . . [ ]	
517A	Has (NAME) ever received a pentavalent vaccination, that is, an injection given in the thigh sometimes at the same time as polio drops?	YES . . . 1<br>NO . . . 2<br>DON'T KNOW . . . 8	(NO, DON'T KNOW) → 519A
518A	How many times did (NAME) receive the pentavalent vaccine?	NUMBER OF TIMES . . . [ ]	
519A	Has (NAME) ever received a pneumococcal vaccination, that is, an injection in the thigh to prevent pneumonia?	YES . . . 1<br>NO . . . 2<br>DON'T KNOW . . . 8	(NO, DON'T KNOW) → 521A
520A	How many times did (NAME) receive the pneumococcal vaccine?	NUMBER OF TIMES . . . [ ]	
521A	Has (NAME) ever received an inactivated polio vaccine (IPV), that is, an injection in the thigh to prevent polio?	YES . . . 1<br>NO . . . 2<br>DON'T KNOW . . . 8	
523A	Has (NAME) ever received a measles vaccination, that is, an injection in the arm to prevent measles?	YES . . . 1<br>NO . . . 2<br>DON'T KNOW . . . 8	(NO, DON'T KNOW) → 526A
524A	How many times did (NAME) receive the measles vaccine?	NUMBER OF TIMES . . . [ ]	
526A	CONTINUE WITH 501B.		

SECTION 5B. CHILD IMMUNIZATION (NEXT-TO-LAST BIRTH)

NO.	QUESTIONS AND FILTERS	CODING CATEGORIES	SKIP
501B	CHECK 215 IN THE BIRTH HISTORY: ANY MORE BIRTHS IN 2015-2018?<br>MORE BIRTHS IN 2015-2018 ☐ ↓ NO MORE BIRTHS IN 2015-2018 ☐		→ 601
502B	RECORD THE NAME AND BIRTH HISTORY NUMBER FROM 212 OF THE NEXT-TO-LAST CHILD BORN IN 2015-2018.<br>NAME OF NEXT-TO-LAST BIRTH ________	BIRTH HISTORY NUMBER . . . . ☐☐	
503B	CHECK 216 FOR CHILD:<br>LIVING ☐ ↓ DEAD ☐		→ 526B
504B	Do you have a card or other document where (NAME)'s vaccinations are written down?	YES, HAS ONLY A CARD . . . . 1<br>YES, HAS ONLY AN OTHER DOCUMENT . . . . . 2<br>YES, HAS CARD AND OTHER DOCUMENT . . . . . 3<br>NO, NO CARD AND NO OTHER DOCUMENT . . 4	1 → 507B<br><br>3 → 507B
505B	Did you ever have a vaccination card for (NAME)?	YES . . . . 1<br>NO 2	
506B	CHECK 504B:<br>CODE '2' CIRCLED ☐ ↓ CODE '4' CIRCLED ☐		→ 511B
507B	May I see the card or other document where (NAME)'s vaccinations are written down?	YES, ONLY CARD SEEN . . . . 1<br>YES, ONLY OTHER DOCUMENT SEEN . . . . 2<br>YES, CARD AND OTHER DOCUMENT SEEN . . 3<br>NO CARD AND NO OTHER DOCUMENT SEEN . . 4	 <br><br>4 → 511B

SECTION 5B. CHILD IMMUNIZATION (NEXT-TO-LAST BIRTH)

NO.	QUESTIONS AND FILTERS	CODING CATEGORIES	SKIP
	NAME OF NEXT-TO-LAST BIRTH ______________	BIRTH HISTORY NUMBER . . . . [ ][ ]	
508B	COPY DATES FROM THE CARD.<br>WRITE '44' IN 'DAY' COLUMN IF CARD SHOWS THAT A DOSE WAS GIVEN, BUT NO DATE IS RECORDED.		

	DAY	MONTH	YEAR
BCG			
HEPATITIS B AT BIRTH			
ORAL POLIO VACCINE (OPV) 0 (BIRTH DOSE)			
ORAL POLIO VACCINE (OPV) 1			
ORAL POLIO VACCINE (OPV) 2			
ORAL POLIO VACCINE (OPV) 3			
DPT-HEP.B-HIB (PENTAVALENT) 1			
DPT-HEP.B-HIB (PENTAVALENT) 2			
DPT-HEP.B-HIB (PENTAVALENT) 3			
PNEUMOCOCCAL 1			
PNEUMOCOCCAL 2			
PNEUMOCOCCAL 3			
INACTIVATED POLIO VIRUS (IPV)			
MEASLES 1			
MEASLES/MMR 2			
VITAMIN A (MOST RECENT)			

NO.	QUESTIONS AND FILTERS	CODING CATEGORIES	SKIP
509B	CHECK 508B: 'BCG' TO 'MEASLES/MMR 2' ALL RECORDED?<br>NO [ ] ↓	YES [ ]	→ 526B
510B	In addition to what is recorded on (this document/these documents), did (NAME) receive any other vaccinations, including vaccinations received in campaigns or immunization days or child health days?<br><br>RECORD 'YES' ONLY IF THE RESPONDENT MENTIONS AT LEAST ONE OF THE VACCINATIONS IN 508B THAT ARE NOT RECORDED AS HAVING BEEN GIVEN.	YES . . . . 1<br>(PROBE FOR VACCINATIONS AND WRITE '66' IN THE CORRESPONDING DAY COLUMN IN 508B THEN WRITE '00' IN THE CORRESPONDING DAY COLUMN FOR ALL VACCINATIONS NOT GIVEN)<br>(THEN SKIP TO 526B)<br><br>NO . . . . 2<br>DON'T KNOW . . . . 8<br>(WRITE '00' IN THE CORRESPONDING DAY COLUMN FOR ALL VACCINATIONS NOT GIVEN)<br>(THEN SKIP TO 526B)	

SECTION 5B. CHILD IMMUNIZATION (NEXT-TO-LAST BIRTH)

NO.	QUESTIONS AND FILTERS	CODING CATEGORIES	SKIP
	NAME OF NEXT-TO-LAST BIRTH ______________	BIRTH HISTORY NUMBER . . . . [ ][ ]	
511B	Did (NAME) ever receive any vaccinations to prevent (NAME) from getting diseases, including vaccinations received in campaigns or immunization days or child health days?	YES . . . . . 1<br>NO . . . . . 2<br>DON'T KNOW 8	2, 8 → 526B
512B	Has (NAME) ever received a BCG vaccination against tuberculosis, that is, an injection in the arm or shoulder that usually causes a scar?	YES . . . . . 1<br>NO . . . . . 2<br>DON'T KNOW 8	
513B	Within 24 hours after birth, did (NAME) receive a Hepatitis B vaccination, that is, an injection in the thigh to prevent Hepatitis B?	YES . . . . . 1<br>NO . . . . . 2<br>DON'T KNOW 8	
514B	Has (NAME) ever received oral polio vaccine, that is, about two drops in the mouth to prevent polio?	YES . . . . . 1<br>NO . . . . . 2<br>DON'T KNOW 8	2, 8 → 517B
515B	Did (NAME) receive the first oral polio vaccine in the first two weeks after birth or later?	FIRST TWO WEEKS . . . . . 1<br>LATER 2	
516B	How many times did (NAME) receive the oral polio vaccine?	NUMBER OF TIMES . . . . . [ ]	
517B	Has (NAME) ever received a pentavalent vaccination, that is, an injection given in the thigh sometimes at the same time as polio drops?	YES . . . . . 1<br>NO . . . . . 2<br>DON'T KNOW 8	2, 8 → 519B
518B	How many times did (NAME) receive the pentavalent vaccine?	NUMBER OF TIMES . . . . . [ ]	
519B	Has (NAME) ever received a pneumococcal vaccination, that is, an injection in the thigh to prevent pneumonia?	YES . . . . . 1<br>NO . . . . . 2<br>DON'T KNOW 8	2, 8 → 521B
520B	How many times did (NAME) receive the pneumococcal vaccine?	NUMBER OF TIMES . . . . . [ ]	
521B	Has (NAME) ever received an inactivated polio vaccine (IPV), that is, an injection in the thigh to prevent polio?	YES . . . . . 1<br>NO . . . . . 2<br>DON'T KNOW 8	
523B	Has (NAME) ever received a measles vaccination, that is, an injection in the arm to prevent measles?	YES . . . . . 1<br>NO . . . . . 2<br>DON'T KNOW 8	2, 8 → 526B
524B	How many times did (NAME) receive the measles vaccine?	NUMBER OF TIMES . . . . . [ ]	
526B	CHECK 215 IN BIRTH HISTORY: ANY MORE BIRTHS IN 2015-2018?<br>MORE BIRTHS IN 2015-2018 [ ] → (GO TO 502B IN AN ADDITIONAL QUESTIONNAIRE)<br>NO MORE BIRTHS IN 2015-2018 [ ]		NO MORE BIRTHS → 601

SECTION 6. CHILD HEALTH AND NUTRITION

601	CHECK 224:<br>ONE OR MORE BIRTHS IN 2013-2018 ☐ ↓<br>NO BIRTHS IN 2013-2018 ☐ → 648		
602	CHECK 215: RECORD THE BIRTH HISTORY NUMBER IN 603 AND THE NAME AND SURVIVAL STATUS IN 604 FOR EACH BIRTH IN 2013-2018. ASK THE QUESTIONS ABOUT ALL OF THESE BIRTHS. BEGIN WITH THE LAST BIRTH.<br>IF THERE ARE MORE THAN 2 BIRTHS, USE LAST COLUMN OF ADDITIONAL QUESTIONNAIRE(S).<br>Now I would like to ask some questions about your children born in the last five years. (We will talk about each separately.)		
603	BIRTH HISTORY NUMBER FROM 212 IN BIRTH HISTORY.	LAST BIRTH<br>BIRTH HISTORY NUMBER . . . . ☐☐	NEXT-TO-LAST BIRTH<br>BIRTH HISTORY NUMBER . . . . ☐☐
604	FROM 212 AND 216:	NAME ________<br>LIVING ☐ ↓<br>DEAD ☐ (SKIP TO 646)	NAME ________<br>LIVING ☐ ↓<br>DEAD ☐ (SKIP TO 646)
605	In the last six months, was (NAME) given a vitamin A dose like this?<br>SHOW COMMON TYPES OF CAPSULES.	YES . . . . . 1<br>NO . . . . . 2<br>DON'T KNOW 8	YES . . . . . 1<br>NO . . . . . 2<br>DON'T KNOW 8
606	In the last seven days, was (NAME) given iron pills, sprinkles with iron, or iron syrup like [this/any of these]?<br>SHOW COMMON TYPES OF PILLS/SPRINKLES/SYRUPS.	YES . . . . . 1<br>NO . . . . . 2<br>DON'T KNOW 8	YES . . . . . 1<br>NO . . . . . 2<br>DON'T KNOW 8
607	Was (NAME) given any medicine for deworming in the last six months?	YES . . . . . 1<br>NO . . . . . 2<br>DON'T KNOW 8	YES . . . . . 1<br>NO . . . . . 2<br>DON'T KNOW 8
608	Has (NAME) had diarrhea in the last 2 weeks?	YES . . . . . 1<br>NO . . . . . 2<br>(SKIP TO 618) ←<br>DON'T KNOW 8	YES . . . . . 1<br>NO . . . . . 2<br>(SKIP TO 618) ←<br>DON'T KNOW 8

NO.	QUESTIONS AND FILTERS	LAST BIRTH<br>NAME ______	NEXT-TO-LAST BIRTH<br>NAME ______
609	CHECK 469: CURRENTLY BREASTFEEDING?<br>YES ☐ / NO/ NOT ASKED ☐<br>a) Now I would like to know how much (NAME) was given to drink during the diarrhea including breastmilk. Was (NAME) given less than usual to drink, about the same amount, or more than usual to drink?<br>IF LESS, PROBE: Was (NAME) given much less than usual to drink or somewhat less?<br>b) Now I would like to know how much (NAME) was given to drink during the diarrhea. Was (NAME) given less than usual to drink, about the same amount, or more than usual to drink?<br>IF LESS, PROBE: Was (NAME) given much less than usual to drink or somewhat less?	MUCH LESS . . . . 1<br>SOMEWHAT LESS . . . . 2<br>ABOUT THE SAME . . . . 3<br>MORE . . . . 4<br>NOTHING TO DRINK . . . . 5<br>DON'T KNOW 8	MUCH LESS . . . . 1<br>SOMEWHAT LESS . . . . 2<br>ABOUT THE SAME . . . . 3<br>MORE . . . . 4<br>NOTHING TO DRINK . . . . 5<br>DON'T KNOW 8
610	When (NAME) had diarrhea, was (NAME) given less than usual to eat, about the same amount, more than usual, or nothing to eat?<br>IF LESS, PROBE: Was (NAME) given much less than usual to eat or somewhat less?	MUCH LESS . . . . 1<br>SOMEWHAT LESS . . . . 2<br>ABOUT THE SAME . . . . 3<br>MORE . . . . 4<br>STOPPED FOOD . . . . 5<br>NEVER GAVE FOOD . . . . 6<br>DON'T KNOW 8	MUCH LESS . . . . 1<br>SOMEWHAT LESS . . . . 2<br>ABOUT THE SAME . . . . 3<br>MORE . . . . 4<br>STOPPED FOOD . . . . 5<br>NEVER GAVE FOOD . . . . 6<br>DON'T KNOW 8
611	Did you seek advice or treatment for the diarrhea from any source?	YES . . . . 1<br>NO . . . . 2<br>(SKIP TO 615)	YES . . . . 1<br>NO . . . . 2<br>(SKIP TO 615)

NO.	QUESTIONS AND FILTERS	LAST BIRTH<br>NAME ______	NEXT-TO-LAST BIRTH<br>NAME ______
612	Where did you seek advice or treatment?<br><br>Anywhere else?<br><br>PROBE TO IDENTIFY THE TYPE OF SOURCE.<br><br>IF UNABLE TO DETERMINE IF PUBLIC OR PRIVATE SECTOR, WRITE THE NAME OF THE PLACE(S).<br><br>______<br>(NAME OF PLACE(S))	**PUBLIC SECTOR**<br>GOVERNMENT HOSPITAL . . A<br>GOVERNMENT HEALTH CENTER . . . . B<br>GOVERNMENT HEALTH POST . . . . C<br>MOBILE CLINIC . . . . D<br>FIELDWORKER . . . . E<br>OTHER PUBLIC SECTOR<br>______ F<br>(SPECIFY)<br><br>**PRIVATE MEDICAL SECTOR**<br>PRIVATE HOSPITAL/ CLINIC . . . . G<br>PHARMACY . . . . H<br>CHEMIST/PMS . . . . I<br>PRIVATE DOCTOR . . . . J<br>MOBILE CLINIC . . . . K<br>FIELDWORKER . . . . L<br>OTHER PRIVATE MEDICAL SECTOR<br>______ M<br>(SPECIFY)<br><br>**OTHER SOURCE**<br>SHOP . . . . N<br>TRADITIONAL PRACTITIONER . . . . O<br>MARKET . . . . P<br>ITINERANT DRUG SELLER . . . . Q<br>COMMUNITY-ORIENTED RESOURCE PERSON . . R<br><br>OTHER ______ X<br>(SPECIFY)	**PUBLIC SECTOR**<br>GOVERNMENT HOSPITAL . . A<br>GOVERNMENT HEALTH CENTER . . . . B<br>GOVERNMENT HEALTH POST . . . . C<br>MOBILE CLINIC . . . . D<br>FIELDWORKER . . . . E<br>OTHER PUBLIC SECTOR<br>______ F<br>(SPECIFY)<br><br>**PRIVATE MEDICAL SECTOR**<br>PRIVATE HOSPITAL/ CLINIC . . . . G<br>PHARMACY . . . . H<br>CHEMIST/PMS . . . . I<br>PRIVATE DOCTOR . . . . J<br>MOBILE CLINIC . . . . K<br>FIELDWORKER . . . . L<br>OTHER PRIVATE MEDICAL SECTOR<br>______ M<br>(SPECIFY)<br><br>**OTHER SOURCE**<br>SHOP . . . . N<br>TRADITIONAL PRACTITIONER . . . . O<br>MARKET . . . . P<br>ITINERANT DRUG SELLER . . . . Q<br>COMMUNITY-ORIENTED RESOURCE PERSON . . R<br><br>OTHER ______ X<br>(SPECIFY)
613	CHECK 612:	TWO OR MORE CODES CIRCLED ☐<br>ONLY ONE CODE CIRCLED ☐ (SKIP TO 615)	TWO OR MORE CODES CIRCLED ☐<br>ONLY ONE CODE CIRCLED ☐ (SKIP TO 615)
614	Where did you first seek advice or treatment?<br><br>USE LETTER CODE FROM 612.	FIRST PLACE . . . . ☐	FIRST PLACE . . . . ☐

SECTION 6. CHILD HEALTH AND NUTRITION

NO.	QUESTIONS AND FILTERS	LAST BIRTH<br>NAME ________	NEXT-TO-LAST BIRTH<br>NAME ________
615	Was (NAME) given any of the following at any time since (NAME) started having the diarrhea:<br>a) A fluid made from a special packet called CHI ORS, Emzorlyte, Orasure, Olpharm ORS etc.?<br>c) A government-recommended homemade fluid?<br>d) Zinc tablets or syrup?	YES NO DK<br>a) FLUID FROM ORS PACKET . . 1 2 8<br>c) HOMEMADE FLUID . . . . . 1 2 8<br>d) ZINC 1 2 8	YES NO DK<br>a) FLUID FROM ORS PACKET . . 1 2 8<br>c) HOMEMADE FLUID . . . . . 1 2 8<br>d) ZINC 1 2 8
616	CHECK 615:<br>ANY 'YES' ☐ / ALL 'NO' OR 'DK' ☐<br>a) Was anything else given to treat the diarrhea?<br>b) Was anything given to treat the diarrhea?	YES . . . . 1<br>NO . . . . 2 (SKIP TO 618)<br>DON'T KNOW . . . . . . 8 (SKIP TO 618)	YES . . . . 1<br>NO . . . . 2 (SKIP TO 618)<br>DON'T KNOW . . . . . . 8 (SKIP TO 618)
617	CHECK 615:<br>ANY 'YES' ☐ / ALL 'NO' OR 'DK' ☐<br>a) What else was given to treat the diarrhea? Anything else?<br>b) What was given to treat the diarrhea? Anything else?<br>RECORD ALL TREATMENTS GIVEN.	**PILL OR SYRUP**<br>ANTIBIOTIC . . . . . A<br>ANTIMOTILITY . . . . . B<br>OTHER (NOT ANTIBIOTIC OR ANTIMOTILITY) . . . . . C<br>UNKNOWN PILL OR SYRUP . . . . . D<br>**INJECTION**<br>ANTIBIOTIC . . . . . E<br>NON-ANTIBIOTIC . . . . . F<br>UNKNOWN INJECTION . . . . . G<br>(IV) INTRAVENOUS . . . . . H<br>HOME REMEDY/ HERBAL MEDICINE . . . . . I<br>OTHER ________ X<br>(SPECIFY)	**PILL OR SYRUP**<br>ANTIBIOTIC . . . . . A<br>ANTIMOTILITY . . . . . B<br>OTHER (NOT ANTIBIOTIC OR ANTIMOTILITY) . . . . . C<br>UNKNOWN PILL OR SYRUP . . . . . D<br>**INJECTION**<br>ANTIBIOTIC . . . . . E<br>NON-ANTIBIOTIC . . . . . F<br>UNKNOWN INJECTION . . . . . G<br>(IV) INTRAVENOUS . . . . . H<br>HOME REMEDY/ HERBAL MEDICINE . . . . . I<br>OTHER ________ X<br>(SPECIFY)
618	Has (NAME) been ill with a fever at any time in the last 2 weeks?	YES . . . . 1<br>NO . . . . 2 (SKIP TO 620)<br>DON'T KNOW . . . . . . 8 (SKIP TO 620)	YES . . . . 1<br>NO . . . . 2 (SKIP TO 620)<br>DON'T KNOW . . . . . . 8 (SKIP TO 620)
619	At any time during the illness, did (NAME) have blood taken from (NAME)'s finger or heel for testing?	YES . . . . 1<br>NO . . . . 2<br>DON'T KNOW 8	YES . . . . 1<br>NO . . . . 2<br>DON'T KNOW 8
620	Has (NAME) had an illness with a cough at any time in the last 2 weeks?	YES . . . . 1<br>NO . . . . 2<br>DON'T KNOW 8	YES . . . . 1<br>NO . . . . 2<br>DON'T KNOW 8
621	Has (NAME) had fast, short, rapid breaths or difficulty breathing at any time in the last 2 weeks?	YES . . . . 1<br>NO . . . . 2 (SKIP TO 623)<br>DON'T KNOW . . . . . . 8 (SKIP TO 623)	YES . . . . 1<br>NO . . . . 2 (SKIP TO 623)<br>DON'T KNOW . . . . . . 8 (SKIP TO 623)

NO.	QUESTIONS AND FILTERS	LAST BIRTH<br>NAME ______	NEXT-TO-LAST BIRTH<br>NAME ______
622	Was the fast or difficult breathing due to a problem in the chest or to a blocked or runny nose?	CHEST ONLY . . . . 1<br>NOSE ONLY . . . . 2<br>BOTH . . . . 3<br>OTHER ______ 6<br>(SPECIFY)<br>DON'T KNOW . . . . 8<br>(SKIP TO 624)	CHEST ONLY . . . . 1<br>NOSE ONLY . . . . 2<br>BOTH . . . . 3<br>OTHER ______ 6<br>(SPECIFY)<br>DON'T KNOW . . . . 8<br>(SKIP TO 624)
623	CHECK 618: HAD FEVER?	YES ☐ NO OR DK ☐<br>(SKIP TO 646)	YES ☐ NO OR DK ☐<br>(SKIP TO 646)
624	Did you seek advice or treatment for the illness from any source?	YES . . . . 1<br>NO . . . . 2<br>(SKIP TO 629)	YES . . . . 1<br>NO . . . . 2<br>(SKIP TO 629)
625	Where did you seek advice or treatment?<br><br>Anywhere else?<br><br>PROBE TO IDENTIFY THE TYPE OF SOURCE.<br><br>IF UNABLE TO DETERMINE IF PUBLIC OR PRIVATE SECTOR, WRITE THE NAME OF THE PLACE(S).<br><br>______<br>(NAME OF PLACE(S))	**PUBLIC SECTOR**<br>GOVERNMENT HOSPITAL . . A<br>GOVERNMENT HEALTH CENTER . . . . B<br>GOVERNMENT HEALTH POST . . . . C<br>MOBILE CLINIC . . . . D<br>FIELDWORKER/CHW . . . . . E<br>OTHER PUBLIC SECTOR ______ F<br>(SPECIFY)<br><br>**PRIVATE MEDICAL SECTOR**<br>PRIVATE HOSPITAL/ CLINIC . . . . G<br>PHARMACY . . . . H<br>CHEMIST/PMS . . . . I<br>PRIVATE DOCTOR . . . . J<br>MOBILE CLINIC . . . . K<br>FIELDWORKER/CHW . . . . . L<br>OTHER PRIVATE MEDICAL SECTOR ______ M<br>(SPECIFY)<br><br>**OTHER SOURCE**<br>SHOP . . . . N<br>TRADITIONAL PRACTITIONER . . . . O<br>MARKET . . . . P<br>ITINERANT DRUG SELLER . . . . Q<br>COMMUNITY-ORIENTED RESOURCE PERSON . . R<br><br>OTHER ______ X<br>(SPECIFY)	**PUBLIC SECTOR**<br>GOVERNMENT HOSPITAL . . A<br>GOVERNMENT HEALTH CENTER . . . . B<br>GOVERNMENT HEALTH POST . . . . C<br>MOBILE CLINIC . . . . D<br>FIELDWORKER/CHW . . . . . E<br>OTHER PUBLIC SECTOR ______ F<br>(SPECIFY)<br><br>**PRIVATE MEDICAL SECTOR**<br>PRIVATE HOSPITAL/ CLINIC . . . . G<br>PHARMACY . . . . H<br>CHEMIST/PMS . . . . I<br>PRIVATE DOCTOR . . . . J<br>MOBILE CLINIC . . . . K<br>FIELDWORKER/CHW . . . . . L<br>OTHER PRIVATE MEDICAL SECTOR ______ M<br>(SPECIFY)<br><br>**OTHER SOURCE**<br>SHOP . . . . N<br>TRADITIONAL PRACTITIONER . . . . O<br>MARKET . . . . P<br>ITINERANT DRUG SELLER . . . . Q<br>COMMUNITY-ORIENTED RESOURCE PERSON . . R<br><br>OTHER ______ X<br>(SPECIFY)
626	CHECK 625:	TWO OR MORE CODES CIRCLED ☐ ONLY ONE CODE CIRCLED ☐<br>(SKIP TO 628)	TWO OR MORE CODES CIRCLED ☐ ONLY ONE CODE CIRCLED ☐<br>(SKIP TO 628)

NO.	QUESTIONS AND FILTERS	LAST BIRTH<br>NAME ______	NEXT-TO-LAST BIRTH<br>NAME ______
627	Where did you first seek advice or treatment?<br><br>USE LETTER CODE FROM 625.	FIRST PLACE . . . . [ ]	FIRST PLACE . . . . [ ]
628	How many days after the illness began did you first seek advice or treatment for (NAME)?<br>IF THE SAME DAY RECORD '00'.	DAYS . . . . [ ][ ]	DAYS . . . . [ ][ ]
629	At any time during the illness, did (NAME) take any drugs for the illness?	YES . . . . 1<br>NO . . . . 2<br>(SKIP TO 646)<br>DON'T KNOW 8	YES . . . . 1<br>NO . . . . 2<br>(SKIP TO 646)<br>DON'T KNOW 8
630	What drugs did (NAME) take?<br><br>Any other drugs?<br><br>RECORD ALL MENTIONED.	**ANTIMALARIAL DRUGS**<br>ARTEMISININ<br>COMBINATION<br>THERAPY (ACT) . . . . . A<br>SP/FANSIDAR . . . . B<br>CHLOROQUINE . . . . C<br>AMODIAQUINE . . . . D<br>QUININE<br>PILLS . . . . E<br>INJECTION/IV . . . . F<br>ARTESUNATE<br>RECTAL . . . . G<br>INJECTION/IV . . . . H<br><br>OTHER ANTIMALARIAL<br>______ I<br>(SPECIFY)<br><br>**ANTIBIOTIC DRUGS**<br>PILL/SYRUP . . . . J<br>INJECTION/IV . . . . K<br><br>**OTHER DRUGS**<br>ASPIRIN . . . . L<br>PARACETAMOL . . . . M<br>IBUPROFEN . . . . N<br><br>OTHER ______ X<br>(SPECIFY)<br>DON'T KNOW . . . . Z	**ANTIMALARIAL DRUGS**<br>ARTEMISININ<br>COMBINATION<br>THERAPY (ACT) . . . . . A<br>SP/FANSIDAR . . . . B<br>CHLOROQUINE . . . . C<br>AMODIAQUINE . . . . D<br>QUININE<br>PILLS . . . . E<br>INJECTION/IV . . . . F<br>ARTESUNATE<br>RECTAL . . . . G<br>INJECTION/IV . . . . H<br><br>OTHER ANTIMALARIAL<br>______ I<br>(SPECIFY)<br><br>**ANTIBIOTIC DRUGS**<br>PILL/SYRUP . . . . J<br>INJECTION/IV . . . . K<br><br>**OTHER DRUGS**<br>ASPIRIN . . . . L<br>PARACETAMOL . . . . M<br>IBUPROFEN . . . . N<br><br>OTHER ______ X<br>(SPECIFY)<br>DON'T KNOW . . . . Z
631	CHECK 630:<br>ANY CODE A-I CIRCLED?	YES [ ] NO [ ]<br>(SKIP TO 646)	YES [ ] NO [ ]<br>(SKIP TO 646)

SECTION 6. CHILD HEALTH AND NUTRITION

NO.	QUESTIONS AND FILTERS	LAST BIRTH<br>NAME ______	NEXT-TO-LAST BIRTH<br>NAME ______
632	CHECK 630:<br>ARTEMISININ COMBINATION THERAPY ('A') GIVEN	CODE 'A' CIRCLED ☐<br>CODE 'A' NOT CIRCLED ☐ (SKIP TO 646)	CODE 'A' CIRCLED ☐<br>CODE 'A' NOT CIRCLED ☐ (SKIP TO 646)
633	How long after the fever started did (NAME) first take an artemisinin combination therapy?	SAME DAY . . . . 0<br>NEXT DAY . . . . 1<br>TWO DAYS AFTER FEVER . . . . 2<br>THREE OR MORE DAYS AFTER FEVER . . . . 3<br>DON'T KNOW 8	SAME DAY . . . . 0<br>NEXT DAY . . . . 1<br>TWO DAYS AFTER FEVER . . . . 2<br>THREE OR MORE DAYS AFTER FEVER . . . . 3<br>DON'T KNOW 8
646		GO BACK TO 604 IN NEXT COLUMN; OR, IF NO MORE BIRTHS, GO TO 647.	GO TO 604 IN NEXT-TO-LAST COLUMN OF NEW QUESTIONNAIRE; OR, IF NO MORE BIRTHS, GO TO 647.

NO.	QUESTIONS AND FILTERS	CODING CATEGORIES	SKIP
647	CHECK 615(a) AND 615(b), ALL COLUMNS:<br>NO CHILD RECEIVED FLUID FROM ORS PACKET ☐ ↓	ANY CHILD RECEIVED FLUID FROM ORS PACKET ☐	→ 649
648	Have you ever heard of a special product ORS called CHI ORS, Emzorlyte, Orasure, Olpharm ORS etc. you can get for the treatment of diarrhea?	YES . . . . 1<br>NO 2	
649	CHECK 215 AND 218, ALL ROWS: NUMBER OF CHILDREN BORN IN 2016-2018 LIVING WITH THE RESPONDENT<br>ONE OR MORE ☐ ↓<br>________________<br>(NAME OF YOUNGEST CHILD LIVING WITH HER)<br>↓	NONE ☐	→ 653A

SECTION 6. CHILD HEALTH AND NUTRITION

NO.	QUESTIONS AND FILTERS	CODING CATEGORIES				SKIP
650	Now I would like to ask you about liquids or foods that (NAME FROM 649) had yesterday during the day or at night. I am interested in whether your child had the item I mention even if it was combined with other foods.<br>Did (NAME FROM 649) drink or eat:		YES	NO	DK	
	a) Plain water?	a)	1	2	8	
	b) Juice or juice drinks?	b)	1	2	8	
	c) Clear broth?	c)	1	2	8	
	d) Milk such as tinned, powdered, or fresh animal milk?<br>IF YES: How many times did (NAME) drink milk?<br>IF 7 OR MORE TIMES, RECORD '7'.	d) . . . .<br>NUMBER OF TIMES DRANK	1	2	8	
	e) Infant formula (Nan, SMA Gold, My Boy, Friso, Lactogen, Peak Milk 123, Cow and Gate, etc.)?<br>IF YES: How many times did (NAME) drink infant formula?<br>IF 7 OR MORE TIMES, RECORD '7'.	e) . . . .<br>NUMBER OF TIMES DRANK	1	2	8	
	f) Any other liquids?	f)	1	2	8	
	g) Yogurt?<br>IF YES: How many times did (NAME) eat yogurt?<br>IF 7 OR MORE TIMES, RECORD '7'.	g) . . . .<br>NUMBER OF TIMES ATE	1	2	8	
	h) Any commercially fortified baby food like Cerelac, Nutren, Frisolac H, Weatabix, etc.?	h)	1	2	8	
	i) Bread, rice, noodles, porridge, macaroni, tuwo shinkafa, semo, masa, pap or other foods made from grains (e.g. millet, sorghum, maize, wheat, oats, etc.)?	i)	1	2	8	
	j) Pumpkin, carrots, squash, or sweet potatoes that are yellow or orange inside?	j)	1	2	8	
	k) Irish/white potatoes, white yams, cassava, plantain, cocoyam, garri, fufu, lafun, or any other foods made from roots?	k)	1	2	8	
	l) Any dark green, leafy vegetables like spinach, pumpkin leaf, ugu, zogale (moringa), yakuwa, soko, ewedu, oha leaf, lansir, yadiya, rama, tafasa, etc.?	l)	1	2	8	
	m) Ripe mangoes, ripe pawpaw, ripe passion fruit, dorowa, or red palm-nuts etc. ?	m)	1	2	8	
	n) Any other fruits or vegetables (e.g. banana, watermelon, apples, green beans, avocados, tomatoes)?	n)	1	2	8	
	o) Liver, kidney, heart, or other organ meats?	o)	1	2	8	
	p) Any meat, such as beef, mutton, pork, lamb, bat, bush rat/bush meat, kundi, kilishi, camel, chicken, or duck?	p)	1	2	8	
	q) Eggs?	q)	1	2	8	
	r) Fresh or dried fish or shellfish?	r)	1	2	8	
	s) Any foods made from beans, peas, lentils, or nuts like moimoi, akara?	s)	1	2	8	
	t) Cheese or other food made from milk?	t)	1	2	8	
	u) Any other solid, semi-solid, or soft food?	u)	1	2	8	

NO.	QUESTIONS AND FILTERS	CODING CATEGORIES	SKIP
651	CHECK 650 (CATEGORIES 'g' THROUGH 'u'):<br>NOT A SINGLE 'YES' ☐ AT LEAST ONE 'YES' ☐		→ 653
652	Did (NAME FROM 649) eat any solid, semi-solid, or soft foods yesterday during the day or at night?<br><br>IF 'YES' PROBE: What kind of solid, semi-solid or soft foods did (NAME) eat?	YES . . . . 1<br>(GO BACK TO 650 TO RECORD FOOD EATEN YESTERDAY)<br>(THEN CONTINUE TO 653)<br><br>NO 2	 <br><br><br>→ 653A
653	How many times did (NAME FROM 649) eat solid, semi-solid, or soft foods yesterday during the day or at night?<br><br>IF 7 OR MORE TIMES, RECORD '7'.	NUMBER OF TIMES . . . . . . ☐<br><br>DON'T KNOW 8	

653A Now I would like to ask you about foods and drinks that you ate or drank yesterday during the day or night, whether you ate it at home or anywhere else.
I am interested in whether you had the food items I will mention even if they were combined with other foods. For example, if you had a soup made with carrots, potatoes and meat, you should reply "yes" for each of these ingredients when I read you the list. However, if you consumed only the broth of a soup, but not the meat or vegetable, do not say "yes" for the meat or vegetable.
As I ask you about foods and drinks, please think of foods and drinks you had as snacks or small meals as well as during any main meals. Please also remember foods you may have eaten while preparing meals or preparing food for others.
Please do not include any food used in a small amount for seasoning or condiments (like spices, herbs or crayfish powder). I will ask you about those foods separately.

Yesterday during the day or at night, did you eat or drink:

		YES	NO	DK
**Any foods made from grains, like:**<br>a) Wheat, maize, rice, sorghum (guinea corn or dawa), millet (gero/jero), acha, spaghetti (talia), macaroni, noodles, bread,	a)	1	2	8
**Any vegetables or roots that are orange coloured inside like:**<br>b) Squash that is orange inside, pumpkin, carrot, red sweet pepper (tatase), sweet potato that is orange inside (orange flesh sweet potatoes)?	b)	1	2	8
**Any white roots and tubers or plantains, like:**<br>c) Yam, water yam, cocoyam, potato, cassava, tigernut flour,	c)	1	2	8
**Any dark green leafy vegetables, like:**<br>d) Ugu, bitter leaf (ewuro/ onugbu), zogale (moringa), yakuwa (sorrel leaves), soko, ewedu/ayoyo, afang/okazi, sweet potato leaves, cassava leaves, cocoyam leaves, amaranthus/spinach (green/tete), water leaf, oha leaf, karkashi, kuka (baobab, luru), lansir, yadiya, rama, tafasa, kanya, cress, lettuce, yanrin (wild spinach), eku gogoro, eku petere, ilasa (young okro leaves), igbagba, ebolo, atama, editan, scent leaf (ntong/nchuawu/ arigbe/aluluisi), chaya (iyana paja), egg plant leaves?	d)	1	2	8
**Any fruits that are dark yellow or orange inside, like:**<br>e) Ripe pawpaw (gwanda/ibeppe/okwuru oru/bobo), ripe mango, ripe passion fruit, dorowa (locust bean fruit), red palm fruit, hog plum (tsadan gida, iyeye, ngulungu), ripe cantaloupe, musk melon, monkey cola (ndiya), bush mango fruit (ugili/ogbono/mbupauyo) ?	e)	1	2	8
**Any other fruits, like:**<br>f) Apple, banana, watermelon, tangerine, grapes, avocado pear, oranges, pears, dates (dabino), guava, pineapple, grapefruit, coconut, African cherry/African star apple (agbalumo/udara/udala), breadfruit, cashew fruit, soursop, golden melon, baobab fruit (ose/nonkuku), figs, shea fruit, doum palm fruit (goruba)?	f)	1	2	8
**Any other vegetables, like:**<br>g) Cabbage, cucumber, fresh tomato, onion, green beans, green pepper, okro, garden egg, green peas, boiled or roasted fresh corn, beets, mushroom, ujuju?	g)	1	2	8

NO.	QUESTIONS AND FILTERS	CODING CATEGORIES				SKIP
	**Any meat made from animal organs, like:**<br>h) Liver, kidney, heart, gizzard?	h)	1	2	8	
	**Any other types of meat or poultry, like:**<br>i) Meat, chicken, and other bush meat/bird, kundi, kilishi, dambu nama, ponmo (cow skin)?	i)	1	2	8	
	**Any eggs**<br>j) Any eggs?	j)	1	2	8	
	**Any fish or seafood, whether fresh or dried, like:**<br>k) Fish, crab, lobster, cray fish, shrimp, stock fish (okporoko)?	k)	1	2	8	
	**Any beans or peas, like:**<br>l) Beans, chickpeas, soya beans, bambara nut (ebi-abo)?	l)	1	2	8	
	**Any nuts or seeds, like:**<br>m) Melon seed (egusi), pumpkin seeds (mkpuru anyu/ugboguru), walnuts, groundnuts, shea nut, cashew nuts, bush mango seeds (ogbono)?	m)	1	2	8	
	**Any milk or milk products, like:**<br>n) Milk, sour milk (nono), yogurt, cheese (wara)?	n)	1	2	8	
	**Any insects and other small protein foods, like:**<br>o) Winged termite (aku, esunsun, chinge, ako), cricket, snails (igbin/ejuna), sea snails (nkonko/isawuru), periwinkle, ogongo, akankwu, African palm weevil larva (monini/ekuku/okuka/uton/ [illegible]	o)	1	2	8	
	**Any red palm oil, like:**<br>p) Foods made with red palm oil, red palm nut, or red palm nut pulp sauces?	p)	1	2	8	
	**Any other oils and fats, like:**<br>q) Oil, fats or butter added to food or used for cooking, including vegetable oil, any other type of oil, butter, margarine (blue band), mayonnaise, shea butter, manshanu, extracted oils from nuts, fruits and seeds, and all animal fat?	q)	1	2	8	
	**Any savoury and fried snacks, like:**<br>r) Crisps and chips, fried dough (puffpuff), other fried snacks (chinchin, kulikuli, donkuwa)?	r)	1	2	8	
	**Any sweets, like:**<br>s) Chocolates, candies, cookies/sweet biscuits and cakes, sweet pastries or ice cream?	s)	1	2	8	
	**Any sugar-sweetened beverages, like:**<br>t) Sweetened fruit juices and "juice drinks", soft drinks/fizzy drinks, chocolate drinks(milo), malt drinks, sweet tea or coffee with	t)	1	2	8	
	**Any condiments and seasonings, like:**<br>u) Salt, Maggi, black pepper, alligator pepper, yaji, bay leaf, uziza, scent leaves, utazi, thyme, curry, ginger, garlic, cloves (kanafuru), tomato paste, ehuru, uyayak, uda, crayfish powder, locust bean used as seasoning, ogiri?	u)	1	2	8	
	**Any other beverages and foods, like:**<br>v) Coffee or tea if unsweetened, alcohol, clear broth, soup broth, olives, pickled cucumbers, herbal beverages/infusions (zobo), kunun aya, kunun dawa, water, kolanut, bitter kola?	v)	1	2	8	

SECTION 6. CHILD HEALTH AND NUTRITION

NO.	QUESTIONS AND FILTERS	CODING CATEGORIES	SKIP
653B	CHECK 215 AND 218, ALL ROWS: NUMBER OF CHILDREN BORN IN 2016-2018 LIVING WITH THE RESPONDENT<br>ONE OR MORE ☐ ↓<br>______________________<br>(NAME OF YOUNGEST CHILD LIVING WITH HER) ↓	NONE ☐	→ 701
654	The last time (NAME FROM 649) passed stools, what was done to dispose of the stools?	CHILD USED TOILET OR LATRINE . . . . 01<br>PUT/RINSED<br>INTO TOILET OR LATRI . . . . 02<br>PUT/RINSED<br>INTO DRAIN OR DITCH . . . . 03<br>THROWN INTO GARBA(. . . . . 04<br>BURIED . . . . 05<br>LEFT IN THE OPE . . . . 06<br>OTHER ______________________ 96<br>(SPECIFY)	

SECTION 7. MARRIAGE AND SEXUAL ACTIVITY

NO.	QUESTIONS AND FILTERS	CODING CATEGORIES	SKIP
701	Are you currently married or living together with a man as if married?	YES, CURRENTLY MARRIED . . . . 1<br>YES, LIVING WITH A MAN . . . . 2<br>NO, NOT IN UNION 3	1, 2 → 704
702	Have you ever been married or lived together with a man as if married?	YES, FORMERLY MARRIED . . . . 1<br>YES, LIVED WITH A MAN . . . . 2<br>NO 3	3 → 712
703	What is your marital status now: are you widowed, divorced, or separated?	WIDOWED . . . . 1<br>DIVORCED . . . . 2<br>SEPARATED 3	1, 2, 3 → 709
704	Is your (husband/partner) living with you now or is he staying elsewhere?	LIVING WITH HER . . . . 1<br>STAYING ELSEWHERE 2	
705	RECORD THE HUSBAND'S/PARTNER'S NAME AND LINE NUMBER FROM THE HOUSEHOLD QUESTIONNAIRE. IF HE IS NOT LISTED IN THE HOUSEHOLD, RECORD '00'.	NAME ____________________<br>LINE NO. . . . . [ ][ ]	
706	Does your (husband/partner) have other wives or does he live with other women as if married?	YES . . . . 1<br>NO . . . . 2<br>DON'T KNOW 8	2, 8 → 709
707	Including yourself, in total, how many wives or live-in partners does he have?	TOTAL NUMBER OF WIVES AND LIVE-IN PARTNERS . . . . [ ][ ]<br>DON'T KNOW 98	
708	Are you the first, second, … wife?	RANK . . . . [ ][ ]	
709	Have you been married or lived with a man only once or more than once?	ONLY ONCE . . . . 1<br>MORE THAN ONCE 2	
710	CHECK 709:<br>MARRIED/ LIVED WITH A MAN ONLY ONCE [ ] ↓<br>a) In what month and year did you start living with your (husband/partner)?<br>MARRIED/ LIVED WITH A MAN MORE THAN ONCE [ ] ↓<br>b) Now I would like to ask about your first (husband/partner). In what month and year did you start living with him?	MONTH . . . . [ ][ ]<br>DON'T KNOW MONTH . . . . 98<br>YEAR . . . . [ ][ ][ ][ ]<br>DON'T KNOW YEAR 9998	YEAR → 712
711	How old were you when you first started living with him?	AGE . . . . [ ][ ]	

SECTION 7. MARRIAGE AND SEXUAL ACTIVITY

NO.	QUESTIONS AND FILTERS	CODING CATEGORIES	SKIP
712	**CHECK FOR PRESENCE OF OTHERS. BEFORE CONTINUING, MAKE EVERY EFFORT TO ENSURE PRIVACY.**		
713	Now I would like to ask some questions about sexual activity in order to gain a better understanding of some important life issues. Let me assure you again that your answers are completely confidential and will not be told to anyone. If we should come to any question that you don't want to answer, just let me know and we will go to the next question. How old were you when you had sexual intercourse for the very first time?	NEVER HAD SEXUAL INTERCOURSE . . . . 00<br>AGE IN YEARS . . . . [ ][ ]	→731
714	I would like to ask you about your recent sexual activity. When was the last time you had sexual intercourse?<br><br>IF LESS THAN 12 MONTHS, ANSWER MUST BE RECORDED IN DAYS, WEEKS OR MONTHS. IF 12 MONTHS (ONE YEAR) OR MORE, ANSWER MUST BE RECORDED IN YEARS.	DAYS AGO . . . . 1 [ ][ ]<br>WEEKS AGO . . . . 2 [ ][ ]<br>MONTHS AGO . . . . 3 [ ][ ]<br>YEARS AGO . . . . 4 [ ][ ]	1–3 →716<br>4 →727

		LAST SEXUAL PARTNER	SECOND-TO-LAST SEXUAL PARTNER	THIRD-TO-LAST SEXUAL PARTNER
715	When was the last time you had sexual intercourse with this person?		DAYS AGO . . 1<br>WEEKS AGO . . 2<br>MONTHS AGO . . 3	DAYS AGO . . 1<br>WEEKS AGO . . 2<br>MONTHS AGO . . 3
716	The last time you had sexual intercourse with this person, was a condom used?	YES . . . . 1<br>NO . . . . 2<br>(SKIP TO 718)	YES . . . . 1<br>NO . . . . 2<br>(SKIP TO 718)	YES . . . . 1<br>NO . . . . 2<br>(SKIP TO 718)
717	Was a condom used every time you had sexual intercourse with this person in the last 12 months?	YES . . . . 1<br>NO 2	YES . . . . 1<br>NO 2	YES . . . . 1<br>NO 2
718	What was your relationship to this person with whom you had sexual intercourse?<br><br>IF BOYFRIEND: Were you living together as if married?<br><br>IF YES, RECORD '2'.<br>IF NO, RECORD '3'.	HUSBAND . . . . 1<br>LIVE-IN PARTNER . . . . . 2<br>BOYFRIEND NOT LIVING WITH RESPONDENT . . . . . 3<br>CASUAL ACQUAINTANCE . . 4<br>CLIENT/SEX WORKER . . 5<br>OTHER ______ 6<br>(SPECIFY)	HUSBAND . . . . 1<br>LIVE-IN PARTNER . . . . . 2<br>BOYFRIEND NOT LIVING WITH RESPONDENT . . . . . 3<br>CASUAL ACQUAINTANCE . . 4<br>CLIENT/SEX WORKER . . 5<br>OTHER ______ 6<br>(SPECIFY)	HUSBAND . . . . 1<br>LIVE-IN PARTNER . . . . . 2<br>BOYFRIEND NOT LIVING WITH RESPONDENT . . . . . 3<br>CASUAL ACQUAINTANCE . . 4<br>CLIENT/SEX WORKER . . 5<br>OTHER ______ 6<br>(SPECIFY)
719	How long ago did you first have sexual intercourse with this person?	DAYS AGO . . 1<br>WEEKS AGO . . 2<br>MONTHS AGO . . 3<br>YEARS AGO . . 4	DAYS AGO . . 1<br>WEEKS AGO . . 2<br>MONTHS AGO . . 3<br>YEARS AGO . . 4	DAYS AGO . . 1<br>WEEKS AGO . . 2<br>MONTHS AGO . . 3<br>YEARS AGO . . 4
720	How many times during the last 12 months did you have sexual intercourse with this person?<br>IF NON-NUMERIC ANSWER, PROBE TO GET AN ESTIMATE. IF NUMBER OF TIMES IS 95 OR MORE, RECORD '95'.	NUMBER OF TIMES	NUMBER OF TIMES	NUMBER OF TIMES . . . . .
721	How old is this person?	AGE OF PARTNER<br><br>DON'T KNOW 98	AGE OF PARTNER<br><br>DON'T KNOW 98	AGE OF PARTNER<br><br>DON'T KNOW 98
722	Apart from this person, have you had sexual intercourse with any other person in the last 12 months?	YES . . . . 1<br>(GO BACK TO 715 IN NEXT COLUMN)<br>NO . . . . 2<br>(SKIP TO 724)	YES . . . . 1<br>(GO BACK TO 715 IN NEXT COLUMN)<br>NO . . . . 2<br>(SKIP TO 724)	
723	In total, with how many different people have you had sexual intercourse in the last 12 months?<br>IF NON-NUMERIC ANSWER, PROBE TO GET AN ESTIMATE. IF NUMBER OF PARTNERS IS 95 OR MORE, RECORD '95'.			NUMBER OF PARTNERS LAST 12 MONTHS . .<br><br>DON'T KNOW 98

NO.	QUESTIONS AND FILTERS	CODING CATEGORIES	SKIP
724	CHECK 106: AGE 15-24 ☐ ↓	AGE 25-49 ☐	→ 727
725	CHECK 701: NOT IN A UNION ☐ ↓	CURRENTLY MARRIED/ LIVING WITH A MAN ☐	→ 727
726	In the past 12 months have you had sex or been sexually involved with anyone because he gave you or told you he would give you gifts, cash, or anything else?	YES . . . . 1<br>NO 2	
727	In total, with how many different people have you had sexual intercourse in your lifetime?<br><br>IF NON-NUMERIC ANSWER, PROBE TO GET AN ESTIMATE. IF NUMBER OF PARTNERS IS 95 OR MORE, RECORD '95'.	NUMBER OF PARTNERS IN LIFETIME . . . . ☐☐<br><br>DON'T KNOW 98	
728	CHECK 716, MOST RECENT PARTNER (FIRST COLUMN):<br>YES, CONDOM USED ☐ ↓	NO, CONDOM NOT USED ☐<br>NOT ASKED ☐	→ 731<br>→ 731
729	You told me that a condom was used the last time you had sex. What is the brand name of the condom used at that time?<br><br>IF BRAND NOT KNOWN, ASK TO SEE THE PACKAGE.	**MALE CONDOMS**<br>GOLD CIRCLE . . . . 01<br>DUREX . . . . 02<br>ROUGH RIDER . . . . 03<br>TWIN LOTUS . . . . 04<br>PLAIN CONDOMS . . . . 05<br>GO FLEX . . . . 06<br><br>OTHER ______ 96<br>(SPECIFY)<br>DON'T KNOW 98	

NO.	QUESTIONS AND FILTERS	CODING CATEGORIES	SKIP
730	From where did you obtain the condom the last time?<br><br>PROBE TO IDENTIFY TYPE OF SOURCE.<br><br>IF UNABLE TO DETERMINE IF PUBLIC OR PRIVATE SECTOR, WRITE THE NAME OF THE PLACE.<br><br>________________<br>(NAME OF PLACE)	**PUBLIC SECTOR**<br>GOVERNMENT HOSPITAL . . . . 11<br>GOVERNMENT HEALTH CENTER . . . . 12<br>FAMILY PLANNING CLINIC . . . . 13<br>MOBILE CLINIC . . . . 14<br>FIELDWORKER . . . . 15<br>OTHER PUBLIC SECTOR<br>________________ 16<br>(SPECIFY)<br><br>**PRIVATE MEDICAL SECTOR**<br>PRIVATE HOSPITAL/CLINIC . . . . 21<br>PHARMACY . . . . 22<br>CHEMIST/PMS . . . . 23<br>PRIVATE DOCTOR . . . . 24<br>MOBILE CLINIC . . . . 25<br>FIELDWORKER . . . . 26<br>OTHER PRIVATE MEDICAL SECTOR<br>________________ 27<br>(SPECIFY)<br><br>**OTHER SOURCE**<br>SHOP . . . . 31<br>CHURCH . . . . 32<br>FRIEND/RELATIVE . . . . 33<br>NGO . . . . 34<br><br>OTHER ________________ 96<br>(SPECIFY)<br>DON'T KNOW 98	
731	PRESENCE OF OTHERS DURING THIS SECTION.	YES NO<br>CHILDREN <10 . . . . 1 2<br>MALE ADULTS . . . . 1 2<br>FEMALE ADULTS 1 2	

SECTION 8. FERTILITY PREFERENCES

NO.	QUESTIONS AND FILTERS	CODING CATEGORIES	SKIP
801	CHECK 304:<br>NEITHER STERILIZED ☐ ↓	HE OR SHE STERILIZED ☐	→ 813
802	CHECK 226:<br>PREGNANT ☐ ↓	NOT PREGNANT OR UNSURE ☐	→ 804
803	Now I have some questions about the future. After the child you are expecting now, would you like to have another child, or would you prefer not to have any more children?	HAVE ANOTHER CHILD . . . . 1<br>NO MORE . . . . 2<br>UNDECIDED/DON'T KNOW 8	→ 805<br>→ 812 (2, 8)
804	Now I have some questions about the future. Would you like to have (a/another) child, or would you prefer not to have any (more) children?	HAVE (A/ANOTHER) CHILD . . . . 1<br>NO MORE/NONE . . . . 2<br>SAYS SHE CAN'T GET PREGNANT . . . . 3<br>UNDECIDED/DON'T KNOW 8	 → 807<br>→ 813<br>→ 811
805	CHECK 226:<br>NOT PREGNANT OR UNSURE ☐ ↓ / PREGNANT ☐ ↓<br>a) How long would you like to wait from now before the birth of (a/another) child?<br>b) After the birth of the child you are expecting now, how long would you like to wait before the birth of another child?	MONTHS . . . . 1 ☐☐<br>YEARS . . . . 2 ☐☐<br>SOON/NOW . . . . 993<br>SAYS SHE CAN'T GET PREGNANT . . . . 994<br>AFTER MARRIAGE . . . . 995<br>OTHER __________ (SPECIFY) 996<br>DON'T KNOW 998	 <br>→ 811<br>→ 813<br>→ 811 (995, 996, 998)
806	CHECK 226:<br>NOT PREGNANT OR UNSURE ☐ ↓	PREGNANT ☐	→ 812
807	CHECK 303: USING A CONTRACEPTIVE METHOD?<br>NOT CURRENTLY USING ☐ ↓	CURRENTLY USING ☐	→ 813
808	CHECK 805:<br>'24' OR MORE MONTHS OR '02' OR MORE YEARS ☐ ↓<br>NOT ASKED ☐ ↓	'00-23' MONTHS OR '00-01' YEAR ☐	→ 812
809	CHECK 714:<br>DAYS, WEEKS OR MONTHS AGO ☐ ↓	YEARS AGO ☐<br>NOT ASKED ☐	→ 811<br>→ 811

SECTION 8. FERTILITY PREFERENCES

NO.	QUESTIONS AND FILTERS	CODING CATEGORIES	SKIP
810	CHECK 804:<br>WANTS TO HAVE A/ANOTHER CHILD ☐<br>a) You have said that you do not want (a/another) child soon. Can you tell me why you are not using a method to prevent pregnancy?<br>Any other reason?<br>WANTS NO MORE/ NONE ☐<br>b) You have said that you do not want any (more) children. Can you tell me why you are not using a method to prevent pregnancy?<br>Any other reason?<br>RECORD ALL REASONS MENTIONED.	NOT MARRIED . . . A<br>**FERTILITY-RELATED REASONS**<br>NOT HAVING SEX . . . B<br>INFREQUENT SEX . . . C<br>MENOPAUSAL/HYSTERECTOMY . . . D<br>CAN'T GET PREGNANT . . . E<br>NOT MENSTRUATED SINCE LAST BIRTH . . . F<br>BREASTFEEDING . . . G<br>UP TO GOD/FATALISTIC . . . H<br>**OPPOSITION TO USE**<br>RESPONDENT OPPOSED . . . I<br>HUSBAND/PARTNER OPPOSED . . . J<br>OTHERS OPPOSED . . . K<br>RELIGIOUS PROHIBITION . . . L<br>**LACK OF KNOWLEDGE**<br>KNOWS NO METHOD . . . M<br>KNOWS NO SOURCE . . . N<br>**METHOD-RELATED REASONS**<br>SIDE EFFECTS/HEALTH CONCERNS . . . O<br>LACK OF ACCESS/TOO FAR . . . P<br>COSTS TOO MUCH . . . Q<br>PREFERRED METHOD NOT AVAILABLE . . . R<br>NO METHOD AVAILABLE . . . S<br>INCONVENIENT TO USE . . . T<br>INTERFERES WITH BODY'S NORMAL PROCESSES . . . U<br>OTHER ______ X<br>(SPECIFY)<br>DON'T KNOW . . . Z	
811	CHECK 303: USING A CONTRACEPTIVE METHOD?<br>NOT ASKED ☐<br>NO, NOT CURRENTLY USING ☐<br>YES, CURRENTLY USING ☐ → 813		813
812	Do you think you will use a contraceptive method to delay or avoid pregnancy at any time in the future?	YES . . . 1<br>NO . . . 2<br>DON'T KNOW . . . 8	
813	CHECK 216:<br>HAS LIVING CHILDREN ☐<br>a) If you could go back to the time you did not have any children and could choose exactly the number of children to have in your whole life, how many would that be?<br>NO LIVING CHILDREN ☐<br>b) If you could choose exactly the number of children to have in your whole life, how many would that be?<br>PROBE FOR A NUMERIC RESPONSE.	NONE . . . 00<br>NUMBER . . . [ ][ ]<br>OTHER ______ 96<br>(SPECIFY)	NONE → 815<br>OTHER → 815
814	How many of these children would you like to be boys, how many would you like to be girls and for how many would it not matter if it's a boy or a girl?	BOYS [ ][ ] GIRLS [ ][ ] EITHER [ ][ ]<br>NUMBER . .<br>OTHER ______ 96<br>(SPECIFY)	

SECTION 8. FERTILITY PREFERENCES

NO.	QUESTIONS AND FILTERS	CODING CATEGORIES	SKIP
815	In the last few months have you:<br>a) Heard about family planning on the radio?<br>b) Seen anything about family planning on the television?<br>c) Read about family planning in a newspaper or magazine?<br>d) Received a voice or text message about family planning on a mobile phone?<br>e) Read/heard from social media (facebook, twitter, etc.)?<br>f) Read about family planning in a poster?<br>g) Read about family planning in a leaflet or brochures?<br>h) Heard about family planning from town crier?<br>i) Heard about family planning from mobile public announcement?	YES NO<br>a) RADIO . . . . . 1 2<br>b) TELEVISION . . . . . 1 2<br>c) NEWSPAPER OR MAGAZINE . . . . . 1 2<br>d) MOBILE PHONE . . . . . 1 2<br>e) SOCIAL MEDIA . . . . . 1 2<br>f) POSTER . . . . . 1 2<br>g) LEAFLET OR BROCHURE . . . . . 1 2<br>h) TOWN CRIER . . . . . 1 2<br>i) MOBILE PUBLIC ANNOUNCEMEN 1 2	
815A	CHECK 815:<br>AT LEAST ONE `YES' (HAS HEARD OR READ MESSAGE) ☐↓<br>NOT A SINGLE `YES' (HAS NOT HEARD OR READ MESSAGE) ☐ → 817		817
816	Please tell me which family planning messages you have heard or seen in the past few months?<br><br>PROBE: Any others?	AS FOR ME AND MY PARTNER WE `DEY KAMPE' WITH FEMALE CONDOM . . . . . A<br>UNSPACED CHILDREN MAKES THE GOING TOUGH FOR THE LOVE OF YOUR FAMILY, GO FOR CHILD SPACING TODAY . . . . . B<br>WELL-SPACED CHILDREN ARE EVERY PARENT'S JOY . . . . . C<br>IT'S NOT TOO LATE TO PREVENT UNWANTED PREGNANCY . . . . . D<br>WHY IS YOUR WIFE LOOKING SO GOO . . . . . E<br>OTHER ______ X<br>(SPECIFY)	
817	CHECK 701:<br>YES, CURRENTLY MARRIED ☐↓<br>YES, LIVING WITH A MAN ☐↓<br>NO, NOT IN A UNION ☐ → 901		901
818	CHECK 303: USING A CONTRACEPTIVE METHOD?<br>CURRENTLY USING ☐↓<br>NOT CURRENTLY USING ☐ → 820<br>NOT ASKED ☐ → 822		820<br>822
819	Would you say that using contraception is mainly your decision, mainly your (husband's/partner's) decision, or did you both decide together?	MAINLY RESPONDENT . . . . . 1<br>MAINLY HUSBAND/PARTNER . . . . . 2<br>JOINT DECISION . . . . . 3<br>OTHER ______ 6<br>(SPECIFY)	→ 821
820	Would you say that not using contraception is mainly your decision, mainly your (husband's/partner's) decision, or did you both decide together?	MAINLY RESPONDENT . . . . . 1<br>MAINLY HUSBAND/PARTNER . . . . . 2<br>JOINT DECISION . . . . . 3<br>OTHER ______ 6<br>(SPECIFY)	
821	CHECK 304:<br>NEITHER ARE STERILIZED ☐↓<br>HE OR SHE ARE STERILIZED ☐ → 901		901
822	Does your (husband/partner) want the same number of children that you want, or does he want more or fewer than you want?	SAME NUMBER . . . . . 1<br>MORE CHILDREN . . . . . 2<br>FEWER CHILDREN . . . . . 3<br>DON'T KNOW 8	

SECTION 9. HUSBAND'S BACKGROUND AND WOMAN'S WORK

NO.	QUESTIONS AND FILTERS	CODING CATEGORIES	SKIP
901	CHECK 701:<br>CURRENTLY MARRIED/ LIVING WITH A MAN ☐ ↓	NOT IN UNION ☐	→ 909
902	How old was your (husband/partner) on his last birthday?	AGE IN COMPLETED YEARS . . . . ☐☐	
903	Did your (husband/partner) ever attend school?	YES . . . . 1<br>NO 2	 → 906
904	What was the highest level of school he attended: primary, secondary, or higher?	PRIMARY . . . . 1<br>SECONDARY . . . . 2<br>HIGHER . . . . 3<br>DON'T KNOW 8	 <br><br>→ 906
905	What was the highest Class/Year he completed at that level?<br>IF COMPLETED LESS THAN ONE YEAR AT THAT LEVEL, RECORD '00'.	CLASS/YEAR . . . . ☐☐<br>DON'T KNOW 98	
906	Has your (husband/partner) done any work in the last 7 days?	YES . . . . 1<br>NO . . . . 2<br>DON'T KNOW 8	→ 908
907	Has your (husband/partner) done any work in the last 12 months?	YES . . . . 1<br>NO . . . . 2<br>DON'T KNOW 8	 → 909 (NO, DON'T KNOW)
908	What is your (husband's/partner's) occupation? That is, what kind of work does he mainly do?	__________<br>__________ ☐☐<br>__________	
909	Aside from your own housework, have you done any work in the last seven days?	YES . . . . 1<br>NO 2	→ 913
910	As you know, some women take up jobs for which they are paid in cash or kind. Others sell things, have a small business or work on the family farm or in the family business. In the last seven days, have you done any of these things or any other work?	YES . . . . 1<br>NO 2	→ 913
911	Although you did not work in the last seven days, do you have any job or business from which you were absent for leave, illness, vacation, maternity leave, or any other such reason?	YES . . . . 1<br>NO 2	→ 913
912	Have you done any work in the last 12 months?	YES . . . . 1<br>NO 2	 → 917
913	What is your occupation? That is, what kind of work do you mainly do?	__________<br>__________ ☐☐<br>__________	

NO.	QUESTIONS AND FILTERS	CODING CATEGORIES	SKIP
914	Do you do this work for a member of your family, for someone else, or are you self-employed?	FOR FAMILY MEMBER . . . 1<br>FOR SOMEONE ELSE . . . 2<br>SELF-EMPLOYED . . . 3	
915	Do you usually work throughout the year, or do you work seasonally, or only once in a while?	THROUGHOUT THE YEAR . . . 1<br>SEASONALLY/PART OF THE YEAR . . . 2<br>ONCE IN A WHILE . . . 3	
916	Are you paid in cash or kind for this work or are you not paid at all?	CASH ONLY . . . 1<br>CASH AND KIND . . . 2<br>IN KIND ONLY . . . 3<br>NOT PAID . . . 4	
917	CHECK 701:<br>CURRENTLY MARRIED/LIVING WITH A MAN ☐ ↓	NOT IN UNION ☐	→ 925
918	CHECK 916:<br>CODE '1' OR '2' CIRCLED ☐ ↓	OTHER ☐	→ 921
919	Who usually decides how the money you earn will be used: you, your (husband/partner), or you and your (husband/partner) jointly?	RESPONDENT . . . 1<br>HUSBAND/PARTNER . . . 2<br>RESPONDENT AND HUSBAND/PARTNER JOINTLY . . . 3<br>OTHER ______ 6<br>(SPECIFY)	
920	Would you say that the money that you earn is more than what your (husband/partner) earns, less than what he earns, or about the same?	MORE THAN HIM . . . 1<br>LESS THAN HIM . . . 2<br>ABOUT THE SAME . . . 3<br>HUSBAND/PARTNER HAS NO EARNINGS . . . 4<br>DON'T KNOW . . . 8	4 → 922
921	Who usually decides how your (husband's/partner's) earnings will be used: you, your (husband/partner), or you and your (husband/partner) jointly?	RESPONDENT . . . 1<br>HUSBAND/PARTNER . . . 2<br>RESPONDENT AND HUSBAND/PARTNER JOINTLY . . . 3<br>HUSBAND/PARTNER HAS NO EARNINGS . . . 4<br>OTHER ______ 6<br>(SPECIFY)	
922	Who usually makes decisions about health care for yourself: you, your (husband/partner), you and your (husband/partner) jointly, or someone else?	RESPONDENT . . . 1<br>HUSBAND/PARTNER . . . 2<br>RESPONDENT AND HUSBAND/PARTNER JOINTLY . . . 3<br>SOMEONE ELSE . . . 4<br>OTHER . . . 6	
923	Who usually makes decisions about making major household purchases?	RESPONDENT . . . 1<br>HUSBAND/PARTNER . . . 2<br>RESPONDENT AND HUSBAND/PARTNER JOINTLY . . . 3<br>SOMEONE ELSE . . . 4<br>OTHER . . . 6	

SECTION 9. HUSBAND'S BACKGROUND AND WOMAN'S WORK

NO.	QUESTIONS AND FILTERS	CODING CATEGORIES	SKIP
924	Who usually makes decisions about visits to your family or relatives?	RESPONDENT . . . . 1<br>HUSBAND/PARTNER . . . . 2<br>RESPONDENT AND HUSBAND/PARTNER JOINTLY . . . . 3<br>SOMEONE ELSE . . . . 4<br>OTHER 6	
925	Do you own this or any other house either alone or jointly with someone else?	ALONE ONLY . . . . 1<br>JOINTLY ONLY . . . . 2<br>BOTH ALONE AND JOINTLY . . . . 3<br>DOES NOT OWN 4	4 → 928
926	Do you have a title deed for any house you own?	YES . . . . 1<br>NO . . . . 2<br>DON'T KNOW 8	2, 8 → 928
927	Is your name on the title deed?	YES . . . . 1<br>NO . . . . 2<br>DON'T KNOW 8	
928	Do you own any agricultural or non-agricultural land either alone or jointly with someone else?	ALONE ONLY . . . . 1<br>JOINTLY ONLY . . . . 2<br>BOTH ALONE AND JOINTLY . . . . 3<br>DOES NOT OWN 4	4 → 931
929	Do you have a title deed for any land you own?	YES . . . . 1<br>NO . . . . 2<br>DON'T KNOW 8	2, 8 → 931
930	Is your name on the title deed?	YES . . . . 1<br>NO . . . . 2<br>DON'T KNOW 8	
931	PRESENCE OF OTHERS AT THIS POINT (PRESENT AND LISTENING, PRESENT BUT NOT LISTENING, OR NOT PRESENT)	PRES./LISTEN. | PRES./NOT LISTEN. | NOT PRES.<br>CHILDREN < 10 . . . . 1 2 3<br>HUSBAND . . . . 1 2 3<br>OTHER MALES . . . . 1 2 3<br>OTHER FEMALES 1 2 3	
932	In your opinion, is a husband justified in hitting or beating his wife in the following situations:<br>a) If she goes out without telling him?<br>b) If she neglects the children?<br>c) If she argues with him?<br>d) If she refuses to have sex with him?<br>e) If she burns the food?	YES | NO | DK<br>a) GOES OUT . . . . 1 2 8<br>b) NEGLECTS CHILDREN . . 1 2 8<br>c) ARGUES . . . . 1 2 8<br>d) REFUSES SEX . . . . 1 2 8<br>e) BURNS FOOD 1 2 8	

SECTION 10. HIV/AIDS

NO.	QUESTIONS AND FILTERS	CODING CATEGORIES	SKIP
1001	Now I would like to talk about something else. Have you ever heard of HIV or AIDS?	YES . . . . 1<br>NO 2	 → 1042
1002	HIV is the virus that can lead to AIDS. Can people reduce their chance of getting HIV by having just one uninfected sex partner who has no other sex partners?	YES . . . . 1<br>NO . . . . 2<br>DON'T KNOW 8	
1003	Can people get HIV from mosquito bites?	YES . . . . 1<br>NO . . . . 2<br>DON'T KNOW 8	
1004	Can people reduce their chance of getting HIV by using a condom every time they have sex?	YES . . . . 1<br>NO . . . . 2<br>DON'T KNOW 8	
1005	Can people get HIV by sharing food with a person who has HIV?	YES . . . . 1<br>NO . . . . 2<br>DON'T KNOW 8	
1006	Can people get HIV because of witchcraft or other supernatural means?	YES . . . . 1<br>NO . . . . 2<br>DON'T KNOW 8	
1007	Is it possible for a healthy-looking person to have HIV?	YES . . . . 1<br>NO . . . . 2<br>DON'T KNOW 8	
1008	Can HIV be transmitted from a mother to her baby:<br>a) During pregnancy?<br>b) During delivery?<br>c) By breastfeeding?	YES NO DK<br>a) DURING PREGNANCY . . 1 2 8<br>b) DURING DELIVERY . . . . . 1 2 8<br>c) BREASTFEEDING 1 2 8	
1009	CHECK 1008:<br>AT LEAST ONE 'YES' ☐ ↓ OTHER ☐ →		1011
1010	Are there any special drugs that a doctor or a nurse can give to a woman infected with HIV to reduce the risk of transmission to the baby?	YES . . . . 1<br>NO . . . . 2<br>DON'T KNOW 8	
1011	CHECK 208 AND 215:<br>NO BIRTHS ☐ →<br>LAST BIRTH IN 2016-2018 ☐ ↓ LAST BIRTH IN 2015 OR EARLIER ☐ →		1035<br>1035
1012	CHECK 408 FOR LAST BIRTH:<br>HAD ANTENATAL CARE ☐ ↓ NO ANTENATAL CARE ☐ →		1035
1014	During any of the antenatal visits for your last birth were you given any information about:<br>a) Babies getting HIV from their mother?<br>b) Things that you can do to prevent getting HIV?<br>c) Getting tested for HIV?	YES NO DK<br>a) HIV FROM MOTHER . . 1 2 8<br>b) THINGS TO DO . . . . 1 2 8<br>c) TESTED FOR HIV 1 2 8	
1035	Would you buy fresh vegetables from a shopkeeper or vendor if you knew that this person had HIV?	YES . . . . 1<br>NO . . . . 2<br>DON'T KNOW/NOT SURE/DEPENDS 8	

SECTION 10. HIV/AIDS

NO.	QUESTIONS AND FILTERS	CODING CATEGORIES	SKIP
1036	Do you think children living with HIV should be allowed to attend school with children who do not have HIV?	YES . . . 1<br>NO . . . 2<br>DON'T KNOW/NOT SURE/DEPENDS . . . 8	
1037	Do you think people hesitate to take an HIV test because they are afraid of how other people will react if the test result is positive for HIV?	YES . . . 1<br>NO . . . 2<br>DON'T KNOW/NOT SURE/DEPENDS . . . 8	
1038	Do people talk badly about people living with HIV, or who are thought to be living with HIV?	YES . . . 1<br>NO . . . 2<br>DON'T KNOW/NOT SURE/DEPENDS . . . 8	
1039	Do people living with HIV, or thought to be living with HIV, lose the respect of other people?	YES . . . 1<br>NO . . . 2<br>DON'T KNOW/NOT SURE/DEPENDS . . . 8	
1040	Do you agree or disagree with the following statement: I would be ashamed if someone in my family had HIV.	AGREE . . . 1<br>DISAGREE . . . 2<br>DON'T KNOW/NOT SURE/DEPENDS . . . 8	
1041	Do you fear that you could get HIV if you come into contact with the saliva of a person living with HIV?	YES . . . 1<br>NO . . . 2<br>SAYS SHE HAS HIV . . . 3<br>DON'T KNOW/NOT SURE/DEPENDS . . . 8	
1042	CHECK 1001:<br>HEARD ABOUT HIV OR AIDS ☐ / NOT HEARD ABOUT HIV OR AIDS ☐<br>a) Apart from HIV, have you heard about other infections that can be transmitted through sexual contact?<br>b) Have you heard about infections that can be transmitted through sexual contact?	YES . . . 1<br>NO . . . 2	
1043	CHECK 713:<br>HAS HAD SEXUAL INTERCOURSE ☐ / NEVER HAD SEXUAL INTERCOURSE ☐ → 1051		1051
1044	CHECK 1042: HEARD ABOUT OTHER SEXUALLY TRANSMITTED INFECTIONS?<br>YES ☐ / NO ☐ → 1046		1046
1045	Now I would like to ask you some questions about your health in the last 12 months. During the last 12 months, have you had a disease which you got through sexual contact?	YES . . . 1<br>NO . . . 2<br>DON'T KNOW . . . 8	
1046	Sometimes women experience a bad-smelling abnormal genital discharge. During the last 12 months, have you had a bad-smelling abnormal genital discharge?	YES . . . 1<br>NO . . . 2<br>DON'T KNOW . . . 8	
1047	Sometimes women have a genital sore or ulcer. During the last 12 months, have you had a genital sore or ulcer?	YES . . . 1<br>NO . . . 2<br>DON'T KNOW . . . 8	
1048	CHECK 1045, 1046, AND 1047:<br>HAS HAD AN INFECTION (ANY 'YES') ☐ / HAS NOT HAD AN INFECTION OR DOES NOT KNOW ☐ → 1051		1051
1049	The last time you had (PROBLEM FROM 1045/1046/1047), did you seek any kind of advice or treatment?	YES . . . 1<br>NO . . . 2	 → 1051

NO.	QUESTIONS AND FILTERS	CODING CATEGORIES	SKIP
1050	Where did you go?<br><br>Any other place?<br><br>PROBE TO IDENTIFY THE TYPE OF SOURCE.<br><br>IF UNABLE TO DETERMINE IF PUBLIC OR PRIVATE SECTOR, WRITE THE NAME OF THE PLACE.<br><br>__________<br>(NAME OF PLACE)	**PUBLIC SECTOR**<br>GOVERNMENT HOSPITAL . . . . . A<br>GOVERNMENT HEALTH CENTER . . . . . B<br>STAND-ALONE HTS CENTER . . . . . C<br>FAMILY PLANNING CLINIC . . . . . D<br>MOBILE HTS SERVICES . . . . . E<br>OTHER PUBLIC SECTOR<br>__________ F<br>(SPECIFY)<br>**PRIVATE MEDICAL SECTOR**<br>PRIVATE HOSPITAL/CLINIC/<br>PRIVATE DOCTOR . . . . . G<br>STAND-ALONE HTS CENTER . . . . . H<br>PHARMACY . . . . . I<br>CHEMIST/PMS STORE . . . . . J<br>MOBILE HTS SERVICES . . . . . K<br>OTHER PRIVATE MEDICAL SECTOR<br>__________ L<br>(SPECIFY)<br>**OTHER SOURCE**<br>SHOP . . . . . M<br><br>OTHER __________ X<br>(SPECIFY)	
1051	If a wife knows her husband has a disease that she can get during sexual intercourse, is she justified in asking that they use a condom when they have sex?	YES . . . . . 1<br>NO . . . . . 2<br>DON'T KNOW 8	
1052	Is a wife justified in refusing to have sex with her husband when she knows he has sex with other women?	YES . . . . . 1<br>NO . . . . . 2<br>DON'T KNOW 8	
1053	CHECK 701:<br>CURRENTLY MARRIED/ LIVING WITH A MAN ☐ ↓	NOT IN UNION ☐	→ 1101
1054	Can you say no to your (husband/partner) if you do not want to have sexual intercourse?	YES . . . . . 1<br>NO . . . . . 2<br>DEPENDS/NOT SURE 8	
1055	Could you ask your (husband/partner) to use a condom if you wanted him to?	YES . . . . . 1<br>NO . . . . . 2<br>DEPENDS/NOT SURE 8	

SECTION 11. OTHER HEALTH ISSUES

NO.	QUESTIONS AND FILTERS	CODING CATEGORIES	SKIP
1101	Now I would like to ask you some other questions relating to health matters. Have you had an injection for any reason in the last 12 months?<br><br>IF YES: How many injections have you had?<br><br>IF NUMBER OF INJECTIONS IS 90 OR MORE, OR DAILY FOR 3 MONTHS OR MORE, RECORD '90'. IF NON-NUMERIC ANSWER, PROBE TO GET AN ESTIMATE.	NUMBER OF INJECTIONS . . . . [ ][ ]<br><br>NONE 00	 <br>→1104
1102	Among these injections, how many were administered by a doctor, a nurse, a pharmacist, a dentist, or any other health worker?<br><br>IF NUMBER OF INJECTIONS IS 90 OR MORE, OR DAILY FOR 3 MONTHS OR MORE, RECORD '90'. IF NON-NUMERIC ANSWER, PROBE TO GET AN ESTIMATE.	NUMBER OF INJECTIONS . . . . [ ][ ]<br><br>NONE 00	 <br>→1104
1103	The last time you got an injection from a health worker, did he/she take the syringe and needle from a new, unopened package?	YES . . . . 1<br>NO . . . . 2<br>DON'T KNOW 8	
1104	Do you currently smoke cigarettes every day, some days, or not at all?	EVERY DAY . . . . 1<br>SOME DAYS . . . . 2<br>NOT AT ALL 3	 2, 3 → 1106
1105	On average, how many cigarettes do you currently smoke each day?	NUMBER OF CIGARETTES . . . . [ ][ ]	
1106	Do you currently smoke or use any other type of tobacco every day, some days, or not at all?	EVERY DAY . . . . 1<br>SOME DAYS . . . . 2<br>NOT AT ALL 3	 <br>→1108
1107	What other type of tobacco do you currently smoke or use?<br><br>RECORD ALL MENTIONED.	KRETEKS . . . . A<br>PIPES FULL OF TOBACCO . . . . B<br>CIGARS, CHEROOTS, OR CIGARILLOS . . . . C<br>WATER PIPE . . . . D<br>SNUFF BY MOUTH . . . . E<br>SNUFF BY NOSE . . . . F<br>CHEWING TOBACCO . . . . G<br>BETEL QUID WITH TOBACCO . . . . H<br><br>OTHER ______________________ X<br>(SPECIFY)	
1108	Many different factors can prevent women from getting medical advice or treatment for themselves. When you are sick and want to get medical advice or treatment, is each of the following a big problem or not a big problem:<br><br>a) Getting permission to go to the doctor?<br><br>b) Getting money needed for advice or treatment?<br><br>c) The distance to the health facility?<br><br>d) Not wanting to go alone?	BIG PROBLEM / NOT A BIG PROBLEM<br><br>a) PERMISSION TO GO . . . . . 1 2<br><br>b) GETTING MONEY . . . . 1 2<br><br>c) DISTANCE . . . . 1 2<br><br>d) GO ALONE 1 2	

SECTION 11. OTHER HEALTH ISSUES

NO.	QUESTIONS AND FILTERS	CODING CATEGORIES				SKIP
1108A	I am going to ask you about your opinion on behavior/practice on reducing the risk of malaria. Please tell me whether you agree or disagree with the following statements:		AGREE	DISAGREE	DON'T KNOW	
	b) The medicine given to pregnant women to prevent malaria works well to keep the mother healthy	b)	1	2	8	
	c) The medicine given to pregnant women to prevent malaria works well to keep the baby healthy when it is born	c)	1	2	8	
	d) The malaria tests are the only way to know if someone really has malaria or not	d)	1	2	8	
	f) Even if the malaria test shows that the fever is not caused by malaria, I will still seek out treatment for malaria because I don't trust the test result	f)	1	2	8	
	i) When the entire course of malaria medicine is taken, the disease will be fully cured	i)	1	2	8	
1108B	I am going to ask you about your opinion on consequences of malaria. Please tell me whether you agree or disagree with the following statements:		AGREE	DISAGREE	DON'T KNOW	
	a) Every case of malaria can potentially lead to death	a)	1	2	8	
	c) You don't worry about malaria because it can be easily treated	c)	1	2	8	
	d) You know people who have become dangerously sick with malaria.	d)	1	2	8	
	f) Only weak children can die from malaria	f)	1	2	8	
1109	Are you covered by any health insurance?	YES . . . . . 1<br>NO 2				 → 1200
1110	What type of health insurance are you covered by?<br><br>RECORD ALL MENTIONED.	MUTUAL HEALTH ORGANIZATION/ COMMUNITY-BASED HEALTH INSURANCE . . . . . A<br>HEALTH INSURANCE THROUGH EMPLOYER . . . . . B<br>SOCIAL SECURITY . . . . . C<br>OTHER PRIVATELY PURCHASED COMMERCIAL HEALTH INSURANCE . . . . . D<br><br>OTHER ________ X<br>(SPECIFY)				

SECTION 12. FEMALE GENITAL CUTTING/MUTILATION

NO.	QUESTIONS AND FILTERS	CODING CATEGORIES	SKIP
1200	CHECK COVER PAGE: HOUSEHOLD SELECTED FOR MAN'S SURVEY?<br>HOUSEHOLD NOT SELECTED FOR MAN'S SURVEY ☐ ↓	HOUSEHOLD SELECTED ☐	→ 1401
1201	Now I would like to ask some questions about a practice known as female circumcision, that is, a practice in which a girl may have part of her genitals cut, for example, excision of the clitoris and the labia minora, scraping of tissue surrounding the vaginal orifice (angurya cuts) or cutting of the vagina (gishiri cuts) and even use of corrosive sunstances or herbs into vagina to tighten or narrow it or to cause bleeding.<br><br>Have you ever heard about any of these practices?	YES . . . . 1<br>NO 2	 → 1301
1202	Have you yourself ever had any of these procedures performed on you?	YES . . . . 1<br>NO . . . . 2<br>DON'T KNOW 8	 NO/DON'T KNOW → 1208
1203	Now I would like to ask you what was done to you at that time. Was any flesh removed from the genital area?	YES . . . . 1<br>NO . . . . 2<br>DON'T KNOW 8	→ 1205
1204	Was the genital area just nicked without removing any flesh?	YES . . . . 1<br>NO . . . . 2<br>DON'T KNOW 8	
1205	Was your genital area sewn closed?	YES . . . . 1<br>NO . . . . 2<br>DON'T KNOW 8	
1205A	Which type of procedure was performed on you?<br>a) Removal of clitoris along with partial or total excision of the labia minora?<br>b) Infibulation: removal of clitoris, labia minora and adjacent medial part of labia majora and stitching it?<br>c) Scraping of tissue surrounding the vaginal orifice (eg. Angurya cuts etc.)?<br>d) Cutting of the vagina (eg. Gishiri cuts etc)?	YES / NO / DK<br>a) REMOVAL OF CLITORIS . . 1 2 8<br>b) INFIBULATION . . . . 1 2 8<br>c) ANGURYA . . . . 1 2 8<br>d) GISHIRI 1 2 8	
1205B	Have you ever used corrosive substances or herbs into vagina with the aim of tightening or narrowing it or to cause bleeding?	YES . . . . 1<br>NO . . . . 2<br>DON'T KNOW 8	
1206	How old were you when this procedure (GC6A/GC6B) was performed for the first time?<br><br>IF THE RESPONDENT DOES NOT KNOW THE EXACT AGE, PROBE TO GET AN ESTIMATE.	AGE IN COMPLETED YEARS . . . . . [ ][ ]<br>AS A BABY/DURING INFANCY . . . . 95<br>DON'T KNOW 98	
1207	Who performed this procedure?	TRADITIONAL<br>TRADITIONAL CURCUMCISER . . . . 11<br>TRADITIONAL BIRTH ATTENDANT . . . . 12<br>OTHER TRADITIONAL ______ 16<br>(SPECIFY)<br>HEALTH PROFESSIONAL<br>DOCTOR . . . . 21<br>NURSE/MIDWIFE . . . . 22<br>OTHER HEALTH PROFESSIONAL ______ 26<br>(SPECIFY)<br>DON'T KNOW 98	
1208	CHECK 213, 215 AND 216:<br>HAS ONE OR MORE LIVING DAUGHTERS BORN IN 2003 OR LATER ☐ ↓	HAS NO LIVING DAUGHTERS BORN IN 2003 OR LATER ☐	→ 1216

CHECK 213, 215 AND 216: ENTER IN THE TABLE THE BIRTH HISTORY NUMBER AND NAME OF EACH LIVING DAUGHTER BORN IN 2003 OR LATER. ASK THE QUESTIONS ABOUT ALL OF THESE DAUGHTERS. BEGIN WITH THE YOUNGEST DAUGHTER. (IF THERE ARE MORE THAN 3 DAUGHTERS, USE ADDITIONAL QUESTIONNAIRES).

1209 Now I would like to ask you some questions about your (daughter/daughters).

		YOUNGEST LIVING DAUGHTER	NEXT-TO-YOUNGEST LIVING DAUGHTER	SECOND-TO-YOUNGEST LIVING DAUGHTER
1210	BIRTH HISTORY NUMBER AND NAME OF EACH LIVING DAUGHTER BORN IN 2003 OR LATER.	BIRTH HISTORY NUMBER [ ][ ]<br>NAME ________	BIRTH HISTORY NUMBER [ ][ ]<br>NAME ________	BIRTH HISTORY NUMBER [ ][ ]<br>NAME ________
1211	Is (NAME OF DAUGHTER) circumcised?	YES . . . . . 1<br>NO . . . . . 2 (GO TO 1211 IN NEXT COLUMN; OR IF NO MORE DAUGHTERS, GO TO 1216)	YES . . . . . 1<br>NO . . . . . 2 (GO TO 1211 IN NEXT COLUMN; OR IF NO MORE DAUGHTERS, GO TO 1216)	YES . . . . . 1<br>NO . . . . . 2 (GO TO 1211 IN NEXT COLUMN; OR IF NO MORE DAUGHTERS, GO TO 1216)
1212	How old was (NAME OF DAUGHTER) when she was circumcised?<br><br>IF THE RESPONDENT DOES NOT KNOW THE AGE, PROBE TO GET AN ESTIMATE.	AGE IN COMPLETED YRS . . [ ][ ]<br>DON'T KNOW 98	AGE IN COMPLETED YRS . . [ ][ ]<br>DON'T KNOW 98	AGE IN COMPLETED YRS . . [ ][ ]<br>DON'T KNOW 98
1213	Was her genital area sewn closed?	YES . . . . . 1<br>NO . . . . . 2<br>DON'T KNOW 8	YES . . . . . 1<br>NO . . . . . 2<br>DON'T KNOW 8	YES . . . . . 1<br>NO . . . . . 2<br>DON'T KNOW 8
1214	Who performed the circumcision?	**TRADITIONAL**<br>TRADITIONAL CIRCUMCISER . . 11<br>TRAD. BIRTH ATTENDANT . . 12<br>OTHER TRAD. ________ 16 (SPECIFY)<br><br>**HEALTH PROFESSIONAL**<br>DOCTOR . . . . 21<br>NURSE/MIDWIFE . . 22<br>OTHER HEALTH PROFESSIONAL ________ 26 (SPECIFY)<br><br>DON'T KNOW 98	**TRADITIONAL**<br>TRADITIONAL CIRCUMCISER . . 11<br>TRAD. BIRTH ATTENDANT . . 12<br>OTHER TRAD. ________ 16 (SPECIFY)<br><br>**HEALTH PROFESSIONAL**<br>DOCTOR . . . . 21<br>NURSE/MIDWIFE . . 22<br>OTHER HEALTH PROFESSIONAL ________ 26 (SPECIFY)<br><br>DON'T KNOW 98	**TRADITIONAL**<br>TRADITIONAL CIRCUMCISER . . 11<br>TRAD. BIRTH ATTENDANT . . 12<br>OTHER TRAD. ________ 16 (SPECIFY)<br><br>**HEALTH PROFESSIONAL**<br>DOCTOR . . . . 21<br>NURSE/MIDWIFE . . 22<br>OTHER HEALTH PROFESSIONAL ________ 26 (SPECIFY)<br><br>DON'T KNOW 98
1215		GO BACK TO 1211 IN NEXT COLUMN; OR, IF NO MORE DAUGHTERS, GO TO 1216.	GO BACK TO 1211 IN NEXT COLUMN; OR, IF NO MORE DAUGHTERS, GO TO 1216.	GO BACK TO 1211 IN NEXT COLUMN; OR, IF NO MORE DAUGHTERS, GO TO 1216.

1216	Do you believe that female circumcision is required by your religion?	YES . . . . . 1<br>NO . . . . . 2<br>NO RELIGION . . . . . 3<br>DON'T KNOW 8	
1217	Do you think that female circumcision should be continued, or should it be stopped?	CONTINUED . . . . . 1<br>STOPPED . . . . . 2<br>DEPENDS . . . . . 3<br>DON'T KNOW 8	

SECTION 13. FISTULA

NO.	QUESTIONS AND FILTERS	CODING CATEGORIES	SKIP
1301	Sometimes a woman can have a problem of constant leakage of urine or stool from her vagina during the day and night. This problem usually occurs after a difficult childbirth, but may also occur after a sexual assault or after pelvic surgery.<br><br>Have you ever experienced a constant leakage of urine or stool from your vagina during the day and night?	YES . . . . . 1<br>NO 2	1 → 1303
1302	Have you ever heard of this problem?	YES . . . . . 1<br>NO 2	1, 2 → 1401
1303	Did this problem start after you delivered a baby or had a stillbirth?	AFTER DELIVERED BABY . . . . . 1<br>AFTER HAD STILLBIRTH . . . . . 2<br>NEITHER 3	3 → 1305
1304	Did this problem start after a normal labor and delivery, or after a very difficult labor and delivery?	NORMAL LABOR/DELIVERY . . . . . 1<br>VERY DIFFICULT LABOR/DELIVERY 2	1, 2 → 1306
1305	What do you think caused this problem?	SEXUAL ASSAULT . . . . . 1<br>PELVIC SURGERY . . . . . 2<br>OTHER ______ 6<br>(SPECIFY)<br>DON'T KNOW 8	8 → 1307
1306	How many days after (CAUSE OF PROBLEM FROM 1303 OR 1305) did the leakage start?<br><br>ENTER '90' IF 90 DAYS OR MORE.	NUMBER OF DAYS AFTER DELIVERY/OTHER EVENT . . . . . [ ][ ]	
1306A	How old were you when you experienced this problem?	AGE IN YEARS . . . . . [ ][ ]	
1307	Have you sought treatment for this condition?	YES . . . . . 1<br>NO 2	1 → 1309
1308	Why have you not sought treatment?	DO NOT KNOW CAN BE FIXED . . . . . A<br>DO NOT KNOW WHERE TO GO . . . . . B<br>TOO EXPENSIVE . . . . . C<br>TOO FAR . . . . . D<br>POOR QUALITY OF CARE . . . . . E<br>COULD NOT GET PERMISSION . . . . . F<br>EMBARRASSMENT . . . . . G<br>PROBLEM DISAPPEARED . . . . . H<br>OTHER ______ X<br>(SPECIFY)	→ 1401
1309	From whom did you last seek treatment?	**HEALTH PROFESSIONAL**<br>DOCTOR . . . . . 1<br>NURSE/MIDWIFE . . . . . 2<br>**OTHER PERSON**<br>COMMUNITY/VILLAGE HEALTH WORKER . . . . . 3<br>OTHER ______ 6<br>(SPECIFY)	
1310	Did you have an operation to fix the problem?	YES . . . . . 1<br>NO 2	
1311	Did the treatment stop the leakage completely?<br><br>IF NO: Did the treatment reduce the leakage?	YES, STOPPED COMPLETELY . . . . . 1<br>NOT STOPPED BUT REDUCED . . . . . 2<br>NOT STOPPED AT ALL . . . . . 3<br>DID NOT RECEIVE TREATMENT 4	
1312	Are there any (other) women in your household who suffer from obstetric fistula?	YES . . . . . 1<br>NO 2	2 → 1401
1313	How many (other) women in your household suffer from obstetric fistula?	NUMBER . . . . . [ ][ ]<br>DON'T KNOW 98	

1401	Now I would like to ask you some questions about your brothers and sisters born to your natural mother, including those who are living with you, those living elsewhere and those who have died. From our experience in prior surveys, we know it may sometimes be difficult to establish a complete list of all the children born to your natural mother. We will work together to draw the most complete list and work to recall all your siblings. Could you please now give me the names of all of your brothers and sisters born to your natural mother.<br>DO NOT FILL IN THE ORDER NUMBER YET.	

NAME	ORDER NUMBER	NAME	ORDER NUMBER
a ______		k ______	
b ______		l ______	
c ______		m ______	
d ______		n ______	
e ______		o ______	
f ______		p ______	
g ______		q ______	
h ______		r ______	
i ______		s ______	
j ______		t ______	

1402	CHECK 1401:<br>ONE OR MORE BROTHERS OR SISTERS LISTED ☐ NO BROTHERS OR SISTERS LISTED ☐	→ 1404
1403	READ THE NAMES OF THE BROTHERS AND SISTERS TO THE RESPONDENT AND AFTER THE LAST ONE ASK: Are there any other brothers and sisters from the same mother that you have not mentioned?<br>NO ☐ YES ☐ → LIST ADDITIONAL BROTHERS AND SISTERS IN 1401.	
1404	Sometimes people forget to mention children born to their natural mother because they do not live with them or they do not see them very often. Are there any brothers or sisters who do not live with you that you have not mentioned?<br>NO ☐ YES ☐ → LIST ADDITIONAL BROTHERS AND SISTERS IN 1401.	
1405	Sometimes people forget to mention children born to their natural mother because they have died. Are there any brothers or sisters who died that you have not mentioned?<br>NO ☐ YES ☐ → LIST ADDITIONAL BROTHERS AND SISTERS IN 1401.	
1406	Some people have brothers or sisters from the same mother but a different father. Are there any brothers or sisters born to your natural mother, but who have a different natural father, that you have not mentioned?<br>NO ☐ YES ☐ → LIST ADDITIONAL BROTHERS AND SISTERS IN 1401.	
1407	COUNT THE NUMBER OF BROTHERS AND SISTERS RECORDED IN 1401.	TOTAL BROTHERS AND SISTERS . .

1408	CHECK 1407:<br>Just to make make sure that I have this right: Your mother had in TOTAL ________ births, excluding you, during her lifetime. Is that correct?<br>YES ☐ NO ☐ → PROBE AND CORRECT 1401 AND/OR 1407	
1409	CHECK 1407:<br>ONE OR MORE BROTHERS/SISTERS ☐ NO BROTHER OR SISTER ☐ →	1501
1410	Please tell me, which brother or sister was born first? And which was born next?<br>RECORD '01' FOR THE ORDER NUMBER IN 1401 FOR THE FIRST BROTHER OR SISTER, '02' FOR THE SECOND, AND SO ON UNTIL YOU HAVE RECORDED THE ORDER NUMBER FOR ALL BROTHERS AND SISTERS.	
1411	How many births did your mother have before you were born? — NUMBER OF PRECEDING BIRTHS ☐☐	

1412	LIST THE BROTHERS AND SISTERS ACCORDING TO THE ORDER NUMBER IN 1401. ASK 1413 TO 1424 FOR ONE BROTHER OR SISTER BEFORE ASKING ABOUT THE NEXT BROTHER OR SISTER. IF THERE ARE MORE THAN 12 BROTHERS AND SISTERS, USE AN ADDITIONAL QUESTIONNAIRE.						
1413	NAME OF BROTHER OR SISTER	(01) ________	(02) ________	(03) ________	(04) ________	(05) ________	(06) ________
1414	Is (NAME) male or female?	MALE . . . 1<br>FEMALE . 2	MALE . . . 1<br>FEMALE . 2	MALE . . . 1<br>FEMALE . 2	MALE . . . 1<br>FEMALE . 2	MALE . . . 1<br>FEMALE . 2	MALE . . . 1<br>FEMALE . 2
1415	Is (NAME) still alive?	YES . . . . . 1<br>NO . . . . . 2<br>GO TO 1417<br>DK . . . . . 8<br>GO TO (02)	YES . . . . . 1<br>NO . . . . . 2<br>GO TO 1417<br>DK . . . . . 8<br>GO TO (03)	YES . . . . . 1<br>NO . . . . . 2<br>GO TO 1417<br>DK . . . . . 8<br>GO TO (04)	YES . . . . . 1<br>NO . . . . . 2<br>GO TO 1417<br>DK . . . . . 8<br>GO TO (05)	YES . . . . . 1<br>NO . . . . . 2<br>GO TO 1417<br>DK . . . . . 8<br>GO TO (06)	YES . . . . . 1<br>NO . . . . . 2<br>GO TO 1417<br>DK . . . . . 8<br>GO TO (07)
1416	How old is (NAME)?	GO TO (02)	GO TO (03)	GO TO (04)	GO TO (05)	GO TO (06)	GO TO (07)
1417	How many years ago did (NAME) die?						
1418	How old was (NAME) when (he/she) died?<br>IF DON'T KNOW, PROBE AND ASK ADDITIONAL QUESTIONS TO GET AN ESTIMATE	IF MALE OR DIED BEFORE 12 YEARS OF AGE, GO TO 1423	IF MALE OR DIED BEFORE 12 YEARS OF AGE, GO TO 1423	IF MALE OR DIED BEFORE 12 YEARS OF AGE, GO TO 1423	IF MALE OR DIED BEFORE 12 YEARS OF AGE, GO TO 1423	IF MALE OR DIED BEFORE 12 YEARS OF AGE, GO TO 1423	IF MALE OR DIED BEFORE 12 YEARS OF AGE, GO TO 1423
1419	Was (NAME) pregnant when she died?	YES . . . . . 1<br>GO TO 1422A<br>NO 2	YES . . . . . 1<br>GO TO 1422A<br>NO 2	YES . . . . . 1<br>GO TO 1422A<br>NO 2	YES . . . . . 1<br>GO TO 1422A<br>NO 2	YES . . . . . 1<br>GO TO 1422A<br>NO 2	YES . . . . . 1<br>GO TO 1422A<br>NO 2
1420	Did (NAME) die during childbirth?	YES . . . . . 1<br>GO TO 1422A<br>NO 2	YES . . . . . 1<br>GO TO 1422A<br>NO 2	YES . . . . . 1<br>GO TO 1422A<br>NO 2	YES . . . . . 1<br>GO TO 1422A<br>NO 2	YES . . . . . 1<br>GO TO 1422A<br>NO 2	YES . . . . . 1<br>GO TO 1422A<br>NO 2
1421	Did (NAME) die within two months after the end of a pregnancy or childbirth?	YES . . . . . 1<br>NO . . . . . 2<br>GO TO 1423	YES . . . . . 1<br>NO . . . . . 2<br>GO TO 1423	YES . . . . . 1<br>NO . . . . . 2<br>GO TO 1423	YES . . . . . 1<br>NO . . . . . 2<br>GO TO 1423	YES . . . . . 1<br>NO . . . . . 2<br>GO TO 1423	YES . . . . . 1<br>NO . . . . . 2<br>GO TO 1423
1422	How many days after the end of the pregnancy did (NAME)						
1422A	In which State did (NAME)<br>WRITE THE STATE CODE.						
1422B	CHECK 1420:	YES / NO/ NOT ASKED<br>GO TO (02)	YES / NO/ NOT ASKED<br>GO TO (03)	YES / NO/ NOT ASKED<br>GO TO (04)	YES / NO/ NOT ASKED<br>GO TO (05)	YES / NO/ NOT ASKED<br>GO TO (06)	YES / NO/ NOT ASKED<br>GO TO (07)
1423	Was (NAME)'s death due to an act of violence?	YES . . . . . 1<br>GO TO (02)<br>NO 2	YES . . . . . 1<br>GO TO (03)<br>NO 2	YES . . . . . 1<br>GO TO (04)<br>NO 2	YES . . . . . 1<br>GO TO (05)<br>NO 2	YES . . . . . 1<br>GO TO (06)<br>NO 2	YES . . . . . 1<br>GO TO (07)<br>NO 2
1424	Was (NAME)'s death due to an accident?	YES . . . . . 1<br>NO . . . . . 2<br>GO TO (02)	YES . . . . . 1<br>NO . . . . . 2<br>GO TO (03)	YES . . . . . 1<br>NO . . . . . 2<br>GO TO (04)	YES . . . . . 1<br>NO . . . . . 2<br>GO TO (05)	YES . . . . . 1<br>NO . . . . . 2<br>GO TO (06)	YES . . . . . 1<br>NO . . . . . 2<br>GO TO (07)

IF NO MORE BROTHERS OR SISTERS, GO TO NEXT SECTION.

1412	LIST THE BROTHERS AND SISTERS ACCORDING TO THE ORDER NUMBER IN 1401. ASK 1413 TO 1424 FOR ONE BROTHER OR SISTER BEFORE ASKING ABOUT THE NEXT BROTHER OR SISTER. IF THERE ARE MORE THAN 12 BROTHERS AND SISTERS, USE AN ADDITIONAL QUESTIONNAIRE.						
1413	NAME OF BROTHER OR SISTER	(07) ________	(08) ________	(09) ________	(10) ________	(11) ________	(12) ________
1414	Is (NAME) male or female?	MALE . . . 1<br>FEMALE . 2	MALE . . . 1<br>FEMALE . 2	MALE . . . 1<br>FEMALE . 2	MALE . . . 1<br>FEMALE . 2	MALE . . . 1<br>FEMALE . 2	MALE . . . 1<br>FEMALE . 2
1415	Is (NAME) still alive?	YES . . . . . 1<br>NO . . . . . 2<br>GO TO 1417<br>DK . . . . . 8<br>GO TO (08)	YES . . . . . 1<br>NO . . . . . 2<br>GO TO 1417<br>DK . . . . . 8<br>GO TO (09)	YES . . . . . 1<br>NO . . . . . 2<br>GO TO 1417<br>DK . . . . . 8<br>GO TO (10)	YES . . . . . 1<br>NO . . . . . 2<br>GO TO 1417<br>DK . . . . . 8<br>GO TO (11)	YES . . . . . 1<br>NO . . . . . 2<br>GO TO 1417<br>DK . . . . . 8<br>GO TO (12)	YES . . . . . 1<br>NO . . . . . 2<br>GO TO 1417<br>DK . . . . . 8<br>GO TO (13)
1416	How old is (NAME)?	☐☐<br>GO TO (08)	☐☐<br>GO TO (09)	☐☐<br>GO TO (10)	☐☐<br>GO TO (11)	☐☐<br>GO TO (12)	☐☐<br>GO TO (13)
1417	How many years ago did (NAME) die?	☐☐	☐☐	☐☐	☐☐	☐☐	☐☐
1418	How old was (NAME) when (he/she) died?<br>IF DON'T KNOW, PROBE AND ASK ADDITIONAL QUESTIONS TO GET AN ESTIMATE	☐☐<br>IF MALE OR DIED BEFORE 12 YEARS OF AGE, GO TO 1423	☐☐<br>IF MALE OR DIED BEFORE 12 YEARS OF AGE, GO TO 1423	☐☐<br>IF MALE OR DIED BEFORE 12 YEARS OF AGE, GO TO 1423	☐☐<br>IF MALE OR DIED BEFORE 12 YEARS OF AGE, GO TO 1423	☐☐<br>IF MALE OR DIED BEFORE 12 YEARS OF AGE, GO TO 1423	☐☐<br>IF MALE OR DIED BEFORE 12 YEARS OF AGE, GO TO 1423
1419	Was (NAME) pregnant when she died?	YES . . . . . 1<br>GO TO 1422A<br>NO 2	YES . . . . . 1<br>GO TO 1422A<br>NO 2	YES . . . . . 1<br>GO TO 1422A<br>NO 2	YES . . . . . 1<br>GO TO 1422A<br>NO 2	YES . . . . . 1<br>GO TO 1422A<br>NO 2	YES . . . . . 1<br>GO TO 1422A<br>NO 2
1420	Did (NAME) die during childbirth?	YES . . . . . 1<br>GO TO 1422A<br>NO 2	YES . . . . . 1<br>GO TO 1422A<br>NO 2	YES . . . . . 1<br>GO TO 1422A<br>NO 2	YES . . . . . 1<br>GO TO 1422A<br>NO 2	YES . . . . . 1<br>GO TO 1422A<br>NO 2	YES . . . . . 1<br>GO TO 1422A<br>NO 2
1421	Did (NAME) die within two months after the end of a pregnancy or childbirth?	YES . . . . . 1<br>NO . . . . . 2<br>GO TO 1423	YES . . . . . 1<br>NO . . . . . 2<br>GO TO 1423	YES . . . . . 1<br>NO . . . . . 2<br>GO TO 1423	YES . . . . . 1<br>NO . . . . . 2<br>GO TO 1423	YES . . . . . 1<br>NO . . . . . 2<br>GO TO 1423	YES . . . . . 1<br>NO . . . . . 2<br>GO TO 1423
1422	How many days after the end of the pregnancy did (NAME)	☐☐	☐☐	☐☐	☐☐	☐☐	☐☐
1422A	In which State did (NAME)<br>WRITE THE STATE CODE.	☐☐	☐☐	☐☐	☐☐	☐☐	☐☐
1422B	CHECK 1420:	YES ☐ GO TO (08)<br>NO/NOT ASKED ☐	YES ☐ GO TO (09)<br>NO/NOT ASKED ☐	YES ☐ GO TO (10)<br>NO/NOT ASKED ☐	YES ☐ GO TO (11)<br>NO/NOT ASKED ☐	YES ☐ GO TO (12)<br>NO/NOT ASKED ☐	YES ☐ GO TO (13)<br>NO/NOT ASKED ☐
1423	Was (NAME)'s death due to an act of violence?	YES . . . . . 1<br>GO TO (08)<br>NO 2	YES . . . . . 1<br>GO TO (09)<br>NO 2	YES . . . . . 1<br>GO TO (10)<br>NO 2	YES . . . . . 1<br>GO TO (11)<br>NO 2	YES . . . . . 1<br>GO TO (12)<br>NO 2	YES . . . . . 1<br>GO TO (13)<br>NO 2
1424	Was (NAME)'s death due to an accident?	YES . . . . . 1<br>NO . . . . . 2<br>GO TO (08)	YES . . . . . 1<br>NO . . . . . 2<br>GO TO (09)	YES . . . . . 1<br>NO . . . . . 2<br>GO TO (10)	YES . . . . . 1<br>NO . . . . . 2<br>GO TO (11)	YES . . . . . 1<br>NO . . . . . 2<br>GO TO (12)	YES . . . . . 1<br>NO . . . . . 2<br>GO TO (13)

IF NO MORE BROTHERS OR SISTERS, GO TO NEXT SECTION.

SECTION 15: DOMESTIC VIOLENCE MODULE

NO.	QUESTIONS AND FILTERS	CODING CATEGORIES	SKIP
1500	CHECK COVER PAGE: WOMAN SELECTED FOR DV MODULE?<br>WOMAN SELECTED FOR THIS SECTION ☐ ↓<br>WOMAN NOT SELECTED ☐		→ 1533
1501	CHECK FOR PRESENCE OF OTHERS:<br>DO NOT CONTINUE UNTIL PRIVACY IS ENSURED.<br>PRIVACY OBTAINED .... 1 ↓<br>PRIVACY NOT POSSIBLE 2		→ 1532
1501A	READ TO THE RESPONDENT:<br>Now I would like to ask you questions about some other important aspects of a woman's life. You may find some of these questions very personal. However, your answers are crucial for helping to understand the condition of women in Nigeria. Let me assure you that your answers are completely confidential and will not be told to anyone and no one else in your household will know that you were asked these questions. If I ask you any question you don't want to answer, just let me know and I will go on to the next question.		
1502	CHECK 701 AND 702:<br>CURRENTLY MARRIED/ LIVING WITH A MAN ☐ ↓<br>FORMERLY MARRIED/ LIVED WITH A MAN (READ IN PAST TENSE AND USE 'LAST' WITH 'HUSBAND/PARTNER') ☐ ↓<br>NEVER MARRIED/ NEVER LIVED WITH A MAN ☐		→ 1516
1503	First, I am going to ask you about some situations which happen to some women. Please tell me if these apply to your relationship with your (last) (husband/partner)?		

Question		YES	NO	DK
a) He (is/was) jealous or angry if you (talk/talked) to other men?	JEALOUS	1	2	8
b) He frequently (accuses/accused) you of being unfaithful?	ACCUSES	1	2	8
c) He (does/did) not permit you to meet your female friends?	NOT MEET FRIENDS ..	1	2	8
d) He (tries/tried) to limit your contact with your family?	NO FAMILY	1	2	8
e) He (insists/insisted) on knowing where you (are/were) at all times?	WHERE YOU ARE	1	2	8

1504 Now I need to ask some more questions about your relationship with your (last) (husband/partner).

A. Did your (last) (husband/partner) ever:

B. How often did this happen during the last 12 months: often, only sometimes, or not at all?

	EVER	OFTEN	SOME-TIMES	NOT IN LAST 12 MONTHS
a) say or do something to humiliate you in front of others?	YES 1 →<br>NO 2 ↓	1	2	3
b) threaten to hurt or harm you or someone you care about?	YES 1 →<br>NO 2 ↓	1	2	3
c) insult you or make you feel bad about yourself?	YES 1 →<br>NO 2 ↓	1	2	3

NO.	QUESTIONS AND FILTERS		CODING CATEGORIES			SKIP
1505	A. Did your (last) (husband/partner) ever do any of the following things to you:		B. How often did this happen during the last 12 months: often, only sometimes, or not at all?			
		EVER	OFTEN	SOME-TIMES	NOT IN LAST 12 MONTHS	
	a) push you, shake you, or throw something at you?	YES 1 → NO 2 ↓	1	2	3	
	b) slap you?	YES 1 → NO 2 ↓	1	2	3	
	c) twist your arm or pull your hair?	YES 1 → NO 2 ↓	1	2	3	
	d) punch you with his fist or with something that could hurt you?	YES 1 → NO 2 ↓	1	2	3	
	e) kick you, drag you, or beat you up?	YES 1 → NO 2 ↓	1	2	3	
	f) try to choke you or burn you on purpose?	YES 1 → NO 2 ↓	1	2	3	
	g) threaten or attack you with a knife, gun, or other weapon?	YES 1 → NO 2 ↓	1	2	3	
	h) physically force you to have sexual intercourse with him when you did not want to?	YES 1 → NO 2 ↓	1	2	3	
	i) physically force you to perform any other sexual acts you did not want to?	YES 1 → NO 2 ↓	1	2	3	
	j) force you with threats or in any other way to perform sexual acts you did not want to?	YES 1 → NO 2 ↓	1	2	3	
1506	CHECK 1505A (a-j): AT LEAST ONE 'YES' ☐ ↓		NOT A SINGLE 'YES' ☐ →			1509
1507	How long after you first (got married/started living together) with your (last) (husband/partner) did (this/any of these things) first happen? IF LESS THAN ONE YEAR, RECORD '00'.		NUMBER OF YEARS . . . . . . ☐☐ BEFORE MARRIAGE/BEFORE LIVING TOGETHER 95			
1508	Did the following ever happen as a result of what your (last) (husband/partner) did to you:					
	a) You had cuts, bruises, or aches?		YES . . . . 1 NO 2			
	b) You had eye injuries, sprains, dislocations, or burns?		YES . . . . 1 NO 2			
	c) You had deep wounds, broken bones, broken teeth, or any other serious injury?		YES . . . . 1 NO 2			
1509	Have you ever hit, slapped, kicked, or done anything else to physically hurt your (last) (husband/partner) at times when he was not already beating or physically hurting you?		YES . . . . 1 NO . . . . 2 →			 1511

NO.	QUESTIONS AND FILTERS	CODING CATEGORIES	SKIP
1510	In the last 12 months, how often have you done this to your (last) (husband/partner): often, only sometimes, or not at all?	OFTEN . . . 1<br>SOMETIMES . . . 2<br>NOT AT ALL . . . 3	
1511	Does (did) your (last) (husband/partner) drink alcohol?	YES . . . 1<br>NO . . . 2	 → 1513
1512	How often does (did) he get drunk: often, only sometimes, or never?	OFTEN . . . 1<br>SOMETIMES . . . 2<br>NEVER . . . 3	
1513	Are (Were) you afraid of your (last) (husband/partner): most of the time, sometimes, or never?	MOST OF THE TIME AFRAID . . . 1<br>SOMETIMES AFRAID . . . 2<br>NEVER AFRAID . . . 3	
1514	CHECK 709:<br>MARRIED MORE THAN ONCE ☐ ↓	MARRIED ONLY ONCE ☐ →	1516

1515	A. So far we have been talking about the behavior of your (current/last) (husband/partner). Now I want to ask you about the behavior of any previous (husband/partner).	EVER	B. How long ago did this last happen? 0 - 11 MONTHS AGO	12+ MONTHS AGO	DON'T REMEMBER
	a) Did any previous (husband/partner) ever hit, slap, kick, or do anything else to hurt you physically?	YES 1 →<br>NO 2 ↓	1	2	3
	b) Did any previous (husband/partner) physically force you to have intercourse or perform any other sexual acts against your will?	YES 1 →<br>NO 2 ↓	1	2	3
	c) Did any previous (husband/partner) humiliate you in front of others, threaten to hurt you or someone you care about, or insult you or make you feel bad about yourself?	YES 1 →<br>NO 2 ↓	1	2	3

NO.	QUESTIONS AND FILTERS	CODING CATEGORIES	SKIP
1516	CHECK 701 AND 702:<br>EVER MARRIED/EVER LIVED WITH A MAN ☐ ↓<br>a) From the time you were 15 years old has anyone other than (your/any) (husband/partner) hit you, slapped you, kicked you, or done anything else to hurt you physically?<br><br>NEVER MARRIED/NEVER LIVED WITH A MAN ☐ ↓<br>b) From the time you were 15 years old has anyone hit you, slapped you, kicked you, or done anything else to hurt you physically?	YES . . . 1<br>NO . . . 2<br>REFUSED TO ANSWER/ NO ANSWER . . . 3	 } → 1519

NO.	QUESTIONS AND FILTERS	CODING CATEGORIES	SKIP
1517	Who has hurt you in this way?<br><br>Anyone else?<br><br>RECORD ALL MENTIONED.	MOTHER/STEP-MOTHER . . . . A<br>FATHER/STEP-FATHER . . . . B<br>SISTER/BROTHER . . . . C<br>DAUGHTER/SON . . . . D<br>OTHER RELATIVE . . . . E<br>CURRENT BOYFRIEND . . . . F<br>FORMER BOYFRIEND . . . . G<br>MOTHER-IN-LAW . . . . H<br>FATHER-IN-LAW . . . . I<br>OTHER IN-LAW . . . . J<br>TEACHER . . . . K<br>EMPLOYER/SOMEONE AT WORK . . . . . L<br>POLICE/SOLDIER . . . . M<br><br>OTHER ______________________ X<br>(SPECIFY)	
1518	In the last 12 months, how often has (this person/have these persons) physically hurt you: often, only sometimes, or not at all?	OFTEN . . . . 1<br>SOMETIMES . . . . 2<br>NOT AT ALL 3	
1519	CHECK 201, 226, AND 230:<br><br>EVER BEEN PREGNANT ☐ ('YES' ON 201 OR 226 OR 230) ↓	NEVER BEEN PREGNANT ☐ →	1522
1520	Has any one ever hit, slapped, kicked, or done anything else to hurt you physically while you were pregnant?	YES . . . . 1<br>NO 2	 → 1522
1521	Who has done any of these things to physically hurt you while you were pregnant?<br><br>Anyone else?<br><br>RECORD ALL MENTIONED.	CURRENT HUSBAND/PARTNER . . . . . A<br>MOTHER/STEP-MOTHER . . . . B<br>FATHER/STEP-FATHER . . . . C<br>SISTER/BROTHER . . . . D<br>DAUGHTER/SON . . . . E<br>OTHER RELATIVE . . . . F<br>FORMER HUSBAND/PARTNER . . . . . G<br>CURRENT BOYFRIEND . . . . H<br>FORMER BOYFRIEND . . . . I<br>MOTHER-IN-LAW . . . . J<br>FATHER-IN-LAW . . . . K<br>OTHER IN-LAW . . . . L<br>TEACHER . . . . M<br>EMPLOYER/SOMEONE AT WORK . . . . . N<br>POLICE/SOLDIER . . . . O<br><br>OTHER ______________________ X<br>(SPECIFY)	
1522	CHECK 701 AND 702:<br><br>EVER MARRIED/EVER LIVED WITH A MAN ☐ ↓	NEVER MARRIED/NEVER LIVED WITH A MAN ☐ →	1522B

NO.	QUESTIONS AND FILTERS	CODING CATEGORIES	SKIP
1522A	Now I want to ask you about things that may have been done to you by someone other than (your/any) (husband/partner). At any time in your life, as a child or as an adult, has anyone ever forced you in any way to have sexual intercourse or perform any other sexual acts when you did not want to?	YES . . . . . 1<br>NO . . . . . 2<br>REFUSED TO ANSWER/ NO ANSWER 3	1 → 1523<br>2, 3 → 1524A
1522B	At any time in your life, as a child or as an adult, has anyone ever forced you in any way to have sexual intercourse or perform any other sexual acts when you did not want to?	YES . . . . . 1<br>NO . . . . . 2<br>REFUSED TO ANSWER/ NO ANSWER 3	2, 3 → 1526
1523	Who was the person who was forcing you the very first time this happened?	CURRENT HUSBAND/PARTNER . . . . . 01<br>FORMER HUSBAND/PARTNER . . . . . 02<br>CURRENT/FORMER BOYFRIEND . . . . . 03<br>FATHER/STEP-FATHER . . . . . 04<br>BROTHER/STEP-BROTHER . . . . . 05<br>OTHER RELATIVE . . . . . 06<br>IN-LAW . . . . . 07<br>OWN FRIEND/ACQUAINTANCE . . . . . 08<br>FAMILY FRIEND . . . . . 09<br>TEACHER . . . . . 10<br>EMPLOYER/SOMEONE AT WORK . . . . . 11<br>POLICE/SOLDIER . . . . . 12<br>PRIEST/RELIGIOUS LEADER . . . . . 13<br>STRANGER . . . . . 14<br>OTHER ______ 96 (SPECIFY)	
1524	CHECK 701 AND 702:<br>EVER MARRIED/EVER LIVED WITH A MAN ☐ ↓ | NEVER MARRIED/NEVER LIVED WITH A MAN ☐ ↓<br>a) In the last 12 months, has anyone other than (your/any) (husband/partner) physically forced you to have sexual intercourse when you did not want to?<br>b) In the last 12 months has anyone physically forced you to have sexual intercourse when you did not want to?	YES . . . . . 1<br>NO 2	1, 2 → 1525
1524A	CHECK 1505A (h-j) and 1515A(b)<br>AT LEAST ONE 'YES' ☐ ↓ | NOT A SINGLE 'YES' ☐ →		1526
1525	CHECK 701 AND 702:<br>EVER MARRIED/EVER LIVED WITH A MAN ☐ ↓ | NEVER MARRIED/NEVER LIVED WITH A MAN ☐ ↓<br>a) How old were you the first time you were forced to have sexual intercourse or perform any other sexual acts by anyone, including (your/any) husband/partner?<br>b) How old were you the first first time you were forced to have sexual intercourse or perform any other sexual acts?	AGE IN COMPLETED YEARS . . . . . [ ][ ]<br>DON'T KNOW 98	
1526	CHECK 1505A (a-j), 1515A (a,b), 1516, 1520, 1522A, AND 1522B:<br>AT LEAST ONE 'YES' ☐ ↓ | NOT A SINGLE 'YES' ☐ →		1530
1527	Thinking about what you yourself have experienced among the different things we have been talking about, have you ever tried to seek help?	YES . . . . . 1<br>NO 2	2 → 1529

NO.	QUESTIONS AND FILTERS	CODING CATEGORIES	SKIP
1528	From whom have you sought help?<br><br>Anyone else?<br><br>RECORD ALL MENTIONED.	OWN FAMILY . . . . . A<br>HUSBAND'S/PARTNER'S FAMILY . . . . . B<br>CURRENT/FORMER HUSBAND/PARTNER . . . . . C<br>CURRENT/FORMER BOYFRIEND . . . . . D<br>FRIEND . . . . . E<br>NEIGHBOR . . . . . F<br>RELIGIOUS LEADER . . . . . G<br>DOCTOR/MEDICAL PERSONNEL . . . . . H<br>POLICE . . . . . I<br>LAWYER . . . . . J<br>SOCIAL SERVICE ORGANIZATION . . . . . K<br><br>OTHER ______ X<br>(SPECIFY)	→ 1530
1529	Have you ever told any one about this?	YES . . . . . 1<br>NO 2	
1530	As far as you know, did your father ever beat your mother?	YES . . . . . 1<br>NO . . . . . 2<br>DON'T KNOW 8	
	THANK THE RESPONDENT FOR HER COOPERATION AND REASSURE HER ABOUT THE CONFIDENTIALITY OF HER ANSWERS. FILL OUT THE QUESTIONS BELOW WITH REFERENCE TO THE DOMESTIC VIOLENCE MODULE ONLY.		

NO.	QUESTION		YES, ONCE	YES, MORE THAN ONCE	NO
1531	DID YOU HAVE TO INTERRUPT THE INTERVIEW BECAUSE SOME ADULT WAS TRYING TO LISTEN, OR CAME INTO THE ROOM, OR INTERFERED IN ANY OTHER WAY?	HUSBAND	1	2	3
		OTHER MALE ADU[illegible]	1	2	3
		FEMALE ADUL[illegible]	1	2	3

NO.	QUESTIONS AND FILTERS	CODING CATEGORIES	SKIP
1532	INTERVIEWER'S COMMENTS/EXPLANATION FOR NOT COMPLETING THE DOMESTIC VIOLENCE MODULE.<br>______<br>______<br>______		
1533	CHECK 223A:<br>ONE OR MORE DEATHS ☐ ↓	NO DEATHS ☐	→ 1535
1534	READ TO THE RESPONDENT:<br>I would like to inform you that detailed information on the circumstances surrounding the deaths of children under the age of 5 years will be collected in the near future so that the federal government of Nigeria can provide health services to help reduce these deaths. If you do not mind, another team will be coming at a leter date to interview members of the household about the death (s) you have told me about. Is this okay?	YES . . . . . 1<br><br>NO 2	
1535	RECORD THE TIME.	HOURS ☐☐<br>MINUTES ☐☐	

INTERVIEWER'S OBSERVATIONS

TO BE FILLED IN AFTER COMPLETING INTERVIEW

COMMENTS ABOUT INTERVIEW:

COMMENTS ON SPECIFIC QUESTIONS:

ANY OTHER COMMENTS:

SUPERVISOR'S OBSERVATIONS

EDITOR'S OBSERVATIONS

INSTRUCTIONS:

ONLY ONE CODE SHOULD APPEAR IN ANY BOX.
COLUMN 1 REQUIRES A CODE IN EVERY MONTH.

CODES FOR EACH COLUMN:

COLUMN 1: <u>BIRTHS, PREGNANCIES, CONTRACEPTIVE USE</u> (2)

B BIRTHS
P PREGNANCIES
T TERMINATIONS

0 NO METHOD

1 FEMALE STERILIZATION
2 MALE STERILIZATION
3 IUD
4 INJECTABLES
5 IMPLANTS
6 PILL
7 CONDOM
8 FEMALE CONDOM
9 EMERGENCY CONTRACEPTION
J STANDARD DAYS METHOD
K LACTATIONAL AMENORRHEA METHOD
L RHYTHM METHOD

M WITHDRAWAL
X OTHER MODERN METHOD
Y OTHER TRADITIONAL METHOD

COLUMN 2: <u>DISCONTINUATION OF CONTRACEPTIVE USE</u>

0 INFREQUENT SEX/HUSBAND AWAY
1 BECAME PREGNANT WHILE USING
2 WANTED TO BECOME PREGNANT
3 HUSBAND/PARTNER DISAPPROVED
4 WANTED MORE EFFECTIVE METHOD
5 SIDE EFFECTS/HEALTH CONCERNS

6 LACK OF ACCESS/TOO FAR
7 COSTS TOO MUCH
8 INCONVENIENT TO USE
F UP TO GOD/FATALISTIC
A DIFFICULT TO GET PREGNANT/MENOPAUSAL
D MARITAL DISSOLUTION/SEPARATION
X OTHER

____
(SPECIFY)

Z DON'T KNOW

Year				COL. 1	COL. 2	Year
	02	FEB	01			2019
	01	JAN	02			
2018	12	DEC	03			2018
	11	NOV	04			
	10	OCT	05			
	09	SEP	06			
	08	AUG	07			
	07	JUL	08			
	06	JUN	09			
	05	MAY	10			
	04	APR	11			
	03	MAR	12			
	02	FEB	13			
	01	JAN	14			
2017	12	DEC	15			2017
	11	NOV	16			
	10	OCT	17			
	09	SEP	18			
	08	AUG	19			
	07	JUL	20			
	06	JUN	21			
	05	MAY	22			
	04	APR	23			
	03	MAR	24			
	02	FEB	25			
	01	JAN	26			
2016	12	DEC	27			2016
	11	NOV	28			
	10	OCT	29			
	09	SEP	30			
	08	AUG	31			
	07	JUL	32			
	06	JUN	33			
	05	MAY	34			
	04	APR	35			
	03	MAR	36			
	02	FEB	37			
	01	JAN	38			
2015	12	DEC	39			2015
	11	NOV	40			
	10	OCT	41			
	09	SEP	42			
	08	AUG	43			
	07	JUL	44			
	06	JUN	45			
	05	MAY	46			
	04	APR	47			
	03	MAR	48			
	02	FEB	49			
	01	JAN	50			
2014	12	DEC	51			2014
	11	NOV	52			
	10	OCT	53			
	09	SEP	54			
	08	AUG	55			
	07	JUL	56			
	06	JUN	57			
	05	MAY	58			
	04	APR	59			
	03	MAR	60			
	02	FEB	61			
	01	JAN	62			
2013	12	DEC	63			2013
	11	NOV	64			
	10	OCT	65			
	09	SEP	66			
	08	AUG	67			
	07	JUL	68			
	06	JUN	69			
	05	MAY	70			
	04	APR	71			
	03	MAR	72			
	02	FEB	73			
	01	JAN	74			
